# A Multicenter Cost-of-Illness and Long-term Socioeconomic Follow-up Study in the Severe Typhoid Fever in Africa Program: Study Protocol

**DOI:** 10.1093/cid/ciz608

**Published:** 2019-10-30

**Authors:** Enusa Ramani, Seeun Park, Trevor Toy, Ursula Panzner, Ondari D Mogeni, Justin Im, Ligia Maria Cruz Espinoza, Hyon Jin Jeon, Gi Deok Pak, Hyeongwon Seo, Yun Chon, Raphaël Rakotozandrindrainy, Ellis Owusu-Dabo, Isaac Osei, Abdramane Bassiahi Soura, Mekonnen Teferi, Florian Marks, Vittal Mogasale

**Affiliations:** 1 Policy and Economic Research Department, Development and Delivery Unit, International Vaccine Institute (IVI), Seoul National University (SNU) Research Park, Republic of Korea; 2 Department of Health Care Management, Faculty of Economics and Management, Berlin University of Technology, Germany; 3 Public Health, Access, and Vaccine Epidemiology Unit, IVI, SNU Research Park, Seoul, Republic of Korea; 4 Oxford University Clinical Research Unit, Ho Chi Minh City, Vietnam; 5 Department of Medicine, University of Cambridge, United Kingdom; 6 Department of Biostatistics and Data Management, Development and Delivery Unit, IVI, SNU Research Park, Seoul, Republic of Korea; 7 University of Antananarivo, Madagascar; 8 School of Public Health, and, Kumasi, Ghana; 9 Kumasi Centre for Collaborative Research in Tropical Medicine, Kwame Nkrumah University of Science and Technology, Kumasi, Ghana; 10 Institut Supérieur des Sciences de la Population, University of Ouagadougou, Burkina Faso; 11 Armauer Hansen Research Institute, ALERT Campus, Addis Ababa, Ethiopia

**Keywords:** cost of illness, socioeconomics, enteric fever, typhoid fever, Africa

## Abstract

**Background:**

There are limited data on typhoid fever cost of illness (COI) and economic impact from Africa. Health economic data are essential for measuring the cost-effectiveness of vaccination or other disease control interventions. Here, we describe the protocol and methods for conducting the health economic studies under the Severe Typhoid Fever in Africa (SETA) program.

**Methods:**

The SETA health economic studies will rely on the platform for SETA typhoid surveillance in 4 African countries—Burkina Faso, Ethiopia, Ghana, and Madagascar. A COI and long-term socioeconomic study (LT-SES) will be its components. The COI will be assessed among blood culture–positive typhoid fever cases, blood culture–negative clinically suspected cases (clinical cases), and typhoid fever cases with pathognomonic gastrointestinal perforations (special cases). Repeated surveys using pretested questionnaires will be used to measure out-of-pocket expenses, quality of life, and the long-term socioeconomic impact. The cost of resources consumed for diagnosis and treatment will be collected at health facilities.

**Results:**

Results from these studies will be published in peer-reviewed journals and presented at scientific conferences to make the data available to the wider health economics and public health research communities.

**Conclusions:**

The health economic data will be analyzed to estimate the average cost per case, the quality of life at different stages of illness, financial stress due to illness, and the burden on the family due to caregiving during illness. The data generated are expected to be used in economic analysis and policy making on typhoid control interventions in sub-Saharan Africa.

The economic burden of disease is a summary estimate of the extent to which a society is economically stretched in managing a particular illness [1]. Out-of-pocket (OOP) payment is the term for the amount paid for the treatment of illness by patients and their household that impacts their finances directly. Both affected persons and caretakers lose income or their potential opportunity to earn income as the inability to perform their regular work impacts finances indirectly. A healthcare facility spends resources to deliver treatment for an illness, and the costs borne by the public health facility represent treatment costs borne by the government. A cost-of-illness (COI) study is used to determine how much each of the parties in society, including the patient and/or their household, the government, and third-party payers such as insurers, nongovernmental organizations, or philanthropies, spend as a result of the illness.

A COI study specific for typhoid fever can be used to estimate health facility costs and OOP payments for treatment and associated productivity losses and thereby enables the estimation of costs that can be saved by typhoid fever control measures. Much effort has been made to quantify typhoid fever incidence [2–7], but the same is not true for collecting economic burden data. To date, only 3 studies have estimated the COI for typhoid fever, 2 from Asia [8, 9] and 1 from Africa [10]. The study from Africa focused on a single location (Zanzibar island) and had a sample size of 17 laboratory-confirmed typhoid fever cases. One study with a small sample size cannot be extrapolated to the entire African region. There is a need to conduct field studies in Africa to assess typhoid fever COI, which can be used in estimating and comparing cost-effectiveness of various typhoid control measures. Such economic evidence is essential for making policy and programmatic decisions, priority setting, and resource allocation. The recent World Health Organization [11] prequalification of a typhoid conjugate vaccine (TCV) and financial commitments by Gavi, the Vaccine Alliance for TCV introduction in eligible countries make cost-effectiveness analysis in African settings an important need.

Furthermore, the long-term costs of complicated typhoid fever, such as intestinal perforation and its resultant socioeconomic implications, are not understood. Patients with complicated cases are known to stay in hospital for longer periods of time and are recognized to have a higher mortality. A systematic review of studies published from 1991 to 2011 revealed a mortality ratio of 15.5% in hospitalized cases with intestinal perforation [12]. Descriptive data from hospital-based studies in Africa demonstrate great variability in clinical outcomes and organ systems where typhoid fever complications manifest [13–16]. Conditions such as intestinal hemorrhage and perforation may require surgical intervention and contribute significantly to the costs incurred [11].

The Typhoid Fever Surveillance in Africa Program (TSAP) generated standardized data on typhoid fever incidence, using a standardized blood culture–based approach, in 10 sub-Saharan Africa countries to be used for evidence-based decision making related to enteric fever control measures and prevention, including vaccination strategies [5, 11, 17]. It therefore became important to follow up typhoid fever cases to measure frequency of various complications, long-term sequelae, and socioeconomic consequences that can occur during the course of illness to understand real disease burden. Consequently, TSAP was succeeded by the Severe Typhoid Fever in Africa (SETA) program. SETA implemented a surveillance program that will allow the collection of the information related to typhoid fever incidence as well as complications in several sites across sub-Saharan Africa by following up cases up to 360 days [18]. The program will establish surveillance for febrile illness at tertiary, secondary, and primary healthcare facilities where clinical and microbiological data will be collected from participants with suspected typhoid fever and febrile participants with invasive nontyphoidal *Salmonella* (iNTS). The SETA research program, described elsewhere [18], will provide an opportunity to conduct COI and long-term socioeconomic studies (LT-SES) in Africa by sharing a common platform and resources used for typhoid fever surveillance. Typhoid fever cases identified through surveillance will serve as cohort participants for these 2 studies.

## METHODS

There will be 2 health economics study components under the SETA program: COI and LT-SES. Costing will be conducted from the societal perspective using the micro-costing approach. Through this approach, also known as bottom-up or ingredient costing, all input resources in the treatment of illness by health facilities and the OOP payments by patients and their household will be identified, measured, and valued.

### Cost-of-Illness Study

**Figure 1.**
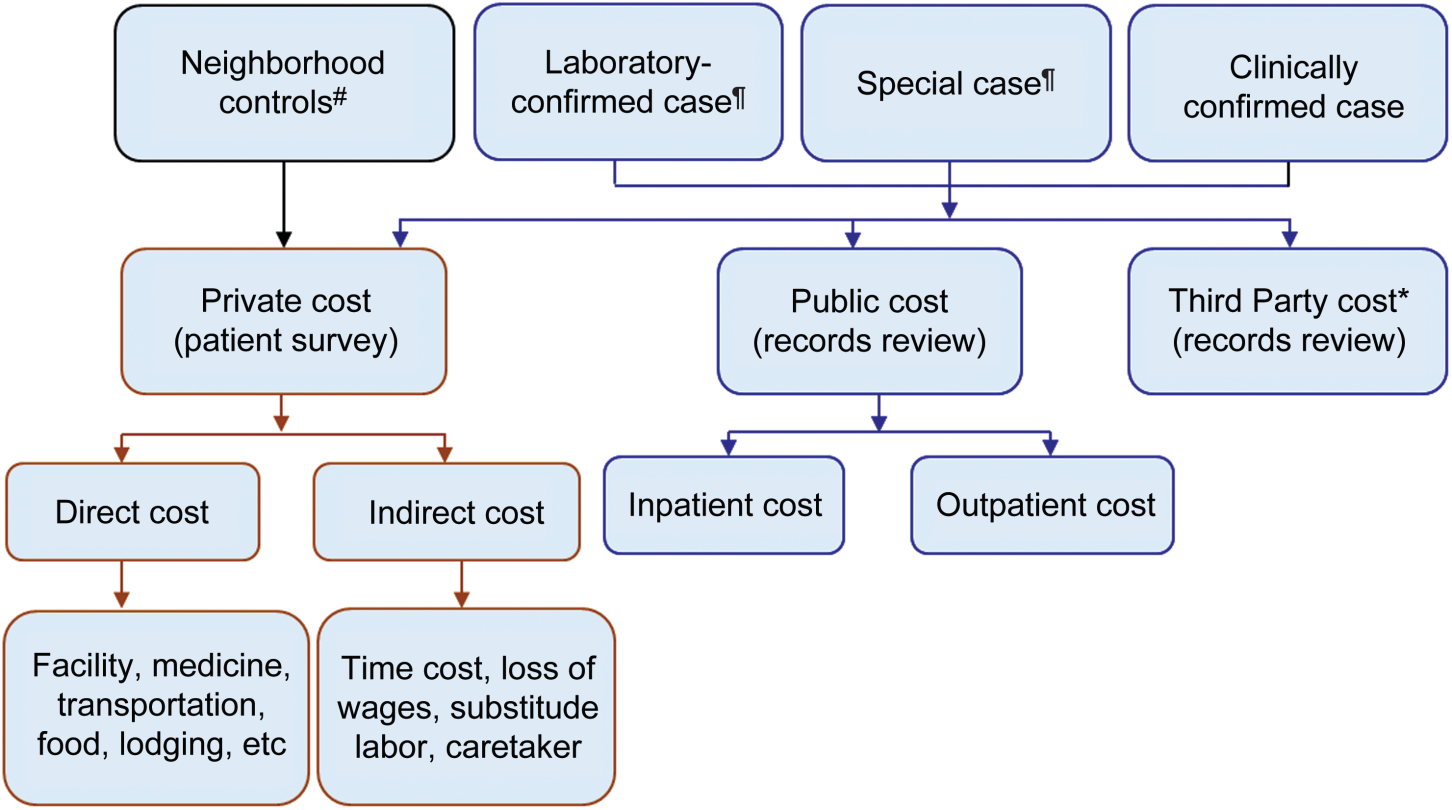
Cost-of-illness study design in the Severe Typhoid Fever in Africa program. Cost of death is to be collected in the event of participant’s passing. ^**#**^Private cost of other illnesses apart from typhoid fever (TF) will be collected. ^**¶**^Private cost of other illnesses will be collected in addition to that of TF. *To be collected only if applicable.

The COI study will have 2 subcomponents: patient costs and health facility costs. The patient cost component will aim at estimating OOP payments and productivity losses borne by patient and households due to typhoid fever illness using the COI survey tools (Supplementary Annexes 1 and 2). The health facility costing will aim to estimate the health facility expenditure on delivering health service based on a review of health facility records (Figure 1). In some countries, health facility costs may be shared by third parties such as insurers.

The patient costs will be estimated based on interviews of typhoid cases, hereafter referred to as participants, and their caregivers at various time points as described in the schedule below (Table 1) until the self-report of the end of illness. The estimates will be based on self-reported expenditures. All direct medical costs arising out of physician consultation, medicines, laboratory diagnostics and radiological services, and direct nonmedical expenditure/costs caused by travel and food purchase that have resulted from participants’ illness and consequential treatment will be valued. The interview will capture indirect costs arising from productivity or income loss to participants and their caregivers during illness. The duration of time absent from work or other activities, time lost by caretakers in caring for the sick, and the amount of time needed to substitute labor will be estimated with the equivalent monetary value for lost time, conservatively based on the minimum wage per time unit. To assess the background healthcare costs, a separate COI tool called the cost of other illness (COOI) (Supplementary Annexes 3 and 4) will be used. The COOI is similar to the COI tool and collects cost for illnesses other than typhoid fever. To understand baseline healthcare expenditures, we will also interview neighborhood controls selected as part of the SETA study [18]. The tools developed/used for collecting patient and household costs were derived from illness costing tools designed and used previously by the International Vaccine Institute [19] in many study sites in Asia and Africa [9, 10].

**Table 1.** Schedule of Follow-up

Day	Laboratory/Special Cases	Clinical Cases	Control
Day 0	Consent + QoL	Consent + QoL	Consent + QoL
Day 3–7	Interview 1 (COI + COOI + LT-SES)^a^	Interview 1 (COI)	Interview 1 (COOI + LT-SES)
Day 12–14	Interview 2 (COI + COOI + LT-SES)	Interview 2 (COI)	Interview 2 (COOI + LT-SES)
Day 28–30	Interview 3 (COI + COOI + LT-SES)	Interview 3 (COI)	Interview 3 (COOI + LT-SES)
Day 90 ± 7	Interview 4 (COI + COOI + LT-SES)^b^	Interview 4 (COI)^b^	Interview 4 (COOI + LT-SES)^b^
Day 180 ± 7	Interview 5 (LT-SES)	…	Interview 5 (LT-SES)
Day 270 ± 7	Interview 6 (LT-SES)	…	Interview 6 (LT-SES)
Day 360 ± 7	Interview 7 (LT-SES)	…	Interview 7 (LT-SES)

If any study participant dies upon enrollment or during follow-up, the death-related costs due to illness tool will be utilized to capture all funeral-related costs.

Abbreviations: COI, cost of illness; COOI, cost of other illness; LT-SES, long-tern socioeconomic study; QoL, quality of life.

^a^Special case may have interview 1 on enrollment day.

^b^COI and COOI survey may continue if illness persists.

Health facility costs arising out of health services delivery will be estimated based on interviews conducted among health services staff and review of financial records at study health facilities. The cost estimation will include unit costs for items such as medicines, laboratory clinical procedures, and time costs of health professionals. We will also collect utility costs of each study healthcare facility for electricity and water if available. Capital costs for building/infrastructure, equipment, and maintenance will also be collected where possible. The costs will be estimated using mainly the bottom-up approach based on inventory and financial reports at health facilities and elsewhere. In addition, the time-motion study—a process of detailed observation of each health professional involved in the treatment procedure, using a stopwatch to evaluate the time needed to complete a specific task—will be conducted [20]. The tools for collecting health facility costs will be developed based on published works by Stephen Morris [21] and by Michael Drummond and colleagues [22] on valuing unit health services costs.

### Long-term Socioeconomic Study

The LT-SES will assess the short-term and long-term health and economic consequences of typhoid fever for participants and their households. The LT-SES will have 3 subcomponents: quality of life (QoL), financial burden, and family burden. A separate tool will be utilized for each subcomponent (Supplementary Annexes 5–8). The QoL instrument will assess the well-being of participants at the time of study enrollment and each of the subsequent interviews so that change can be tracked over time. The financial burden instrument will evaluate the level of financial stress exerted on the participants and their families. The family burden instrument will be used to measure the disruption of social, physical, psychological, and general well-being of caretakers of participants. The LT-SES study will involve interview of participants and/or their caretakers periodically on multiple occasions over a 360-day period from the time of their typhoid fever diagnosis and enrollment, as described under the follow-up schedule in Table 1. To understand the baseline socioeconomic burden, neighborhood controls and febrile participants identified through the SETA study [18] will be interviewed using the QoL and LT-SES tools at the same time points as the typhoid fever participants.

The QoL tool was adapted from the RAND 36-Item Short-Form Survey [23], which was tested, validated, and used in many countries, including some African countries. The QoL tool will measure 8 concepts of well-being: (1) physical functioning, (2) bodily pain, (3) role limitation due to physical health problems, (4) role limitation due to personal or emotional problems, (5) emotional well-being, (6) social functioning, (7) energy or fatigue, and (8) general health perceptions. The financial burden tool adapted for the study has been used in countries in Asia [9], Africa [10], and Latin America [19]. The financial burden section of the tool will measure the level of borrowing, sale of property/belongings due to treatment, and withdrawal from treatment due to shortage of funds. The family burden tool was developed from the family burden interview schedule [24, 25] used in many countries across the globe and was adapted for this study. The family burden interview schedule section will measure the subjective feeling of the caregiver on any (1) financial repercussions, (2) disruption of planned family routine activity, (3) impact on mental health of others, and (4) subjective burden on the family of the participant.

We will also collect costs related to participants’ typhoid fever–related death from the time of death until the funeral by interviewing close relatives who were involved in taking care of a participant during the illness.

### Study Arms

The SETA COI study will have 4 main arms: laboratory-confirmed cases, special cases (complicated typhoid fever cases with pathognomonic gastrointestinal perforations), clinical cases, and neighborhood control arms (Table 2). Participants whose blood culture tests positive for *Salmonella enterica* serovar Typhi will make up the laboratory-confirmed arm. Those who have clinically suspected typhoid fever with pathognomonic gastrointestinal perforations as defined in the SETA protocol [18] will form the special case arm. These 2 arms will be administered with the COI and the COOI tools. Those clinically suspected with typhoid fever, but with a blood culture negative for *S*. Typhi, will form the clinical case arm; for this group, only COI tools will be administered. Neighborhood controls will be the individuals who are healthy at the focal point (the time when the respective laboratory-confirmed typhoid fever case is enrolled) and who live in the same neighborhood. For this arm, only the COOI tools will be administered to capture any expenditure incurred for the treatment of other illnesses. The relapse and reinfection as defined in the SETA protocol will be followed in COI and LT-SES studies too [18].

**Table 2.** Inclusion Criteria

Laboratory-confirmed typhoid fever cases
Participants who fulfill the following criteria will be included for typhoid fever case follow-up: 1. Enrolled in SETA surveillance as febrile case [18], AND 2. Confirmed blood culture positive for *Salmonella* Typhi, AND 3. Confirmation of SETA surveillance consent
Special cases
Participants who fulfill the following criteria will be included for special case follow-up: 1. Enrolled in SETA surveillance as special case (defined as pathognomonic gastrointestinal perforations, ie, clinically diagnosed typhoid fever gastrointestinal perforation), even in the absence of laboratory confirmation, in patients living in and outside the defined catchment area, AND 2. The blood culture is either negative or positive for typhoid fever, but the consulted surgeon(s) clinically diagnosed typhoid fever intestinal perforation and there is no other confirmed cause of perforation, AND 3. Patient undergoes surgical procedure and intestinal perforation is confirmed, AND 4. Confirmation of SETA surveillance consent
Clinical cases
Participants who fulfill the following criteria will be followed up as clinical cases: 1. Enrolled in SETA surveillance study as febrile case, AND 2. Clinician checked “typhoid/enteric fever” under subsection F of section V for preliminary diagnosis in SETA surveillance case report form Part 1, Question 23F (Supplementary Annex 10). 3. Confirmed blood culture negative for *S*. Typhi, AND 4. Duration of illness, place of residence, age and sex matched, and enrolled subsequent to index laboratory-confirmed case (duration from onset of fever ±1 day [preferred], ±2 days [second choice], ±3 days [third choice], ±4 days [fourth choice], ±5 days [fifth choice]; as well as age ±1 [preferred], ±2 [second choice], ±3 [third choice], ±4 [fourth choice], ±5 [fifth choice] years if case is <15 years old and ±5 years if case is ≥15 years old) 5. Confirmation of SETA surveillance consent
Neighborhood controls
Participants who fulfill the following criteria will be included for neighborhood control follow-up: 1. Enrolled in SETA surveillance study as control participant for the index case, AND 2. NO subjective or objective fever reported at any point within 28 days prior to the date of enrollment, AND 3. NO subjective or objective fever on the date of case enrollment (“focal time”), AND 4. Place of residence, age and sex matched, and enrolled subsequent to index laboratory-confirmed case (duration from onset of fever ±1 day [preferred], ±2 days [second choice], ±3 days [third choice], ±4 days [fourth choice], ±5 days [fifth choice]; as well as age ±1 [preferred], ±2 [second choice], ±3 [third choice], ±4 [fourth choice], ±5 [fifth choice] years if case is <15 years old and ±5 years if case is ≥15 years old), AND 5. Residency in the catchment area of the SETA healthcare facility, AND 6. Confirmation of SETA surveillance consent

Abbreviation: SETA, Severe Typhoid Fever in Africa.

Each clinical case will be matched 1:1 to a laboratory-confirmed case based on the potential cost drivers—duration of fever, the place of residence (neighborhood), age (±5 years), and sex—so that the costs between clinical and laboratory cases can be compared. The neighborhood controls will be matched 4:1 to each laboratory-confirmed case and special case by age (±5 years), sex, and residency (neighborhood) and will be enrolled within 7 days of the confirmation of a case (at most 10 days from the date of case enrollment into SETA).

Those who are blood culture confirmed for iNTS or *Salmonella enterica* serovar Paratyphi (A/B/C) will form a single-arm COI study and will be interviewed using the COI tools only but following the same interview procedure as the laboratory-confirmed typhoid fever cases.

The LT-SES will have 3 arms: laboratory-confirmed cases, special cases, and neighborhood controls. The socioeconomic impact of illness will be assessed over a period of 360 days. The QoL, LT-SES, and COOI tools will be administered to all 3 arms.

### Study Follow-up Schedule

**Figure 2.**
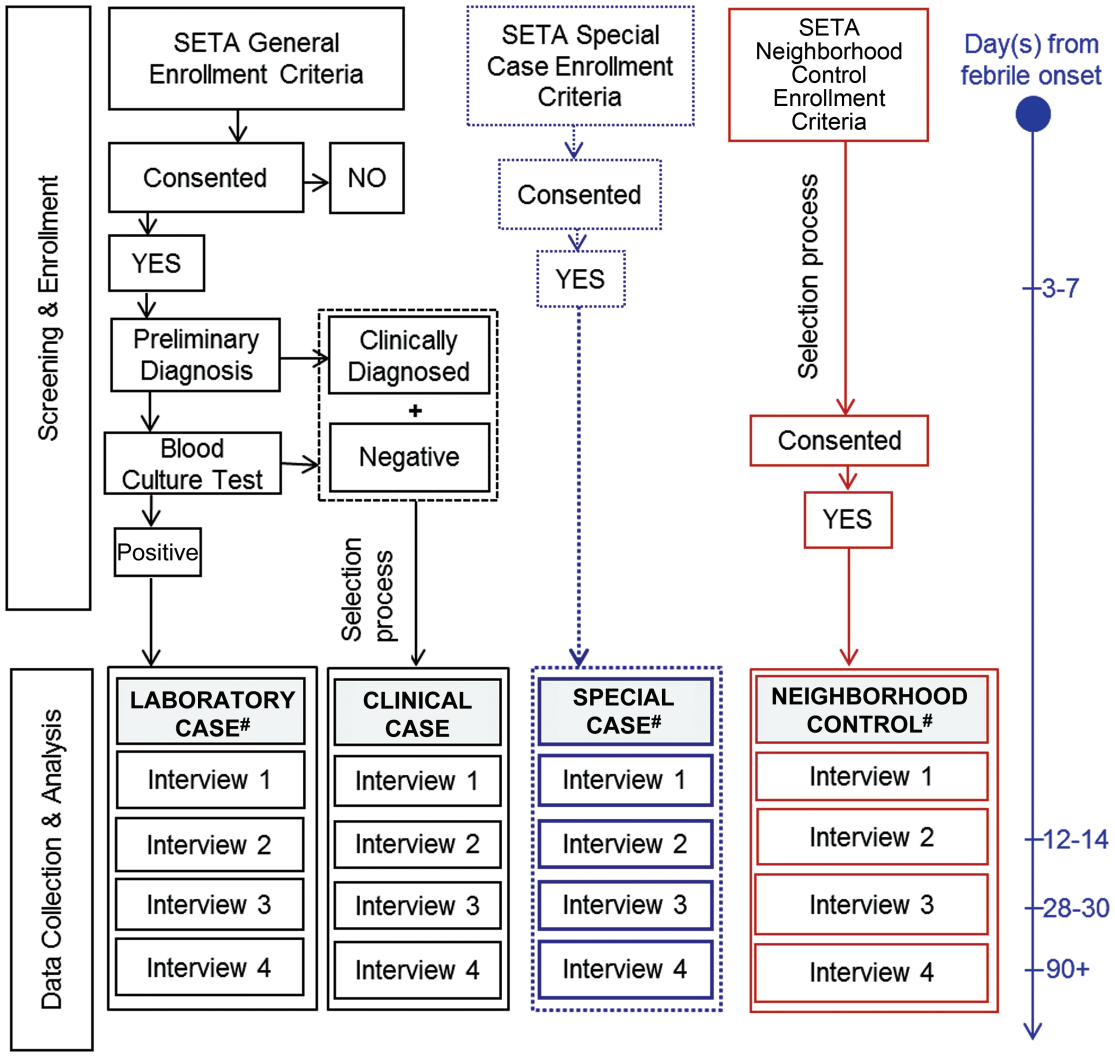
Data collection procedure for patient and household costs. ^#^Continues into long-term socioeconomic study. Abbreviation: SETA, Severe Typhoid Fever in Africa program.

At the time of first presentation to a SETA healthcare facility, blood will be taken from the enrolled febrile participant. This time point will be considered as day zero. The QoL tool will be administered on day zero for all participants. For all laboratory-confirmed cases, special cases, and clinical cases, the COI interview will be conducted upon the earliest availability of their microbiologic culture test results or inclusion confirmation in the case of special cases (Figure 2). This will be considered the first interview, which is expected to occur 3–7 days after day zero. A second interview will be conducted within 12–14 days from day zero. If the participant continues to feel sick, a third interview will be conducted 28–30 days from day zero. We expect most participants to have 2–3 interviews; however, if participants continue to report illness, more interviews will be conducted until they affirm to be well or until 360 days (Table 1). The neighborhood controls will be interviewed in the same manner as above using the respective tools as described before.

For the 3 arms under the LT-SES (laboratory-confirmed case, special case, and neighborhood controls), the QoL survey will be administered on day zero and the LT-SES questionnaire will be administered 7 times up to day 360 as per the follow-up schedule in Table 1.

### Data Collection Procedure

The data on OOP and productivity loss related to illness will be collected by trained field surveyors by prescheduled face-to-face interview either at the health facility or at the participants’ places of residence using the patient and household costing tools (COI and COOI). The data will be collected together with additional surveillance data during scheduled visits. If the field surveyors fail to locate a participant on 3 consecutive visits, the participant will be considered as lost to follow-up from the study. Health service delivery costs will be collected by reviewing hospital records and interviewing some health authorities using a pretested health facility costing tool. Data on QoL, financial burden, and family burden will also be collected by the field surveyors through face-to-face interview matching COI or surveillance visits. In case of a study participant’s demise resulting from typhoid fever, a cost-of-death tool (Supplementary Annex 9) will be administered by the field surveyors to collect all funeral-related costs due to the illness under study.

### Data Protection

Identifying information for all study participants will be kept separately from all study materials, and participants will be indicated by a unique study label. Files linking the participants’ identifying information will be accessible only by the principal investigator or study coordinator and made available to authorized field surveyors when necessary. Identifying information for selected participants will be retrieved from the SETA surveillance database to conduct follow-up visits.

### Data Analysis and Results Presentation

Data collected from the field sites will be entered into the EpiCollect5 (Android 1.0 or 1.5), digital database designed for this study, using hand-held tablets [26, 27]. Participants’ profile information will not be collected by the health economic forms but will be paired from the SETA surveillance database by means of study linkage numbers. Stata software version 14.2 (StataCorp, College Station, Texas) [28] will be used for data exploration, cleaning, and preparation for analyses. All cost data will be adjusted by (1) converting local currency units to US dollar rates for base year (various years of data collection); (2) applying inflation rates from base year to the year of analysis; and (3) converting all estimated costs to the international dollar (purchasing power parity) rate for comparability across all study countries.

The COI for laboratory-confirmed cases and special cases will be estimated based on data collected; the average cost per typhoid fever case will be calculated and categorized by age group (children, adults), type of hospital service used (outpatients, inpatients), who spends it (health facility, patient, and households), and country. Productivity losses will be analyzed taking into consideration the duration of time participants and their caretakers lost in monetary terms to arrive at the average costs of productivity loss due to illness. We will avoid double-counting the number and duration a substitute laborer’s services were enlisted. Healthcare facility costs will be analyzed by accounting for health service delivery personnel cost, materials and supplies cost, medical and nonmedical equipment cost, medication cost, cost of facility building or rent, and estimation of the monetary value of the proportion of each needed in treatment of each episode of typhoid fever. The cost of death will also be analyzed by summing the amount of money spent from the time of death of the participant to the funeral.

The cost of other illnesses, data collected for laboratory-confirmed typhoid fever cases and neighborhood controls, will be analyzed and compared to assess cost biases related to typhoid fever healthcare seeking. Similarly, cost of other illness will be compared between special cases and neighborhood controls.

Under the LT-SES, data on QoL and level of financial and caretaker burden for typhoid fever/special cases and that of their matched neighborhood controls will be assessed over time. Multivariate analysis of the socioeconomic indicators of typhoid fever illness will be conducted to assess its socioeconomic impact over periods beyond illness or by prolonged illness. Changes in caretaker burden, social relations, and financial commitments toward treatment of typhoid fever and its complications as well as quality-adjusted life-years will be estimated from the responses of the QoL and LT-SES surveys. Methods recommended by RAND Health Care [29] will be used for analyzing the QoL data.

### Publication of Results

Results from these studies will be published in peer-reviewed journals and presented at scientific conferences and seminars to make the data on typhoid fever COI and LT-SES available to the wider health economics, infectious diseases, and public health research communities. The data from the COI studies are most useful in conducting cost-effectiveness analysis of interventions for typhoid fever control such as TCV deployment. These economic analyses incorporating disease burden, economic burden, program costs, and potential effectiveness of interventions such TCV are crucial evidence needed for policy and vaccine introduction decisions. This will be particularly important in the near future as World Health Organization–prequalified TCV is available for use in low- and middle-income countries and Gavi has made commitment for its use.

### Study Sites and Ethical Considerations

The COI and the LT-SES will be implemented at several sites in 4 SETA program countries: Burkina Faso, Ethiopia, Ghana, and Madagascar. All ethical approvals for this study were solicited from the Institutional Review Board of the International Vaccine Institute and the Ethical Commissions of the respective study countries along with overall approvals for SETA [18]. Informed consent for participation in the COI and surveillance studies is obtained concurrently from participants during the SETA enrollment. For young adults aged 12–17 years, written assent is required in addition to informed consent from a parent/guardian. For minors aged <12 years, consent will be obtained from their parents or caretakers.

## Supplementary Data

Supplementary materials are available at *Clinical Infectious Diseases* online. Consisting of data provided by the authors to benefit the reader, the posted materials are not copyedited and are the sole responsibility of the authors, so questions or comments should be addressed to the corresponding author.

ciz608_suppl_SETA_COI_Annex_2018

## Notes

***Author contributions.*** V. M. and E. R. conceptualized the study design and facilitated the study implementation with partners. S. E. P., T. T., U. P., O. D. M., J. I., H. J., G. D. P., H. W. S., Y. C., R. R., E. O. D., I. O., A. B. S., M. T., L. M. C. E., and F. M. participated in the study design and implementation. V. M. and E. R. wrote the first draft of the protocol and subsequently modified it with the contribution of all coauthors. All authors and coauthors agreed to the final content. All authors critically reviewed and approved the final manuscript.

***Acknowledgments.*** The authors thank the ad hoc Policy and Economic Research Advisory Committee members Dr Wilm Quintin, Prof Dr Reinhard Busse, Dr Christopher Parry, Dr Raymond Hutubessy, and Prof Dr Christine Faeber, who were instrumental in advising on the study design. The authors also extend appreciation to Soo-Young Kwon and Ji Hyun Han of the International Vaccine Institute for their administrative support.

***Financial support*.** This research was funded by the Bill & Melinda Gates Foundation [OPP1127988]. The International Vaccine Institute acknowledges its donors including the Republic of Korea and the Swedish International Development Cooperation Agency. This publication was made possible through a grant from the Bill & Melinda Gates Foundation [OPP1201031].

***Supplement sponsorship*.** This article was published as part of the supplement “Severe Typhoid Fever in Africa (SETA) Program” sponsored by the International Vaccine Institute.

***Potential conflicts of interest.*** The authors report no potential conflicts of interest. All authors have submitted the ICMJE Form for Disclosure of Potential Conflicts of Interest. Conflicts that the editors consider relevant to the content of the manuscript have been disclosed.
