# Supplementary material for: A Multicenter Cost-of-Illness and Long-term Socioeconomic Follow-up Study in the Severe Typhoid Fever in Africa Program: Study Protocol
Source: Clin Infect Dis. 2019 Oct 30;69(Suppl 6):S459–65. doi: 10.1093/cid/ciz608 (PMC6821243; doi:10.1093/cid/ciz608)
Supplement: ciz608_suppl_SETA_COI_Annex_2018 [file ciz608_suppl_seta_coi_annex_2018.pdf]

Form S.I.#:__________________ **Study label** | | | | | | | | | | |

(To be filled by Data Management)

Annex 1: SETA COI Initial Interview Tool

Form F2: Cost of Illness (COI) Initial Interview

(Day 0 and Day 3 to 7: Upon enrollment of special cases OR 1 week post enrollment)

F2 for *S*. Typhi or Special or *S*. Paratyphi or iNTS positive and clinically diagnosed laboratory negative cases

Note to Interviewer
*If you see **"Ç",** that is a **note for you** to be read by yourself. If you see **"☺"** you should **read out loud** for the respondent or the caretaker/Next of kin.*

Site/**C**ountry: ______________________________/______________________________

Follow-up Schedule: ☐Day 0 (for special cases only) ☐Day 3-7

Interviewer's Name:__________________________ **D**ate of Interview(dd/mm/yyyy):_____/_____/__________

Laboratory diagnosis: ☐*S.* Typhi ☐Special Case ☐*S.* Paratyphi ☐iNTS ☐**Cl**inical enteric fever

Place of interview: ☐Health facility / ☐Home / ☐Other, specify: _________________________

If at health facility, indicate point of care: ☐Out-Patient / ☐In-Patient / ☐Invited visit for SETA research

If In-Patient, name of admission ward: _________________________

Date of Admission (dd/mm/yyyy): _____/_____/__________

Date of Discharge (dd/mm/yyyy): _____/_____/__________ [**Ç:** Look this up in the study facility record]

COI Questionnaire: Day 3-7 (Upon availability of diagnosis results)

Ç: *Is the participant the right person interviewed 3-7 days ago?*		
0	Correct?	☐1.Yes ☐2.No ☐9.Don't know
Ç: *If "**No**" or "**Don't know**", identify the right candidate to continue the interview.*		

Ç: *Use this instrument to interview an adult participant/caretaker/Next of kin(in case of a minor or if health-impaired) that is familiar with the participant and his/her treatment over the last few days.*
☺ Now I am going to ask you about the participant's recent illness and the costs that the participant/their household incurred for management of the illness until yesterday.

Form S.I.#:___________________

(To be filled by Data Management)

Study label | | | | | | | | | | |

A	☺ Now I want to know a little bit about you, any health insurance you might have and the duration of your health condition before and after the visit to health facility. I need this information to help me estimate a reliable cost of illness.	
1	Could you please tell me your relationship to the participant? ***(Ç: If interviewing participant directly, check response 1)***	☐1.Participant ☐2.Spouse ☐3.Mother ☐4.Father<br>☐5.Brother ☐6.Sister ☐7.Grandfather<br>☐8.Grandmother ☐9.Uncle ☐10.Aunt<br>☐11.Cousin ☐98.Other, specify: ____________________
2	How many days was the participant sick with the disease **before visiting** [enter facility/clinic name: ______________________________] where blood sample was collected from him/her for diagnosis OR the day participant was enrolled to the study (*for special cases*)?	Days: __________<br>99=Don't know
3	How many days was the participant sick with the disease **after visiting** [enter facility/clinic name: ______________________________] where blood sample was collected from him/her for diagnosis including the day of visit OR the day participant was enrolled to the study (*for special cases*)?<br>***(Ç: Please remember to include the day of the facility visit in the count)***	Days: __________<br>99=Don't know
4	What kind of health insurance does the participant has?<br>(Select all that apply)	☐1.Public/civil ☐2.Private<br>☐3.None ☐8.Other, specify:__________________<br>☐9.Don't know

<u>☺Please think about what happened when you had fever and sick with typhoid. If you were sick with another disease, don't tell me about that.</u>

DIRECT COST: Health facility/clinic visits until the day of first interview

B	***☺First, I would like to know how this disease episode affected the participant and his/her household financially.***	
5	Did the participant receive treatment for this illness at the **study health facility** or **any health provider** from the **onset of illness until yesterday** (a day before this interview)?<br>*(If "No" or "Don't know" skip to Section C, Q.8)*	☐1.Yes<br>☐2.No<br>☐9.Don't know
6	Which type of health provider has the participant visited? *(Select all that apply)*<br>*(Refer to * below for response key)*	_____ _____ _____ _____ _____ _____
7	How many times has the participant made a visit **in total**?	_____x _____x _____x _____x _____x _____x
*	***Response key for B6: Option for "Type of health provider" above***<br>**Name codes:** a=Private hospital/ clinic b=Public health facility c=Other health facility d=Pharmacy e=Traditional healer f=Other/self	

Form S.I.#:__________________ **Study label** | | | | | | | | | | |

(To be filled by Data Management)

DETAILS OF HEALTH FACILITY/CLINIC VISITS BEFORE THE DAY OF FIRST INTERVIEW

For the participant who has received treatment for ***this illness*** *from all health care facilities/clinics, please provide following details. Each column numbered 1,2,… represents one care provider visited. Do not report amounts paid by insurance company or program. I want to know how much you or your household had to spend during each visit and additional information.*

C	**Zero visit** is the day participant visits study facility and blood specimen taken (Zero visit for special cases is the visit in which SETA enrollment occurred)	**Visit**				
		**Before Zero Visit**		**During Zero Visit**	**After Zero before interview**	
		1	2	Enrollment Day	1	2
8	Date of visit dd/ mm/ yyyy	____/ ____/ ____	____/ ____/ ____	____/ ____/ ____	____/ ____/ ____	____/ ____/ ____
9	Type of treatment provider *(Refer to # below for response key)*					
10	Was the participant's visit for SETA research purpose?	☐1.Yes ☐2.No ☐9.Don't know	☐1.Yes ☐2.No ☐9.Don't know	☐1.Yes ☐2.No ☐9.Don't know	☐1.Yes ☐2.No ☐9.Don't know	☐1.Yes ☐2.No ☐9.Don't know
11	How long did the participant have to wait after arriving at the health facility before s/he was able to see the provider? (**Ç**: *Record respondents' answer in minutes ONLY. If the waiting time was one and a half hours, record either 90 minutes*)	______ 9999.Don't know	______ 9999.Don't know	______ 9999.Don't know	______ 9999.Don't know	______ 9999.Don't know
12	If you had not visited the health facility, would you have been working – either at home or for wages?	☐1.Yes ☐2.No ☐9.Don't know	☐1.Yes ☐2.No ☐9.Don't know	☐1.Yes ☐2.No ☐9.Don't know	☐1.Yes ☐2.No ☐9.Don't know	☐1.Yes ☐2.No ☐9.Don't know
13	Who paid for the health facility visit? *(Refer to * below for responses key)*					
14	What was the total amount the participant had to spend to receive the treatment at the health facility, not including the cost of food, transportation and overnight stay? *(In local currency unit)*					
15	Did you receive medication? *(If "No" or "Don't know" skip to Q.18)*	☐1.Yes ☐2.No ☐9.Don't know	☐1.Yes ☐2.No ☐9.Don't know	☐1.Yes ☐2.No ☐9.Don't know	☐1.Yes ☐2.No ☐9.Don't know	☐1.Yes ☐2.No ☐9.Don't know
16	Does the out of pocket cost mentioned in Q.14 include medication cost?	☐1.Yes ☐2.No ☐9.Don't know	☐1.Yes ☐2.No ☐9.Don't know	☐1.Yes ☐2.No ☐9.Don't know	☐1.Yes ☐2.No ☐9.Don't know	☐1.Yes ☐2.No ☐9.Don't know

17	If not, how much was the cost of medication paid out of pocket? *(In local currency unit)*					
18	Did you receive any diagnostic test? *(If "No" or "Don't know" skip to Q.21)*	☐1.Yes ☐2.No ☐9.Don't know	☐1.Yes ☐2.No ☐9.Don't know	☐1.Yes ☐2.No ☐9.Don't know	☐1.Yes ☐2.No ☐9.Don't know	☐1.Yes ☐2.No ☐9.Don't know
19	Does the out of pocket cost mentioned in Q.14 include diagnostic costs?	☐1.Yes ☐2.No ☐9.Don't know	☐1.Yes ☐2.No ☐9.Don't know	☐1.Yes ☐2.No ☐9.Don't know	☐1.Yes ☐2.No ☐9.Don't know	☐1.Yes ☐2.No ☐9.Don't know
20	If not, how much was the cost of diagnostics paid out of pocket? *(In local currency unit)*					
21	Did the participant stay overnight at the health facility? *(If "No" or "Don't know" skip to Q.25)*	☐1.Yes ☐2.No ☐9.Don't know	☐1.Yes ☐2.No ☐9.Don't know	☐1.Yes ☐2.No ☐9.Don't know	☐1.Yes ☐2.No ☐9.Don't know	☐1.Yes ☐2.No ☐9.Don't know
22	How many nights did the participant stay (not including today)?					
23	Does the out of pocket cost mentioned in Q.14 include overnight stay cost?	☐1.Yes ☐2.No ☐9.Don't know	☐1.Yes ☐2.No ☐9.Don't know	☐1.Yes ☐2.No ☐9.Don't know	☐1.Yes ☐2.No ☐9.Don't know	☐1.Yes ☐2.No ☐9.Don't know
24	If not, how much was the cost of overnight stay paid out of pocket? *(In local currency unit)*					
25	Did any **friend or family** member **accompany** the participant when s/he sought care? *(If "No" or "Don't know" skip to Q.28)*	☐1.Yes ☐2.No ☐9.Don't know	☐1.Yes ☐2.No ☐9.Don't know	☐1.Yes ☐2.No ☐9.Don't know	☐1.Yes ☐2.No ☐9.Don't know	☐1.Yes ☐2.No ☐9.Don't know
26	How many person(s) accompanied the participant?					
27	Lodging cost for companion (if paid separately). (In local currency unit) *(Skip this question if participant did not stay overnight or did have a companion to the health facility)*					
28	What form of transportation was used to the treatment provider? *(Refer to ^ below for response key)*					
29	**How long** did it take to travel **one-way** to the health facility? (**Ç**: *Record respondents' answer in minutes ONLY. If the waiting time was one and a half hours, record either 90 minutes*)	________ 9999.Don't know	________ 9999.Don't know	________ 9999.Don't know	________ 9999.Don't know	________ 9999.Don't know
30	**One-way** transportation cost for participant & companions to the health facility. *(In local currency unit)*					
31	Food cost for the **participant** during the health facility visit (if paid separately). *(In local currency unit)*					
32	Food cost for **companion** during the health facility visit (if paid separately). *(In local currency unit)*					

Form S.I.#:___________________ **Study label** | | | | | | | | | | |

(To be filled by Data Management)

33	Add-on and miscellaneous costs related to the treatment (eg. toothpaste, soap, phone, herbs, etc.). *(In local currency unit)*					
#	*Response for C9: Option for "Type of treatment provider" above*<br>**Name codes:** a=Private hospital/clinic b=Public health facility c=Other health facility d=Pharmacy e=Traditional healer f=Other/self					

*	*Response option for question C.13 above:* Who paid for this visit? [Total cost]
(1) Paid completely out-of-pocket by you or your household<br>(2) Paid completely by health insurance/employer<br>(3) Paid completely by health care facility<br>(4) Paid partly by insurance and partly out-of-pocket by you or your household<br>(5) Paid partly by health care facility and partly out-of-pocket by you or your household<br>(6) Paid partly by health care facility and partly by insurance<br>(7) Paid partly by health care facility, partly by insurance, and partly out-of-pocket by you or your household<br>(8) Other *(please specify)* ____<br>(9) Don't know	
^	Response option for question C.27 above<br>1.On foot/walking 2.Motorcycle 3.Bicycle 4. Tricycle 5.Animal/cart 6.Car/taxi 7.Bus/truck 8.Ambulance 98.Other, specify:__________________________ 99.Don't know

INDIRECT COSTS: PARTICIPANT

☺: I want to know whether the participant needed any person to take care of him/her (**caretaker**) or whether the participant had to ask for someone else to do his/her work/job(**substitute labourer**) during the participant's illness. Also whether there were days the participant **was completely unable**, or **able to perform some** or **able to perform all** of his/her usual activities **during the illness**. (Ç: Please do not include the day of interview)

D		
34	**In total**, how many days has the participant been sick with typhoid fever from the day your illness started?	_____ days<br>9999=Don't know
35	How many days was the participant **completely unable to perform any** of his/her **usual activities**?	_____ days<br>9999=Don't know
36	How many days was the participant **able to perform some** of his/her **usual activities**?	_____ days<br>9999=Don't know
37	How many days was the participant **able to perform all** of his/her **activities while sick**?	_____ days<br>9999=Don't know
38	What would the participant have been doing mainly if s/he had not been sick?<br><br>*(Check only one main activity)*	☐1.Going to school<br>☐2.Working on own farm or self-employed<br>☐3.House work (eg. Cooking, cleaning, child care giving)<br>☐4.Working for wage<br>☐5.Leisure time<br>☐8.Other, specify____________________

39	How much is the participant normally paid for one day's work?	In local currency: _______________<br>99999999=Don't know
40	Did the participant lose any income because of this illness?<br>*(If "No" or "Don't know" skip to Section E, Q.42)*	☐1.Yes<br>☐ 2.No<br>☐9.Don't know
41	In total, how many days' income did the participant lose because of this illness?	___________ days<br>9999=Don't know

Error Check Box

(Ç: Please check that the days of complete incapacitation, days of debilitation, and normal activity days add up to the total number of days ill.)						
Days of incapacitation (no activity while ill)[35]	+	Days of debilitation (some activity while ill)[36]	+	Days of normal activity (while ill)[37]	+	Total number of days ill (Check that this is equal to number of sick days reported by respondent)[34]
	+		+		=	

Form S.I.#:__________________

(To be filled by Data Management)

Study label ☐☐☐☐☐☐☐☐☐☐

INDIRECT COSTS: SUBSTITUTE LABOR

E		
42	Did anyone perform the participant's usual activities for him/her while s/he was sick with typhoid fever?<br>*(If "No" or "Don't know" skip to Section H, Q.55)*	☐1.Yes<br>☐2.No<br>☐9.Don't know
43	How many people performed the participant's usual activities while s/he was sick with typhoid fever?	__________ Person(s)<br>9999=Don't know

Form S.I.#:___________________ **Study label** | | | | | | | | | | |

(To be filled by Data Management)

**Substitute number F**	44. # The participant's relationship with the person who performed the participant's usual activities while s/he was sick.	45. Is this person an adult, teenager, or a child?<br>1=Adult (17+)<br>2=Teenager (12-16)<br>3=Child (Less than 12)<br>9=DK	46. How many days did s/he perform the participant's usual activities? *(Record number of days and/or number of hours as days/hours)*<br>*Day*□ *Hour*□ 99=DK	47. Did this person perform:<br>1=All of participant's activities<br>2=Some of participant's activities<br>9=DK	48. Was this person paid to perform participant's activities?<br>1= Yes<br>2= No (Mark "0" in Q.49)<br>9 =DK (then mark "99999999" in Q.49)	49. How much was this person paid per day?<br>99999999=DK<br>Enter **local currency** amount
**A**						
**B**						
**C**						
**D**						
**E**						
**F**						

#Response key for Q.44 above: 1. Spouse 2.Mother 3.Father 4.Immediate family member 5.Friend 6.Neighbour 7.Hired labourer 8.Can't say 98.Other, specify

**Substitute number G**	50. Did this person who performed the participant's activities cut back on his or her own usual activities?<br>1= Yes<br>2 = No<br>9 = DK<br>*(If "No" or "Don't know"skip to Section H, Q.55)*	51. How many days did this person who performed the participant's activities cut back on his or her own duties? *(Record number of days and/or number of hours as days/hours)*<br>*Day*□ *Hour*□ 99=DK	52. Was this person who performed the participant's activities able to do:<br>1=Some of his/her own activities<br>2=None of his/her own activities	53. What would this person who performed the participant's activities have been doing if s/he had not been performing the participant's activities while s/he was sick?<br>1 =Going to school<br>2 =Working on a farm<br>3 =Working at home<br>4 =Working for a wage<br>5 =Leisure time<br>8 =Other *(specify)*	54. *(If s/he would have worked for a wage)* How much is this person who performed the participant's activities normally paid for one day's work?<br>99999999=DK<br>Enter **local currency** amount
**A**					
**B**					
**C**					
**D**					
**E**					
**F**					

INDIRECT COSTS: CARETAKING

H		
55	Was the participant so sick that someone had to cut back on his or her own usual activities for one or more days to care for him/her?<br><br>*Caretaking means assuming responsibility for the physical and emotional needs of a minor or health impaired participant because they are incapable of self-care. Caretaking activities can include attending to the participant at home, accompanying the participant during trips to the* [name study facility:_________________________] *or other care providers or running errands for the participant such as picking up medication. (If "No" or "Don't know" skip to Section J, Q.67)*	☐1.Yes<br>☐2.No<br>☐9.Don't know
56	How many people cared for the participant while s/he was sick?	__________ Person(s)<br>9999=Don't know

Caretaker number I-1	57.* The participant's relationship with the person who cared for him/her while s/he was sick.	58. Is this person an adult, teenager, or a Child?<br><br>1=Adult(17+yrs)<br>2=Teenager (12-16 years)<br>3=Child (<12yrs)<br>9=DK	59. How many days did s/he care for the participant?<br><br>*(Record number of days and/or number of hours as days/hours)*<br>*Day*☐ *Hour*☐ 99=DK	60. Was this person paid to care for the participant?<br><br>1= *Yes*<br>2= No *(Mark "0" in Q.61)*<br>9= DK *(Mark 99999999 in Q.61)*	61. How much was this person paid per day to care for the participant?<br><br>DK=99999999<br>Enter **local currency** amount
**A**					
**B**					
**C**					
**D**					
**E**					
**F**					

*Response key for Q.57 above: 1. Spouse 2.Mother 3.Father 4.Immediate family member 5.Friend 6.Neighbour 7.Hired labourer 8.Can't say 98.Other, specify

Caretaker number I-2	62. Did this person who cared for the participant cut back on his or her own usual activities?<br><br>1= *Yes*<br>2 = No<br>9 =DK<br>*(If "No" or "Don't know" skip to Section J, Q.67)*	63. How many days did this person who cared for the participant cut back on his or her own duties?<br><br>*(Record number of days and or number of hours as days/hours)*<br>*Day*☐ *Hour*☐<br>99=DK	64. Was this person who cared for the participant able to do:<br><br>1=Some of his/her own activities<br>2=None of his/her own activities<br>9=DK	65. What would this person who cared for the participant have been doing if he/she had not been caring for the participant while s/he was sick?<br><br>1 = Going to school<br>2 = Working on a farm<br>3 = Working at home<br>4 = Working for a wage<br>5 = Leisure time<br>8 = Other *(specify)*	66. *(If he/she would have worked for a wage)*<br>How much is this person who catered for caretaker's activity paid for one day's work?<br><br>DK=99999999<br>Enter **local currency** amount
**A**					
**B**					

**C**					
**D**					
**E**					
**F**					

KNOWLEDGE AND PREVIOUS EXPERIENCE WITH TYPHOID
Health Risk Perception: *For Participant to Answer*

☺ I want to know what you think about typhoid fever.

**J**		
67	What comes to your mind when we say someone has "severe illness"? Tell me all that you think about this.<br><br>*(Ç: Do not read list. Check all that apply.)*	☐1. Hospitalization<br>☐2. Fainting<br>☐3. Confusion<br>☐4. Wound/bleeding<br>☐5. Repeated laboratory test<br>☐6. Repeated hospital visit<br>☐7. Death<br>☐8. Other, specify: ____________________<br>☐9. Don't know
68	Tell me how someone feels when they have typhoid fever (mention all you know)<br><br>*(Ç: Do not read list. Check all that apply.)*<br><br>*(If "Response 15" is mentioned, skip to Section M, Q.95)*	☐1.Headache ☐2.Exhaustion ☐3.Diarrhea<br>☐4.Rash ☐5.Constipation ☐6.Restlessness<br>☐7.Loss of appetite ☐8.Generalized aching<br>☐9.Cough ☐10.Nosebleeds<br>☐11.Mental confusion ☐12.Malaise ☐13.Nausea<br>☐14. Fever ☐15. Never heard about typhoid<br>☐98. Other, specify: ____________________<br>☐99. Don't know
69	Tell me all the ways that you believe typhoid fever is transmitted. (Mention all you know)<br><br>*(Ç: Do not read list. Check all that apply.)*	☐1. Polluted water/dirty water<br>☐2. Contaminated food<br>☐3. Flies<br>☐4. Lack of safe sanitation/proper disposal of excreta<br>☐5. Sleeping without mosquito net<br>☐6. Contact with patient/carrier<br>☐7. Mosquitoes<br>☐8. Other, specify: ____________________<br>☐9. Don't know
70	If someone is sick with typhoid fever, what do you believe are the best ways to cure this person?<br><br>*(Ç: Do not read list. Check all that apply.)*	☐1. No treatment is necessary<br>☐2. Antibiotics, specify: ____________________<br>☐3. Taking other medicine, specify: _______________<br>☐4. Drinking a lot of clean water<br>☐5. Injections<br>☐6. Changing one's diet<br>☐7. Cleaning house, environment or body<br>☐8. Religious healing<br>☐9. Herbal medicines<br>☐98. Other, specify: ____________________<br>☐99. Don't know

71	What are the best ways to avoid getting typhoid fever?<br><br>*(Ç: Do not read list. Check all that apply.)*	☐1. There is no way to prevent typhoid<br>☐2. Boil water before use/clean water<br>☐3. Control in-house water contamination (safe water storage)<br>☐4. Control in-community water contamination (safe water supplies)<br>☐5. Maintain clean and proper latrines<br>☐6. Maintain adequate waste disposal<br>☐7. Maintain cleanliness in food preparation and handling (e.g. handwashing)<br>☐8. Avoid food from street vendors<br>☐9. Clean the house or environment<br>☐10. Avoid contact with participants or carriers<br>☐11. Eat well-cooked food (including avoiding certain foods)<br>☐12. Get vaccinated<br>☐13.Maintain proper personal hygiene by hand washing<br>☐14. Take preventive medicine, specify: ___________<br>☐98. Other, specify: ____________________________<br>☐99. Don't know
72	Do you think typhoid fever is a severe illness?	☐1.Yes<br>☐2.No<br>☐9.Don't know
73	Who does typhoid affect more seriously?<br><br>*(Ç: Check only one.)*	☐1.Adults<br>☐2.School-age children<br>☐3.Preschool children<br>☐4.Infants<br>☐5.Elderly<br>☐6.Equally serious to all age groups<br>☐7.Not serious in any age group<br>☐8.Other, specify:____________________________<br>☐9.Don't know
**K**		
74	Do you think one gets typhoid fever from your community or from somewhere else?	☐1.In this community<br>☐2.Somewhere else<br>☐8.Other, specify:____________________________<br>☐9.Don't know
75	Has the participant had typhoid fever within the past three years?<br>(If "No" or "Don't know" skip to Q.77)	☐1.Yes<br>☐2.No<br>☐9.Don't know
76	How many times has the participant had typhoid fever within the past three years?	_____ time(s)<br>9999=Don't know
77	Has anyone in your household ever died of typhoid fever?<br>(If "No" or "Don't know" skip to Q.80)	☐1.Yes<br>☐2.No<br>☐9.Don't know
78<br>&<br>79	78. How old were they when they died?<br>A. ______ year(s) 9999=Don't know<br>B. ______ year(s) 9999=Don't know<br>C. ______ year(s) 9999=Don't know	79. What year did they die? (*YYYY*)<br>A. ___________ 9999=Don't know<br>B. ___________ 9999=Don't know<br>C. ___________ 9999=Don't know
80	Has any adult now living in your household had typhoid fever within the past three years and recovered? (If "No" or "Don't know" skip to Q.83)	☐1.Yes<br>☐2.No<br>☐9.Don't know
81	How many adults now living in your household have had typhoid fever within the past three years and recovered?	_____ Adult(s)<br>9999=Don't know

<table>
<tr><td rowspan="5">82</td><td colspan="3">How many times has this adult had typhoid fever within the past three years and recovered?</td></tr>
<tr><td>Relation</td><td>How old?</td><td>How many times?</td></tr>
<tr><td>A.</td><td></td><td></td></tr>
<tr><td>B.</td><td></td><td></td></tr>
<tr><td>C.</td><td></td><td></td></tr>
<tr><td>83</td><td colspan="2">Has any other child, now living in your household had typhoid fever within the past three years and recovered? (If “No” or “Don’t know” skip to Q.86)</td><td>☐1.Yes
☐2.No
☐9.Don’t know</td></tr>
<tr><td>84</td><td colspan="2">How many other children now living in this household have had typhoid fever within the past three years and recovered?</td><td>_____ Child(ren)
9999=Don’t know</td></tr>
<tr><td rowspan="6">85</td><td colspan="3">(Ç: Complete the table by asking the respondent the questions in the top row).
How many times has this child had typhoid fever within the past three years and recovered?</td></tr>
<tr><td>Relation</td><td>How old?</td><td>How many times?</td></tr>
<tr><td>A.</td><td></td><td></td></tr>
<tr><td>B.</td><td></td><td></td></tr>
<tr><td>C.</td><td></td><td></td></tr>
<tr><td colspan="3"></td></tr>
<tr><td rowspan="5">86</td><td colspan="3">☺ Can you tell me the number of people you know outside your family who have ever died of typhoid fever? ☐1.Yes ☐2.No ☐9.Don’t know (If “No” or “Don’t know” skip to Q.87)
Will be glad to know the relation and ages of those people to the participant.</td></tr>
<tr><td>Relation</td><td colspan="2">How old?</td></tr>
<tr><td>A.</td><td colspan="2"></td></tr>
<tr><td>B.</td><td colspan="2"></td></tr>
<tr><td>C.</td><td colspan="2"></td></tr>
</table>

AVERTIVE BEHAVIOUR

**L**		
87	Before the illness started, has any member of this household done anything special or spent any money on anything to specifically avoid getting infected with typhoid fever? *(If “No” or “Don’t know” skip to Section M, Q.95)*	☐1.Yes<br>☐2.No<br>☐9.Don’t know

88	Which practices does your household observe to prevent themselves from typhoid fever?<br><br>*(Ç: Do not read list. Check all that apply.)*	☐1. Boil water before use or use bottled water<br>☐2. Wash raw vegetable thoroughly before use<br>☐3. Maintain cleanliness in food preparation and handling (e.g. handwashing with soap)<br>☐4. Eat well-cooked food (including avoiding certain foods)<br>☐5. Avoid food from street vendors<br>☐6. Seek doctors' advice on prevention measures<br>☐7. Report immediately to the hospital when sick<br>☐8. Vaccination<br>☐9. Control in-community water contamination (safe water supplies)<br>☐10. None of the above<br>☐98.Other, specify: ___________________________<br>☐99=Don't know
89	When do household members of the participant wash hands with soap?<br><br>*(Ç: Do not read list. Check all that apply.)*	☐1.During sickness<br>☐2.Before eating<br>☐3.After eating<br>☐4.Before cooking<br>☐5.After going to toilet<br>☐6.Upon returning home<br>☐7.When washing face<br>☐8.Never<br>☐98. Other, specify: ________________<br>☐99.Don't know
90	What kinds of vegetable washing habits does the participant's household observe?<br><br>*(Ç: Do not read list. Check all that apply.)*<br><br>*(If response is not "9. Do not wash", skip to Q.92)*	☐1.Washing with water only without additive<br>☐2.Washing vegetables with salt water<br>☐3.Washing vegetables with lemon juice<br>☐4.Washing vegetables with vinegar<br>☐5.Washing with mixture of water, vinegar, lemon juice<br>☐6.Just rinsing with water without soap or additive<br>☐7.Soak vegetables in plain water<br>☐8.Soak vegetables in additive mixture<br>☐9.**Do not wash**<br>☐98.Other, specify: ___________________________<br>☐99.Don't know
91	If respondent does not wash vegetables in Q.90, "response 9", ASK why not?	☐1.Feeling lazy<br>☐2. Too time consuming/inconvenient<br>☐3.I need to save water<br>☐4.No need to wash<br>☐5.I trust the shops wash well before selling<br>☐6.Small quantities of germs cannot cause harm<br>☐8.Other, specify: ___________________________<br>☐9.Don't know
92	How many times per week do your household members buy prepared (cooked) food from street vendors?<br>(If "no buying from street vendors" write "**0**" time(s) **BUT** if "Don't know" skip to Q.94)	_____ time(s)<br><br>☐99.Don't know

93	If ZERO times in Q.92, ASK: Why don't the household members buy food from street vendors?	☐1.Too expensive<br>☐2.Too time consuming/inconvenient<br>☐3.Not available<br>☐4.Worried about typhoid<br>☐5.Worried about health risks<br>☐8.Other, specify: ____________________<br>☐9.Don't know
94	How early does one need to see a doctor when feeling sick with typhoid fever? Please select only one option.	☐1.No need to see a doctor<br>☐2.On the first day of symptom start<br>☐3.Three days after symptom start<br>☐4.One week after symptom start<br>☐8.Other, specify:_______________<br>☐9.Don't know

Socio-Economic Information

***Ç:** This information should be collected from the participant/caregiver in case of a child or when participant is unable to communicate.*

Socio-economic Indicators: *For adult participant or caretaker of a minor or any health impaired participant to answer*

M	☺ We are almost done, *let me ask you few questions about yourself*	
95	What is the participant's level of education?<br>*(Ç: Ask proxy person if participant is less than 18 years)*	☐1.Never been to school<br>☐2.Preschool/Kindergarten<br>☐3.1-5 years of school (Primary)<br>☐4.6-9 years of school (JHS)<br>☐5.10-12 years of school (SHS)<br>☐6. Tertiary<br>☐7.Polytechnic /Undergraduate<br>☐8.Other, specify: ____________________<br>☐9.Don't know
96	Is the participant a pupil/student?	☐1.Yes<br>☐2.No, reason: _________________________<br>☐9.Don't know
97	Is the participant currently employed?<br>(If "No" or "Don't know" skip to Q.102)	☐1.Yes<br>☐2.No<br>☐9.Don't know
98	How many months in a year does the participant earn a wage?	__________ months<br>9999=Don't know
99	Does the participant get the same wage every month?<br>*(If "Yes" skip to Q.101)*	☐1.Yes<br>☐2.No<br>☐9.Don't know
100	In which months does the participant earn more?<br>*(Ç: Write all that apply and separate by commas)*<br>*(Refer to ** below for key)*	_________________________<br>99=Don't know
**	Response options for Q.100<br>**Months:** Jan=1, Feb=2, Mar=3, Apr=4, May=5, Jun=6, Jul=7, Aug=8, Sept=9, Oct=10, Nov=11, Dec=12	
101	Is the participant the only earning member of your household?<br>*(If "Yes" skip to Q.103)*	☐1.Yes<br>☐2.No<br>☐9.Don't know
102	Who else earns a wage in your/the participant's household?	1.No. of household members earning: __________<br><br>**In local currency**<br>2.Combined earning per day: Amount: __________

***Ç:** Please ask the respondent these questions in the table below on behalf of his/her **household**. If the respondent says "don't know", record (99) for Q.103. If the respondent refuses to answer, record (97)*

☺: I will like you to now tell me about participants work and how much you/s/he earn(s) per month?

List	103***<br>**Occupation**	104.<br>**Earnings per month**<br>In local currency<br>DK=99999999

Participant		
Parent/caretaker (If different from participant)		
Head of household		

***	*Response option for Q.103 above*<br>**Occupation List**		
[1]<br>[2]<br>[3]<br>[4]<br>[5]<br>[6]<br>[7]<br>[8]<br>[9]	Student/Pupil<br>Retiree<br>Housewife<br>Professional<br>Unskilled office worker<br>Business owner<br>Farmer<br>Unskilled manual worker<br>Skilled manual worker	[10]<br>[11]<br>[12]<br>[13]<br>[14]<br>[15]<br>[97]<br>[98]<br>[99]	Street seller<br>Driver<br>Public servant<br>Petty trader<br>Fisherman<br>Service worker (eg. servant, cook, hotel or restaurant worker)<br>Refused to answer<br>Other, specify: ______________________________<br>Don't know

105	How many people live with the participant in the same household?<br>*(i.e. Number of all persons living in a house or a cluster of houses who share a common cooking pot)*	__________ person(s).<br>9999=Don't know
106	What is the main source of drinking water for members of your household?<br><br>*(If "Response 1, 2 or 9" is mentioned, go to "Q.107")*	**PIPED WATER**<br>☐1.Piped into dwelling/household connection<br>☐2.Piped plot/yard connection<br>☐3.Public tap/standpipe<br>☐4.**TUBEWELL/BOREHOLE**<br>**DUG WELL**<br>☐5.Protected well<br>☐6.Unprotected well<br>**WATER FROM SPRING**<br>☐7.Protected spring<br>☐8.Unprotected spring<br>☐9.**RAINWATER**<br>☐10.**TANKER TRUCK**<br>☐11.**CART WITH SMALL TANK/DRUM**<br>☐12.**SURFACE WATER(RIVER/DAM/LAKE/POND/ STREAM/CANAL/IRRIGATION CHANNEL)**<br><br>☐13.**BOTTLED WATER**<br>☐98.Other, specify: ______________________________

107	What kind of toilet facility do members of your household usually use?<br><br>*(If "Response 12" is mentioned, skip to "Q.110")*	**FLASH OR POUR FLUSH TOILET**<br>☐1.Flush to piped sewer system<br>☐2.Flush to septic tank<br>☐3.Flush to pit latrine<br>☐4.Flush to somewhere else<br>☐5.Flush, don't know where<br>**PIT LATRINE**<br>☐6.Ventilated improved pit latrine<br>☐7.Pit latrine with slab<br>☐8.Pit latrine without slab/open pit<br>☐9.COMPOSING TOILET<br>☐10.BUCKET TOILET<br>☐11.HANGING TOILET/HANGING LATRINE<br>☐12.NO FACILITY/BUSH/FIELD<br>☐98.Other, specify:__________________________
108	Do you share this toilet facility with other households?<br>*(If "No" skip to Q.110)*	☐1.Yes<br>☐2.No
109	How many households use this toilet facility?	☐1.Number of households if less than 10: ☐<br>☐2.10 or more households<br>☐9.Don't know
110	*(Ç: Read out each item and select the answer given. Multiple responses possible. Do not leave any item blank. If none, select 'No'.)*<br><br>Does your household have?<br><br>Electricity?<br>A Radio?<br>A television?<br>A mobile telephone?<br>A non-mobile telephone?<br>A refrigerator?	(see below)

☐1.Electricity	☐1.Yes	☐2.No
☐2.Radio	☐1.Yes	☐2.No
☐3.Television	☐1.Yes	☐2.No
☐4.Mobile telephone	☐1.Yes	☐2.No
☐5.Non-mobile telephone	☐1.Yes	☐2.No
☐6.Refrigerator	☐1.Yes	☐2.No
☐7.Chair	☐1.Yes	☐2.No
☐8.Bed	☐1.Yes	☐2.No
☐9.Sofa	☐1.Yes	☐2.No
☐10.Cupboard	☐1.Yes	☐2.No
☐11.Table	☐1.Yes	☐2.No
☐12.Clock	☐1.Yes	☐2.No
☐13.Grain grinder	☐1.Yes	☐2.No
☐14.Casstette/CD player	☐1.Yes	☐2.No
☐15.Fan	☐1.Yes	☐2.No

111	What type of fuel does your household mainly use for cooking?<br><br>*(Ç: If the household uses more than one fuel for cooking, find out the fuel used most often. If any fuel other than the precoded ones is reported as being the main fuel used for cooking, select '98' and specify the type of fuel in the space provided)*	☐1.Electricity<br>☐2.LPG<br>☐3.Natural gas<br>☐4.Biogas<br>☐5.Kerosine<br>☐6.Coal, Lignite<br>☐7.Charcoal<br>☐8.Wood<br>☐9.Straw/Shrubs/Grass<br>☐10.Agricultural crop<br>☐11.Animal dung<br>☐12.No food cooked in household<br>☐98.Other, specify:__________________________
112	Main material of the floor.<br><br>*(Ç: Record observation; do not ask but check for yourself. If there is more than one kind of flooring material, record the main type of material (the material that covers the largest amount of floor space).)*	**NATURAL FLOOR**<br>☐1.Earth/Sand<br>☐2.Dung<br>**RUDIMENTARY FLOOR**<br>☐3.Wood planks<br>☐4.Palm/Bamboo<br>**FINISHED FLOOR**<br>☐5.Parquet or polished wood<br>☐6.Vinyl or asphalt strips<br>☐7.Ceramic tiles<br>☐8.Cement<br>☐9.Carpet<br>☐98.Other, specify:__________________________
113	Main material of the roof.<br><br>*(Ç: Record observation, in case of doubt asks the head of household. In case of different kinds of roofing material, record the main type of material that covers the largest amount of roof space.)*	**NATURAL ROOFING**<br>☐1.No roof<br>☐2.Thatch/Palm leaf<br>☐3.Sod<br>**RUDIMENTARY ROOFING**<br>☐4.Rustic mat<br>☐5.Palm/Bamboo<br>☐6.Wood planks<br>☐7.Cardboard<br>**FINISHED ROOFING**<br>☐8.Metal<br>☐9.Wood<br>☐10.Calamine/Cement fiber<br>☐11.Ceramic tiles<br>☐12.Cement<br>☐13.Roofing shingles<br>☐98.Other, specify:__________________________

114	Main material of the exterior walls.<br><br>*(Ç: Record observation, in case of doubt asks the head of household. In case of different kinds of exterior wall material, record the main type of material that covers the largest amount of wall space.)*	**NATURAL WALLS**<br>☐1.No walls<br>☐2.Cane/Palm/Trunks<br>☐3.Dirt<br>**RUDIMENTARY WALLS**<br>☐4.Bamboo with mud<br>☐5.Stone with mud<br>☐6.Uncovered adobe<br>☐7.Plywood<br>☐8.Cardboard<br>☐9.Reused wood<br>**FINISHED WALLS**<br>☐10.Cement<br>☐11.Stone with lime/Cement<br>☐12.Bricks<br>☐13.Cement blocks<br>☐14.Covered adobe<br>☐15.Wood planks/shingles<br>☐98.Other, specify:_______________________
115	How many rooms in this household are used for sleeping?	Room(s): _____
116	Does any member of this household own<br><br>A watch?<br>A bicycle?<br>A motorcycle or motor scooter?<br>An animal-drawn cart?<br>A car or truck?<br>A boat with a motor?<br><br>(*Ç:* Do not record children's bicycle as it is considered primarily as a toy.)	☐1.Watch ☐1.Yes ☐2.No<br>☐2.Bicycle ☐1.Yes ☐2.No<br>☐3.Motorcycle/Scooter ☐1.Yes ☐2.No<br>☐4.Animal-drawn cart ☐1.Yes ☐2.No<br>☐5.Car/Truck ☐1.Yes ☐2.No<br>☐6.Boat with motor ☐1.Yes ☐2.No
117	Does any member of this household own any agricultural land? *(If "No", skip to "Q.119")*	☐1.Yes<br>☐2.No
118	How many hectares of agricultural land do members of this household own?<br><br>*(If 95 or more, select "Response 2".)*	☐1. Hectares: ☐☐.☐<br>☐2. 95 or more hectares<br>☐9.Don't know
119<br>~~124~~	Does this household own any livestock, herds, other farm animals, or poultry?<br>*(If "No" or, skip to "Q.121")*	☐1.Yes<br>☐2.No

120	*(Ç: Read out each item and select the answer given. Multiple responses possible. Do not leave any item blank)*<br><br>How many of the following animals do this household own?<br><br>Cattle?<br>Milk cows or bulls?<br>Horses, donkeys, or mules?<br>Goats?<br>Sheep?<br>Chickens?<br><br>If none, enter '00'.<br>If 95 or more, enter '95'.<br>If unknown, enter '99'.	☐1.Cattle . . . .<br>☐2.Cows/Bulls . . . .<br>☐3.Horses/Donkeys/Mules . . . . ….<br>☐4.Goats . . . .<br>☐5.Sheep . . . .<br>☐6.Chickens . . . .<br>☐7.Pigs…………... . . . .… .<br>☐8.Ducks…………………………….<br>☐98.Other, specify:……………….....
121	Does any member of this household have a bank account?	☐1.Yes<br>☐2.No
122	What is the participant's source of drinking water outside home?	☐1.Kiosk/stall water seller<br>☐2.Water from street vendors<br>☐3.Bottled/Sachet water from local residents<br>☐4.Bottled/Sachet water from professional firms<br>☐5.Water from unprotected containers/barrels<br>☐6.Water from protected spring<br>☐7.Water from unprotected spring<br>☐8.Other, specify: ________________________________<br>☐9.Don't know
123	Does the participant have access to **a toilet outside home**?<br>(If "No" or "Don't know" skip to Q.125)	☐1.Yes<br>☐2.No<br>☐9.Don't know
124	Type of **toilet outside home**	**PUBLIC FLASH OR POUR FLUSH TOILET**<br>☐1.Public flush to pipe sewer system<br><br>**PUBLIC PIT LATRINE**<br>☐2.Ventilated improved pit latrine<br>☐3.Pit latrine with slab<br>☐4.Pit latrine without slab/open pit<br><br>☐5.PUBLIC HANGING TOILET/HANGING LATRINE<br>☐6.NO FACILITY/BUSH/FIELD<br><br>☐8.Other, specify:__________________________<br>☐9.Don't know

125	Which toilet does the participant often use?	☐1.Home toilet<br>☐2.Outside toilet<br>☐3.Do not use<br>☐8.Other, specify:___________________________<br>☐9.Don't know
126	Who is the owner of the house the participant lives in?	☐1.Self ☐2.Parents ☐3.Family<br>☐4.Provided by employer ☐5.Rented<br>☐8.Other, specify:___________________________<br>☐9.Don't know
127	How much does the participant's household spend on average during the month?	**In local currency:**<br>________________<br>99999999=Don't know<br>98888888=No response
128	How much was the participant's household's own electricity related cost last month? Or, if the bill is being shared, how much was the participant's share?	**In local currency**<br>Household's own share of electricity bill: ___________<br>66666666=We pay no monthly charge for electricity? It's included in the rent<br>99999999=Don't know/Not sure<br>88888888=No response
129	How much was the participant's household's telephone (land line and mobile) related cost last month?	**In local currency**<br>Household's own share of phone bill: ___________<br>99999999=Don't know<br>~~9~~88888888=No response

**N**		
130	Is the participant still sick with fever/typhoid?	☐1.Yes ☐2.No ☐3.Can't say<br>*If "**YES**" or "**Can't say**", schedule next interview after finishing to administer all applicable questionnaires on this interview.*<br>*If "**No**", end COI follow-up after finishing to administer all applicable questionnaires on this interview.*

**O**	**Further Questions**	
131	Is the participant also being treated for any illness in addition to typhoid fever from the onset of current febrile illness? (Eg. Any injuries, accident, diabetes, etc.)<br>*(If "Yes" use Form F4: Cost of Other Illness Initial Interview)*<br>*(If "No" or "Don't know" use Form F6.1: LT-SES Survey Form for cases' Initial Interview)*	☐1.Yes<br>☐2.No<br>☐9.Don't know

Annex 2: SETA COI Follow-up Interview Tool

Form F3: COI Follow-Up (FU) Interview

(Day ___ to ___: ___☐week/☐month post enrollment by Illness continuation)

F3 for *S*. Typhi or Special or *S*. Paratyphi or iNTS positive and clinically diagnosed laboratory negative cases

Note to Interviewer
*If you see **"Ç"**, that is a **note for you** to read by yourself. If you see **"☺"** you should **read out loud** for the respondent or the caretaker/Next of kin.*

Site/**C**ountry: ______________________________/______________________________

Follow-up Schedule: ☐Day 3-7 (for special cases only)
☐Day 12-14 ☐Day 28-30 ☐Day 90 ☐Day 180 ☐Day 270 ☐Day 360

Interviewer's Name:_________________________ **D**ate of Interview(dd/mm/yyyy): _____/_____/__________
Laboratory diagnosis: ☐*S.* Typhi ☐Special Case ☐*S.* Paratyphi ☐iNTS ☐Clinical enteric fever

Place of interview: ☐Health facility / ☐Home / ☐Other, specify: ________________________
If at health facility, indicate point of care: ☐Out-Patient / ☐In-Patient;
If In-Patient, name of admission ward: ______________________________
Date of Admission: _____/_____/__________
Date of Discharge (dd/mm/yyyy): _____/_____/__________ [**Ç:** Look this up in the study facility record]

**Ç: Check whether participant completed the diary card.**	
**L**ogbook (Diary card) completed?	☐1.Yes ☐2.No

Cost-of-Illness Questionnaire: ___- ___ day Follow-Up

Ç: Is the participant the right person interviewed ____ ☐weeks/☐months (____ to ____ days) ago.		
0	Correct?	☐1.Yes ☐2.No ☐9.Don't know
***Ç:** If "**No**" or "**Don't know**", identify the right candidate to continue the interview.*		

***Ç:** Use this instrument to interview an adult participant/caretaker/Next of kin(in case of a minor or if health-impaired) that is familiar with the participant and his/her treatment over the last few days/weeks/months.*
☺ I am going to ask you about the participant's illness and the costs that the participant/their household incurred for management of the Illness until yesterday.

☺ Please refer to the logbook (diary card) recorded when you were/the participant was sick.
***Ç:** If the respondent has their logbook(diary card) before them. ☺ Please check your logbook if it will help you remember.*

☺***Please think about what happened when you had fever and sick with typhoid. If you were sick with another disease, don't tell me about that.***

DIRECT COSTS: Health facility/clinic visits until yesterday before this interview

**A**	☺ ***First, I would like to know how this disease episode affected the participant and his/her household financially. Ç: Please, cross check with the participant's diary card to see if answers are comparable.***	
1	Did the participant receive treatment for this illness at the study health facility or **any health provider** from the **day of our last interview (including the day of that interview) until yesterday**? *(If "No" or "Don't know" skip to Section B, Q.4)*	☐1.Yes ☐2.No ☐9.Don't know
2	Which type of health provider has the participant visited? *(Select all that apply)* *(Refer to ++ below for response key)*	_____ _____ _____ _____ _____ _____
3	How many times has the participant made a visit **in total**?	_____x _____x _____x _____x _____x _____x
++	***Response for A2: Option for "Type of health provider" above*** **Name codes:** a=Private hospital/ clinic b=Public health facility c=Other health facility d=Pharmacy e=Traditional healer f=Other/self	

DETAILS OF HEALTH FACILITY/CLINIC VISITS FROM THE DAY OF LAST INTERVIEW TO A DAY BEFORE THIS INTERVIEW (YESTERDAY)

*For the participant who has received treatment for **this illness** from all health care facilities/clinics, please provide following details. Each column numbered 1,2,3… represents one care provider visited. Do not report payments made by insurance company or program. I want to know how much you or your household had to spend during each visit and other things.*

**B**	**All health facility visits from the day of last interview till yesterday (Day ___ - ___)**	**Visit**				
		**Day of last interview to day before this interview**				
		1	2	3	4	5
4	Date of visit<br>dd/<br>mm/<br>yyyy	_____/<br>_____/<br>_____	____/<br>____/<br>____	____/<br>____/<br>____	____/<br>____/<br>____	____/<br>____/<br>____
5	Type of treatment provider<br>*(Refer to # below for response key)*					
6	Was the participant's visit for SETA research purpose?	☐1.Yes<br>☐2.No<br>☐9.Don't know	☐1.Yes<br>☐2.No<br>☐9.Don't know	☐1.Yes<br>☐2.No<br>☐9.Don't know	☐1.Yes<br>☐2.No<br>☐9.Don't know	☐1.Yes<br>☐2.No<br>☐9.Don't know
7	How long did the participant have to wait after arriving at the health facility before s/he was able to see the provider?<br><br>(Ç: *Record respondents' answer in minutes ONLY. If the waiting time was one and a half hours, record either 90 minutes*)	_______<br>9999.Don't know	_______<br>9999.Don't know	_______<br>9999.Don't know	_______<br>9999.Don't know	_______<br>9999.Don't know
8	If you had not visited the health facility, would you have been working – either at home or for wages?	☐1.Yes<br>☐2.No<br>☐9.Don't know	☐1.Yes<br>☐2.No<br>☐9.Don't know	☐1.Yes<br>☐2.No<br>☐9.Don't know	☐1.Yes<br>☐2.No<br>☐9.Don't know	☐1.Yes<br>☐2.No<br>☐9.Don't know
9	Who paid for the health facility visit?<br>*(Refer to * below for response key)*					
10	What was the total amount the participant had to spend to receive the treatment at the health facility, not including the cost of food, transportation and overnight stay? *(In local currency unit)*					
11	Did you receive medication?<br>*(If "No" or "Don't know" skip to Q.14)*	☐1.Yes<br>☐2.No<br>☐9.Don't know	☐1.Yes<br>☐2.No<br>☐9.Don't know	☐1.Yes<br>☐2.No<br>☐9.Don't know	☐1.Yes<br>☐2.No<br>☐9.Don't know	☐1.Yes<br>☐2.No<br>☐9.Don't know
12	Does the out of pocket cost mentioned in Q.10 include medication cost?	☐1.Yes<br>☐2.No<br>☐9.Don't know	☐1.Yes<br>☐2.No<br>☐9.Don't know	☐1.Yes<br>☐2.No<br>☐9.Don't know	☐1.Yes<br>☐2.No<br>☐9.Don't know	☐1.Yes<br>☐2.No<br>☐9.Don't know
13	If not, how much was the cost of medication paid out of pocket? *(In local currency unit)*					

14	Did you receive any diagnostic test?<br>*(If "No" or "Don't know" skip to Q.17)*	☐1.Yes<br>☐2.No<br>☐9.Don't know	☐1.Yes<br>☐2.No<br>☐9.Don't know	☐1.Yes<br>☐2.No<br>☐9.Don't know	☐1.Yes<br>☐2.No<br>☐9.Don't know	☐1.Yes<br>☐2.No<br>☐9.Don't know
15	Does the out of pocket cost mentioned in Q.10 include diagnostic costs?	☐1.Yes<br>☐2.No<br>☐9.Don't know	☐1.Yes<br>☐2.No<br>☐9.Don't know	☐1.Yes<br>☐2.No<br>☐9.Don't know	☐1.Yes<br>☐2.No<br>☐9.Don't know	☐1.Yes<br>☐2.No<br>☐9.Don't know
16	If not, how much was the cost of diagnostics paid out of pocket? *(In local currency unit)*					
17	Did the participant stay overnight at the health facility?<br>*(If "No" or "Don't know" skip to Q.21)*	☐1.Yes<br>☐2.No<br>☐9.Don't know	☐1.Yes<br>☐2.No<br>☐9.Don't know	☐1.Yes<br>☐2.No<br>☐9.Don't know	☐1.Yes<br>☐2.No<br>☐9.Don't know	☐1.Yes<br>☐2.No<br>☐9.Don't know
18	How many nights did the participant stay?					
19	Does the out of pocket cost mentioned in Q.10 include overnight stay cost?	☐1.Yes<br>☐2.No<br>☐9.Don't know	☐1.Yes<br>☐2.No<br>☐9.Don't know	☐1.Yes<br>☐2.No<br>☐9.Don't know	☐1.Yes<br>☐2.No<br>☐9.Don't know	☐1.Yes<br>☐2.No<br>☐9.Don't know
20	If not, how much was the cost of overnight stay paid out of pocket? *(In local currency unit)*					
21	Did any **friend or family** member **accompany** the participant when s/he sought care?<br>*(If "No" or "Don't know" skip to Q.24)*	☐1.Yes<br>☐2.No<br>☐9.Don't know	☐1.Yes<br>☐2.No<br>☐9.Don't know	☐1.Yes<br>☐2.No<br>☐9.Don't know	☐1.Yes<br>☐2.No<br>☐9.Don't know	☐1.Yes<br>☐2.No<br>☐9.Don't know
22	How many person(s) accompanied the participant?					
23	Lodging cost for companion (if paid separately).<br>(In local currency unit)<br>*(Skip this question, if participant did not stay overnight or did have a companion to the health facility)*					
24	What form of transportation was used to the treatment provider?<br>*(Refer to ^ below for response key)*					
25	**How long** did it take to travel **one-way** to the health facility?<br>(Ç: *Record respondents' answer in minutes ONLY. If the waiting time was one and a half hours, record either 90 minutes*)	_______<br>9999.Don't know	_______<br>9999.Don't know	_______<br>9999.Don't know	_______<br>9999.Don't know	_______<br>9999.Don't know
26	**One-way** transportation cost for participant & companions to the health facility.<br>*(In local currency unit)*					
27	Food cost for **participant** during the health facility visit (if paid separately). *(In local currency unit)*					
28	Food cost for **companion** during the health facility visit (if paid separately). *(In local currency unit)*					
29	Add-on and miscellaneous cost related to the treatment (eg. toothpaste, soap, phone, herbs, etc.). *(In local currency unit)*					

#	*Response for question B5: Option for "Type of treatment provider" above*<br>**Name codes:** a=Private hospital/ clinic b=Public health facility c=Other health facility d=Pharmacy<br>e=Traditional healer f=Other/self

*	*Response option for question B.9 above:* Who paid for this visit? [Total cost]
(1) Paid completely out-of-pocket by you or your household<br>(2) Paid completely by health insurance/employer<br>(3) Paid completely by health care facility<br>(4) Paid partly by insurance and partly out-of-pocket by you or your household<br>(5) Paid partly by health care facility and partly out-of-pocket by you or your household<br>(6) Paid partly by health care facility and partly by insurance<br>(7) Paid partly by health care facility, partly by insurance, and partly out-of-pocket by you or your household<br>(8) Other *(please specify)* ____<br>(9) Don't know	
^	Response option for question B.23 above<br>1.On foot/walking 2.Motorcycle 3.Bicycle 4. Tricycle 5.Animal/cart 6.Car/taxi 7.Bus/truck<br>8.Ambulance 98.Other, specify:___________________________ 99.Don't know

INDIRECT COSTS: PARTICIPANT

☺: I want to know whether the participant needed any person to take care of him/her (**caretaker**) or whether the participant had to ask for someone else to do his/her work/job(**substitute labourer**) during the participant's illness. Also whether there were days the participant **was completely unable**, or **able to perform some** or **able to perform all** of his/her daily routine **during the illness**. (Ç: Please do not include the day of interview)

C	*Ç: Please, cross check with the participant's diary card to see if answers are comparable.*	
30	**In total**, how many days has the participant been sick with typhoid fever since the day of last interview?	_____ days<br>9999=Don't know
31	How many days was the participant **completely unable to perform any** of his/her **usual activities**?	_____ days<br>9999=Don't know
32	How many days was the participant **able to perform some** of his/her **usual activities**?	_____ days<br>9999=Don't know
33	How many days was the participant **able to perform all** of his/her **activities while sick**?	_____ days<br>9999=Don't know
34	What would the participant have been doing if s/he had not been sick?<br><br>*(Check only one main activity)*	☐1.Going to school<br>☐2.Working on own farm or self-employed<br>☐3.House work (eg. Cooking, cleaning, child care giving)<br>☐4.Working for wage<br>☐5.Leisure time<br>☐8.Other, specify_____________________
35	How much is the participant normally paid for one day's work?	In local currency: _______________<br>99999999=Don't know

36	Did the participant lose any income because of this illness?<br>*(If "No" or "Don't know" skip to Section D, Q.38)*	☐1.Yes<br>☐2.No<br>☐9.Don't know
37	In total, how many days' income did the participant lose because of this illness?	______ days<br>9999=Don't know

Error Check Box

(Ç: Please check that the days of complete incapacitation, days of debilitation, and normal activity days add up to the total number of days ill.)						
Days of incapacitation (no activity while ill)[31]	+	Days of debilitation (some activity while ill)[32]	+	Days of normal activity (while ill)[33]	+	Total number of days ill (Check that this is equal to number of sick days reported by respondent)[30]
	+		+		=	

INDIRECT COSTS: SUBSTITUTE LABOR

**D**		
38	Did anyone perform the participant's usual activities for you or him/her while you, s/he was sick with typhoid fever?<br>*(If "No" or "Don't know" skip to Section G, Q.51)*	☐1.Yes<br>☐2.No<br>☐9.Don't know
39	How many people performed your or the participant's usual activities while you or s/he was sick with typhoid fever?	_____ Person(s)<br>9999=Don't know

Substitute number E	40. # The participant's relationship with the person who performed the participant's usual activities while s/he was sick.	41. Is this person an adult, teenager, or a child?<br>1=Adult (17+)<br>2=Teenager (12-16)<br>3=Child (Less than 12)<br>9=DK	42. How many days did s/he perform the participant's usual activities?<br>*(Record number of days and/or number of hours as days/hours)*<br>*Day*☐ *Hour*☐<br>9999=DK	43. Did this person perform:<br>1=All of participant's activities<br>2=Some of participant's activities<br>9=DK	44. Was this person paid to perform activities?<br>1= Yes<br>2= No (Mark "0" in Q.45)<br>9=DK (then mark "99999999" in Q.45)	45. How much was this person paid per day?<br>DK=99999999<br>Enter **local currency** amount
**A**						
**B**						
**C**						
**D**						
**E**						
**F**						

#Response key for Q.40 above: 1. Spouse 2.Mother 3.Father 4.Immediate family member 5.Friend 6.Neighbour 7.Hired labourer 8.Can't say 98.Other, specify

Substitute number F	46. Did this person who performed the participant's activities cut back on his or her own usual activities?<br>1= Yes<br>2 = No<br>9 = DK<br>*(If "No" or "Don't know"skip to G.51)*	47. How many days did this person who performed the participant's activities cut back on his or her own duties?<br>*(Record number of days and/or number of hours as days/hours)*<br>*Day*☐ *Hour*☐ 9999=DK	48. Was this person who performed the participant's activities able to do:<br>1=Some of his/her own activities<br>2=None of his/her own activities	49. What would this person who performed the participant's activities have been doing if s/he had not been performing the participant's activities while s/he was sick?<br>1=Going to school<br>2 = Working on a farm<br>3 =Working at home<br>4 =Working for a wage<br>5 =Leisure time<br>8=Other, *specify*	50. *(If s/he would have worked for a wage)* How much is this person who performed the participant's activities normally paid for one day's work?<br>DK=99999999<br>Enter **local currency** amount
**A**					
**B**					
**C**					
**D**					
**E**					
**F**					

INDIRECT COSTS: CARETAKING

G		
51	Was the participant so sick that someone had to cut back on his or her own usual activities for one or more days just to care for him/her?<br><br>*Caretaking means assuming responsibility for the physical and emotional needs of a minor or health impaired participant because they are incapable of self-care. Caretaking activities can include attending to the participant at home, accompanying the participant during trips to the* [name study facility:___________________________] *or other care providers or running errands for the participant such as picking up medication.*<br>*(If "No" or "Don't know" skip to Section I, Q.63)*	☐ 1.Yes<br>☐2.No<br>☐9.Don't know
52	How many people cared for the participant while s/he was sick?	___________ Person(s)<br>9999=Don't know

Caretaker number H-1	53.* The participant's relationship with the person who cared for the participant while s/he was sick.	54. Is this person an adult, teenager, or a child?<br><br>1=Adult(17+yrs)<br>2=Teenager (12-16 years)<br>3=Child (<12yrs)<br>9=DK	55. How many days did he/she care for the participant?<br><br>*(Record number of days and/or number of hours as days/hours)*<br>*Day*☐ *Hour*☐ 99=DK	56. Was this person paid to care for the participant?<br><br>1= *Yes*<br>2= No *(Mark "0" in Q.57)*<br>9= DK *(Mark 99999999 in Q.57)*	57. How much was this person paid per day to care for the participant?<br><br>DK=99999999<br>Enter **local currency** amount
**A**					
**B**					
**C**					
**D**					
**E**					
**F**					

*Response key for Q.53 above: 1. Spouse 2.Mother 3.Father 4.Immediate family member 5.Friend 6.Neighbour 7.Hired labourer 8.Can't say 98.Other, specify

Caretaker number H-2	58. Did this person who cared for the participant cut back on his or her own usual activities?<br>1= *Yes*<br>2 = No<br>9=DK<br>*(If "No" or "Don't know" skip to Section I, Q.63)*	59. How many days did this person who cared for the participant cut back on his or her own duties?<br>*(Record number of days and/or number of hours as days/hours)*<br>*Day*☐ *Hour*☐<br>99=DK	60. Was this person who cared for the participant able to do:<br>1=Some of his/her own activities<br>2=None of his/her own activities<br>9=DK	61. What would this person who cared for the participant have been doing if he/she had not been caring for the participant while s/he was sick?<br>1= Going to school<br>2 = Working on a farm<br>3 = Working at home<br>4 = Working for a wage<br>5 = Leisure time<br>8=Other, *specify*	62. *(If he/she would have worked for a wage)*<br>How much is this person who cared for the participant normally paid for one day's work?<br>DK=99999999<br>Enter **local currency** amount
**A**					
**B**					
**C**					
**D**					
**E**					
**F**					

**I**	**FRICTION COST**	
63	You were sick for ___ to ___ days now, and had many unfulfilled tasks. Do you have to work extra to compensate for the missed tasks due to the illness?	☐1.Yes<br>☐2.No.
64	If YES, by working extra do you think you will complete all the tasks which you have missed?	☐1.Yes<br>☐2.No.

**J**		
65	Is the participant still sick with fever/typhoid?	☐1.Yes ☐2.No ☐3.Can't say<br>*If "**YES**" or "**Can't say**", schedule next interview after finishing to administer all applicable questionnaires on this interview.*<br>*If "**No**", end COI follow-up after finishing to administer all applicable questionnaires on this interview.*

**K**	**FURTHER QUESTIONS**	
66	Is the participant also being treated for any illness in addition to typhoid fever since the last interview? (Eg. Any injuries, accident, diabetes, etc.)<br>*(If "Yes" use Form F5: COOI Follow-Up Interview)*<br>*(If "No" or "Don't know" use Form F6.2: LT-SES Survey Form Case and Control's Follow-Up for S.Typhi/Special cases and Control participant follow-up interview)*	☐1.Yes<br>☐2.No<br>☐9.Don't know

Annex 3: SETA COOI Initial Interview Tool

Form F4: Cost-Of-Other-Illness (COOI) Initial Interview

(Day 3 to 7: 1 Week Post Enrollment)

F4 for any illness of all cases and for neighbourhood controls

Note to Interviewer
*If you see "**Ç**", that is a **note for you** to be read by yourself. If you see "☺" you should **read out loud** for the respondent or the caretaker/Next of kin.*

Site/Country: ______________________________/______________________________

Follow-up Schedule: ☐Day 0 (for special cases only) ☐Day 3-7

Interviewer's Name:_________________________ Date of Interview (dd/mm/yyyy): _____/_____/__________

Laboratory diagnosis: ☐*S.* Typhi ☐Special cases ☐*S.* Paratyphi ☐iNTS

☐Clinical enteric fever ☐Control Other illness of the participant (if any): ____________________

Screening Question

**If a "Case Participant"**<br>☺*: Before the date of your health facility visit, did you have any illness apart from typhoid?*
**If a "Control Participant"**<br>☺: *Before the date of study facility visit for the index case (lab positive S. Typhi/Special case) (dd/mm/yyyy): _____/_____/__________), did you have other illness?*

Place of interview: ☐Health facility / ☐Home / ☐Other, specify: _________________________

If at health facility, indicate point of care: ☐Out-Patient / ☐In-Patient;

If In-Patient, name of admission ward: _________________________

Date of Admission (dd/mm/yyyy): _____/_____/__________

Date of Discharge (dd/mm/yyyy): _____/_____/__________ [**Ç**: Look this up in the study facility record]

COOI Questionnaire: Day 3-7 (Upon availability of diagnosis results)

***Ç**: Is the participant the right person interviewed 3-7 days ago?*		
0	Correct?	☐1.Yes ☐2.No ☐9.Don't know
***Ç**: If "**No**" or "**Don't know**", identify the right candidate to continue the interview.*		

***Ç**: Use this instrument to interview an adult participant/caretaker/Next of kin(in case of a minor or if health-impaired) that is familiar with the participant and his/her treatment over the last few days.*
☺: Now I am going to ask you about the participant's other illness and the costs that the participant/their household incurred for the management of that Illness until yesterday.

☺***Please think about what happened when you were sick with the other illness. Please tell me only about this condition.***

DIRECT COSTS: Health facility/clinic visits until the day of first interview

**A**	☺ ***First, I would like to know how this disease episode affected the participant and his/her household financially.***	
1	Did the participant receive treatment for this illness at the **study health facility or any health provider** from the **onset of illness until yesterday** (a day before this interview)? *(If "No" or "Don't know" skip to Section B, Q.4)*	☐1.Yes<br>☐2.No<br>☐9.Don't know
2	Which types of health provider have you/the participant visited? *(Select all that apply)*<br>*(Refer to ++ below for response key)*	_____ _____ _____ _____ _____ _____
3	How many times have you/the participant made a visit **in total**?	_____x _____x _____x _____x _____x _____x
++	***Response key for A2: Option for "Type of health provider" above***<br>**Name codes:** a=Private hospital/ clinic b=Public health facility c=Other health facility d=Pharmacy e=Traditional healer f=Other/self	

DETAILS OF HEALTH FACILITY/CLINIC VISITS BEFORE THE DAY OF FIRST INTERVIEW FOR OTHER ILLNESS(ES)

*For the participant who has received treatment for **other illness** from all health care facilities/clinics, please provide following details. Each column numbered 1,2,… represents one care provider visited. Do not report amounts paid by insurance company or program. I want to know how much you or your household had to spend during each visit and other things.*

B	**Zero visit** is the day participant visits study family and blood specimen taken	**Visit**				
		**Before Zero Visit**		**During Zero Visit**	**After Zero before interview**	
		1	2	Enrollment Day	1	2
4	Date of visit<br>dd/<br>mm/<br>yyyy	____/<br>____/<br>____	____/<br>____/<br>____	____/<br>____/<br>____	____/<br>____/<br>____	____/<br>____/<br>____
5	Type of treatment provider<br>*(Refer to # below for response key)*					
6	Was the participant's visit for SETA research purpose?	☐1.Yes<br>☐2.No<br>☐9.Don't know	☐1.Yes<br>☐2.No<br>☐9.Don't know	☐1.Yes<br>☐2.No<br>☐9.Don't know	☐1.Yes<br>☐2.No<br>☐9.Don't know	☐1.Yes<br>☐2.No<br>☐9.Don't know
7	How long did the participant have to wait after arriving at the health facility before s/he was able to see the provider?<br><br>(**Ç**: *Record respondents' answer in minutes ONLY. If the waiting time was one and a half hours, record either 90 minutes*)	____<br>9999.Don't know	____<br>9999.Don't know	____<br>9999.Don't know	____<br>9999.Don't know	____<br>9999.Don't know
8	If you had not visited the health facility, would you have been working – either at home or for wages?	☐1.Yes<br>☐2.No<br>☐9.Don't know	☐1.Yes<br>☐2.No<br>☐9.Don't know	☐1.Yes<br>☐2.No<br>☐9.Don't know	☐1.Yes<br>☐2.No<br>☐9.Don't know	☐1.Yes<br>☐2.No<br>☐9.Don't know
9	Who paid for the health facility visit?<br>*(Refer to * below for response key)*					
10	What was the total amount the participant had to spend to receive the treatment at the health facility, not including the cost of food, transportation and overnight stay? *(In local currency unit)*					
11	Did you receive medication?<br>*(If "No" or "Don't know" skip to Q.14)*	☐1.Yes<br>☐2.No<br>☐9.Don't know	☐1.Yes<br>☐2.No<br>☐9.Don't know	☐1.Yes<br>☐2.No<br>☐9.Don't know	☐1.Yes<br>☐2.No<br>☐9.Don't know	☐1.Yes<br>☐2.No<br>☐9.Don't know
12	Does the out of pocket cost mentioned in Q.10 include medication cost?	☐1.Yes<br>☐2.No<br>☐9.Don't know	☐1.Yes<br>☐2.No<br>☐9.Don't know	☐1.Yes<br>☐2.No<br>☐9.Don't know	☐1.Yes<br>☐2.No<br>☐9.Don't know	☐1.Yes<br>☐2.No<br>☐9.Don't know

13	If not, how much was the cost of medication paid out of pocket? *(In local currency unit)*					
14	Did you receive any diagnostic test?<br>*(If "No" or "Don't know" skip to Q.16)*	☐1.Yes<br>☐2.No<br>☐9.Don't know	☐1.Yes<br>☐2.No<br>☐9.Don't know	☐1.Yes<br>☐2.No<br>☐9.Don't know	☐1.Yes<br>☐2.No<br>☐9.Don't know	☐1.Yes<br>☐2.No<br>☐9.Don't know
15	Does the out of pocket cost mentioned in Q.10 include diagnostic costs?	☐1.Yes<br>☐2.No<br>☐9.Don't know	☐1.Yes<br>☐2.No<br>☐9.Don't know	☐1.Yes<br>☐2.No<br>☐9.Don't know	☐1.Yes<br>☐2.No<br>☐9.Don't know	☐1.Yes<br>☐2.No<br>☐9.Don't know
16	If not, how much was the cost of diagnostics paid out of pocket? *(In local currency unit)*					
17	Did the participant stay overnight at the health facility?<br>*(If "No" or "Don't know" skip to Q.21)*	☐1.Yes<br>☐2.No<br>☐9.Don't know	☐1.Yes<br>☐2.No<br>☐9.Don't know	☐1.Yes<br>☐2.No<br>☐9.Don't know	☐1.Yes<br>☐2.No<br>☐9.Don't know	☐1.Yes<br>☐2.No<br>☐9.Don't know
18	How many nights did the participant stay?					
19	Does the out of pocket cost mentioned in Q.10 include overnight stay cost?	☐1.Yes<br>☐2.No<br>☐9.Don't know	☐1.Yes<br>☐2.No<br>☐9.Don't know	☐1.Yes<br>☐2.No<br>☐9.Don't know	☐1.Yes<br>☐2.No<br>☐9.Don't know	☐1.Yes<br>☐2.No<br>☐9.Don't know
20	*If not, how much was the cost of overnight stay paid out of pocket? (In local currency unit)*					
21	Did any **friend or family** member **accompany** the participant when you/s/he sought care?<br>*(If "No" or "Don't know" skip to Q.24)*	☐1.Yes<br>☐2.No<br>☐9.Don't know	☐1.Yes<br>☐2.No<br>☐9.Don't know	☐1.Yes<br>☐2.No<br>☐9.Don't know	☐1.Yes<br>☐2.No<br>☐9.Don't know	☐1.Yes<br>☐2.No<br>☐9.Don't know
22	How many person(s) accompanied the participant?					
23	Lodging cost for companion (if paid separately).<br>*(In local currency unit)*<br>(**Ç**: *Skip this question, if participant did not stay overnight or did have a companion to the health facility)*					
24	What form of transportation was used to the treatment provider?<br>*(Refer to ^ below for response key)*					
25	**How long** did it take to travel **one-way** to the health facility?<br>(**Ç**: *Record respondents' answer in minutes ONLY. If the waiting time was one and a half hours, record either 90 minutes*)	_______<br>9999.Don't know	_______<br>9999.Don't know	_______<br>9999.Don't know	_______<br>9999.Don't know	_______<br>9999.Don't know
26	**One-way** transportation cost for participant & companions to the health facility.<br>*(In local currency unit)*					
27	Food cost for the **participant** during the health facility visit (if paid separately).<br>*(In local currency unit)*					
28	Food cost for **companion** during the health facility visit (if paid separately).<br>*(In local currency unit)*					
29	Add-on and miscellaneous costs related to the treatment (eg. toothpaste, soap, phone, herbs, etc.). *(In local currency unit)*					

#	*Response for question B5: Option for "Type of treatment provider" above*<br>**Name codes:** a=Private hospital/ clinic b=Public health facility c=Other health facility d=Pharmacy<br>e**=**Traditional healer f=Other/self					

*	*Response option for Q.9 above:* Who paid for this visit? [Total cost]
(1) Paid completely out-of-pocket by you or your household<br>(2) Paid completely by health insurance/employer<br>(3) Paid completely by health care facility<br>(4) Paid partly by insurance and partly out-of-pocket by you or your household<br>(5) Paid partly by health care facility and partly out-of-pocket by you or your household<br>(6) Paid partly by health care facility and partly by insurance<br>(7) Paid partly by health care facility, partly by insurance, and partly out-of-pocket by you or your household<br>(8) Other *(please specify)* ____<br>(9) Don't know	
^	Response option for question B.23 above<br>1.On foot/walking 2.Motorcycle 3.Bicycle 4. Tricycle 5.Animal/cart 6.Car/taxi 7.Bus/truck<br>8.Ambulance 98.Other, specify:_________________________ 99.Don't know

INDIRECT COSTS: PARTICIPANT

☺: I want to know whether the participant needed any person to take care of him/her (**caretaker**) or whether the participant had to ask for someone else to do his/her work/job (**substitute labourer**) during the other illness. Also whether there were days participant **was completely unable**, or **able to perform some** or **able to perform all** of his/her daily routine **during the other illness.** (Ç: Please do not include the day of interview)

**C**		
30	**In total**, how many days has the participant been sick with the illness?	_____ days<br>9999=Don't know
31	How many days was the participant **completely unable to perform any** of his/her **usual activities**?	_____ days<br>9999=Don't know
32	How many days was the participant **able to perform some** of his/her **usual activities**?	_____ days<br>9999=Don't know
33	How many days was the participant **able to perform all** of his/her **activities while sick**?	_____ days<br>9999=Don't know
34	What would the participant have been doing if s/he had not been sick?<br><br>*(Check only one main activity)*	☐1.Going to school<br>☐2.Working on own farm or self-employed<br>☐3.House work (eg. Cooking, cleaning, child care giving)<br>☐4.Working for wage<br>☐5.Leisure time<br>☐8.Other, specify____________________
35	How much is the participant normally paid for one day's work?	In local currency: __________<br>99999999=Don't know
36	Did the participant lose any income because of this illness?<br>*(If "No" or "Don't know" skip to Section D, Q.38)*	☐1.Yes<br>☐2.No<br>☐9.Don't know
37	In total, how many days' income did you or the participant lose because of this illness?	_____ days<br>9999=Don't know

Error Check Box

(Interviewer: Please check that the days of complete incapacitation, days of debilitation, and normal activity days add up to the total number of days ill.)						
Days of incapacitation<br>(no activity while ill)[31]	+	Days of debilitation<br>(some activity while ill)[32]	+	Days of normal activity<br>(while ill)[33]	+	Total number of days ill (Check that this is equal to number of sick days reported by respondent)[30]
	+		+		=	

INDIRECT COSTS: SUBSTITUTE LABOR

D		
38	Did anyone perform the participant's usual activities for you or him/her while s/he was sick with the illness?<br>*(If "No" or "Don't know" skip to Section G, Q.51)*	☐1.Yes<br>☐2.No<br>☐9.Don't know
39	How many people performed your or the participant's usual activities while you or s/he was sick with that illness?	_____ Person(s)<br>9999=Don't know

Substitute number **E**	40. # The participant's relationship with the person who performed the participant's usual activities while s/he was sick.	41. Is this person an adult, teenager, or a child?<br><br>1=Adult (17+)<br>2=Teenager (12-16)<br>3=Child (Less than 12)<br>9=DK	42. How many days did s/he perform the participant's usual activities?<br>*(Record number of days and/or number of hours as days/hours)*<br><br>*Day*□ *Hour*□ 9999=DK	43. Did this person perform:<br>1=All of participant's activities<br>2=Some of participant's activities<br><br>9=DK	44. Was this person paid to perform (participant name's) activities?<br>1= Yes<br>2= No (Mark "0" in Q.45)<br>9=DK (then mark "99999999" in Q.45)	45. How much was this person paid per day?<br><br>DK=99999999<br>Enter **local currency** amount
**A**						
**B**						
**C**						
**D**						
**E**						
**F**						

#Response key for Q.40 above: 1. Spouse 2.Mother 3.Father 4.Immediate family member 5.Friend 6.Neighbour 7.Hired labourer 8.Can't say 98.Other, specify

Substitute number **F**	46. Did this person who performed the participant's activities cut back on his or her own usual activities?<br><br>1= Yes<br>2 = No<br>9 = DK<br>*(If "No" or "Don't know"skip to Section G, Q.51)*	47. How many days did this person who performed the participant's activities cut back on his or her own duties?<br>*(Record number of days and/or number of hours as days/hours)*<br><br>*Day*□ *Hour*□ 9999=DK	48. Was this person who performed the participant's activities able to do:<br><br>1=Some of his/her own activities<br>2=None of his/her own activities	49. What would this person who performed the participant's activities have been doing if s/he had not been performing the participant's activities while s/he was sick?<br>1=Going to school<br>2 = Working on a farm<br>3 =Working at home<br>4 =Working for a wage<br>5 =Leisure time<br>8=Other, *specify*	50. *(If s/he would have worked for a wage)* How much is this person who performed the participant's activities normally paid for one day's work?<br><br>DK=99999999<br>Enter **local currency** amount
**A**					
**B**					
**C**					
**D**					
**E**					
**F**					

INDIRECT COSTS: CARETAKING

G		
51	Was the participant so sick that someone had to cut back on his or her own usual activities for one or more days just to care for him/her?<br><br>*Caretaking means assuming responsibility for the physical and emotional needs of a minor or health impaired participant because they are incapable of self-care.*<br>*Care taking activities can include attending to the participant at home, accompanying the participant during trips to the* [name study facility:_________________________] *or other care providers or running errands for the participant such as picking up medication.*<br>*(If "No" or "Don't know" skip to Section K, Q.65)*	☐ 1.Yes<br>☐2.No<br>☐9.Don't know
52	How many people cared for the participant while s/he was sick?	_____ Person(s)<br>9999=Don't know

Caretaker number H-1	53.* The participant's relationship with the person who cared for the participant while s/he was sick.	54. Is this person an adult, teenager, or a child?<br>1=Adult(17+yrs)<br>2=Teenager (12-16 years)<br>3=Child (<12yrs)<br>9=DK	55. How many days did he/she care for the participant?<br>*(Record number of days and/or number of hours as days/hours)*<br>*Day*☐ *Hour*☐ 99=DK	56. Was this person paid to care for the participant?<br>1= *Yes*<br>2= No *(Mark "0" in Q.57)*<br>9= DK *(Mark 99999999 in Q.57)*	57. How much was this person paid per day to care for the participant?<br>(DK=99999999)<br>Enter **local currency** amount
A					
B					
C					
D					
E					
F					

*Response option for Q.53 above: 1. Spouse 2.Mother 3.Father 4.Immediate family member 5.Friend 6.Neighbour 7.Hired labourer 8.Can't say 98.Other, specify

Caretaker number H-2	58. Did this person who cared for the participant cut back on his or her own usual activities?<br><br>1= *Yes*<br>2 = No<br>9=DK<br>*(If "No" or "Don't know" skip to Section K, Q.65)*	59. How many days did this person who cared for the participant cut back on his or her own duties?<br>*(Record number of days and/or number of hours as days/hours)*<br><br>*Day*☐ *Hour*☐ 99=DK	60. Was this person who cared for the participant able to do:<br><br>1=Some of his/her own activities<br>2=None of his/her own activities<br>9=DK	61. What would this person who cared for the participant have been doing if he/she had not been caring for the participant while s/he was sick?<br><br>1= Going to school<br>2 = Working on a farm<br>3 = Working at home<br>4 = Working for a wage<br>5 = Leisure time<br>8=Other, *specify*	62. *(If he/she would have worked for a wage)*<br>How much is this person who cared for the participant normally paid for one day's work?<br><br>DK=99999999<br>Enter **local currency** amount
**A**					
**B**					
**C**					
**D**					
**E**					
**F**					

**I**		
63	Is the participant still sick with the other illness?	☐1.Yes ☐2.No ☐3.Can't say<br>*If "**YES**" or "**Can't say**", schedule next interview after finishing to administer all applicable questionnaires on this interview.*<br>*If "**No**", end COOI follow-up after finishing to administer all applicable questionnaires on this interview.*

**J**	**Ç: FOR YOU THE INTERVIEWER TO TAKE ACTION**
64	Upon completion of this form:<br>Use F6.1: LT-SES Survey Form for Cases' Initial Interview if the participant is a *S.* Typhi/Special Case.<br>OR<br>Use F6.3: LT-SES Survey Form Neighbourhood Controls' Initial Interview if the participant is a neighbourhood Control.

Annex 4: SETA COOI Follow-up Interview Tool

Form F5: COOI Follow-Up Interview

(Day ___ to ___: ___☐week/☐month post enrollment by illness continuation)

F5 for Special case (Day 3-7 and beyond), *S.* Typhi cases and neighbourhood controls (Day 12-14 and beyond), then if (any) other illness continues

Note to Interviewer
*If you see **"Ç",** that is a **note for you** to be read by yourself. If you see **"☺"** you should **read out loud** for the respondent or the caretaker/Next of kin.*

Site/**C**ountry: ______________________________/______________________________

Follow-up Schedule: ☐Day 3-7 (for special cases only)

☐Day 12-14 ☐Day 28-30 ☐Day 90 ☐Day 180 ☐Day 270 ☐Day 360

Interviewer's Name:_________________________ **D**ate of Interview (dd/mm/yyyy): _____/_____/__________

Laboratory diagnosis: ☐*S.* Typhi ☐Special cases ☐*S.* Paratyphi ☐iNTS

☐Clinical enteric fever ☐**C**ontrol ☐**O**ther illness of the participant (if any): ____________________

Place of interview: ☐Health facility **/** ☐Home **/** ☐Other, specify: ______________________________

If at health facility, indicate point of care: ☐Out-Patient / ☐In-Patient;

If In-Patient, name of admission ward: ____________________

Date of Admission (dd/mm/yyyy): _____/_____/__________

Date of Discharge (dd/mm/yyyy): _____/_____/__________ [**Ç:** Look this up in the study facility record]

**Ç: Check whether participant completed the diary card.**	
**L**ogbook (Diary card) completed?	☐1.Yes ☐2.No

Cost-of-Illness Questionnaire: ___- ___ day Follow-Up

Ç: If Form F3 has been administered to the participant before this interview, skip this section and go to Section A. If Not then use this part for the participant and continue to **Section A, Q.1**.

***Ç:** Is the participant the right person interviewed ____ ☐weeks / ☐months (____ to ____ days) ago.*		
0	Correct?	☐1.Yes ☐2.No ☐9.Don't know
***Ç:** If "**No**" or "**Don't know**", identify the right candidate to continue the interview.*		

***Ç:** Use this instrument to interview an adult participant/caretaker/caretaker/Next of kin(in case of a minor or if health-impaired) that is familiar with the participant and his/her treatment of other illness over the last few days/weeks/months.*
**☺:** I am going to ask you about the participant's <u>other illness</u> and the costs that the participant/their household incurred for the management of that illness until yesterday.

**☺:** Please refer to your logbook (diary card) recorded when you were/the participant was sick.
***Ç:** If the respondent has their logbook(diary card) before them. ☺ Please check your logbook if it will help you remember.*

☺***<u>Please think about what happened when you were sick with the other illness. Please tell me only about this condition.</u>***

DIRECT COSTS: Health facility/clinic visits until yesterday before this interview

**A**	☺ ***First, I would like to know how this disease episode affected the participant and his/her household financially. Ç: Please, cross check with the participant's diary card to see if answers are comparable.***	
1	Did the participant receive treatment for this illness **at the study health facility or any health provider** from the **day of our last interview (including the day of that interview) until yesterday**?<br>*(If "No" or "Don't know" skip to Section B, Q.4)*	☐1.Yes<br>☐2.No<br>☐9.Don't know
2	Which types of health provider has the participant visited? *(Select all that apply)*<br>*(Refer to ++ below for response key)*	_____ _____ _____ _____ _____ _____
3	How many times has the participant made a visit **in total**?	_____x _____x _____x _____x _____x _____x
++	***Response for A2: Option for "Type of health provider" above***<br>**Name codes:** a=Private hospital/ clinic b=Public health facility c=Other health facility d=Pharmacy e=Traditional healer f=Other/self	

DETAILS OF HEALTH FACILITY/CLINIC VISITS FROM THE DAY OF LAST INTERVIEW TO A DAY BEFORE THIS INTERVIEW (YESTERDAY)

*For the participant who has received treatment for **other illness** from all health care facilities/clinics, please provide following details. Each column numbered 1,2,3… represents one care provider visited. Do not report payments made by insurance company or program. I want to know how much you or your household had to spend during each visit and other things.*

**B**	**All health facility visits from the day of last interview till yesterday (Day ___ - ___)**	**Visit**				
		**Day of last interview to day before this interview**				
		1	2	3	4	5
4	Date of visit<br>dd/<br>mm/<br>yyyy	_____/<br>_____/<br>_____	____/<br>____/<br>____	____/<br>____/<br>____	____/<br>____/<br>____	____/<br>____/<br>____
5	Type of treatment provider<br>*(Refer to # below for response key)*					
6	Was the participant's visit for SETA research purpose?	☐1.Yes<br>☐2.No<br>☐9.Don't know	☐1.Yes<br>☐2.No<br>☐9.Don't know	☐1.Yes<br>☐2.No<br>☐9.Don't know	☐1.Yes<br>☐2.No<br>☐9.Don't know	☐1.Yes<br>☐2.No<br>☐9.Don't know
7	How long did the participant have to wait after arriving at the health facility before s/he was able to see the provider?<br><br>(Ç: *Record respondents' answer in minutes ONLY. If the waiting time was one and a half hours, record either 90 minutes*)	_______<br><br>9999.Don't know	_______<br><br>9999.Don't know	_______<br><br>9999.Don't know	_______<br><br>9999.Don't know	_______<br><br>9999.Don't know
8	If you had not visited the health facility, would you have been working – either at home or for wages?	☐1.Yes<br>☐2.No<br>☐9.Don't know	☐1.Yes<br>☐2.No<br>☐9.Don't know	☐1.Yes<br>☐2.No<br>☐9.Don't know	☐1.Yes<br>☐2.No<br>☐9.Don't know	☐1.Yes<br>☐2.No<br>☐9.Don't know
9	Who paid for the health facility visit?<br>*(Refer to * below for response key)*					
10	What was the total amount the participant had to spend to receive the treatment at the health facility, not including the cost of food, transportation and overnight stay? *(In local currency unit)*					
11	Did you receive medication?<br>*(If "No" or "Don't know" skip to Q.14)*	☐1.Yes<br>☐2.No<br>☐9.Don't know	☐1.Yes<br>☐2.No<br>☐9.Don't know	☐1.Yes<br>☐2.No<br>☐9.Don't know	☐1.Yes<br>☐2.No<br>☐9.Don't know	☐1.Yes<br>☐2.No<br>☐9.Don't know
12	Does the out of pocket cost mentioned in Q.10 include medication cost?	☐1.Yes<br>☐2.No<br>☐9.Don't know	☐1.Yes<br>☐2.No<br>☐9.Don't know	☐1.Yes<br>☐2.No<br>☐9.Don't know	☐1.Yes<br>☐2.No<br>☐9.Don't know	☐1.Yes<br>☐2.No<br>☐9.Don't know

13	If not, how much was the cost of medication paid out of pocket? *(In local currency unit)*					
14	Did you receive any diagnostic test?<br>*(If "No" or "Don't know" skip to Q.17)*	☐1.Yes<br>☐2.No<br>☐9.Don't know	☐1.Yes<br>☐2.No<br>☐9.Don't know	☐1.Yes<br>☐2.No<br>☐9.Don't know	☐1.Yes<br>☐2.No<br>☐9.Don't know	☐1.Yes<br>☐2.No<br>☐9.Don't know
15	Does the out of pocket cost mentioned in Q.10 include diagnostic costs?	☐1.Yes<br>☐2.No<br>☐9.Don't know	☐1.Yes<br>☐2.No<br>☐9.Don't know	☐1.Yes<br>☐2.No<br>☐9.Don't know	☐1.Yes<br>☐2.No<br>☐9.Don't know	☐1.Yes<br>☐2.No<br>☐9.Don't know
16	If not, how much was the cost of diagnostics paid out of pocket? *(In local currency unit)*					
17	Did you stay overnight at the health facility?<br>*(If "No" or "Don't know" skip to Q.21)*	☐1.Yes<br>☐2.No<br>☐9.Don't know	☐1.Yes<br>☐2.No<br>☐9.Don't know	☐1.Yes<br>☐2.No<br>☐9.Don't know	☐1.Yes<br>☐2.No<br>☐9.Don't know	☐1.Yes<br>☐2.No<br>☐9.Don't know
18	How many nights did you stay?					
19	Does the out of pocket cost mentioned in Q.10 include overnight stay cost?	☐1.Yes<br>☐2.No<br>☐9.Don't know	☐1.Yes<br>☐2.No<br>☐9.Don't know	☐1.Yes<br>☐2.No<br>☐9.Don't know	☐1.Yes<br>☐2.No<br>☐9.Don't know	☐1.Yes<br>☐2.No<br>☐9.Don't know
20	If not, how much was the cost of overnight stay paid out of pocket? *(In local currency unit)*					
21	Did any **friend or family** member **accompany** the participant when s/he sought care?<br>*(If "No" or "Don't know" skip to Q.24)*	☐1.Yes<br>☐2.No<br>☐9.Don't know	☐1.Yes<br>☐2.No<br>☐9.Don't know	☐1.Yes<br>☐2.No<br>☐9.Don't know	☐1.Yes<br>☐2.No<br>☐9.Don't know	☐1.Yes<br>☐2.No<br>☐9.Don't know
22	How many person(s) accompanied the participant?					
23	Lodging cost for companion (if paid separately).<br>(In local currency unit)<br>(**Ç**: *Skip this question, if participant did not stay overnight or did have a companion to the health facility)*					
24	What form of transportation was used to the treatment provider?<br>*(Refer to ^ below for response key)*					
25	**How long** did it take to travel **one-way** to the health facility?<br>(**Ç**: *Record respondents' answer in minutes ONLY. If the waiting time was one and a half hours, record either 90 minutes*)	________<br>9999.Don't know	________<br>9999.Don't know	________<br>9999.Don't know	________<br>9999.Don't know	________<br>9999.Don't know
26	**One-way** transportation cost for participant & companions to the health facility. *(In local currency unit)*					
27	Food cost for **participant** during the health facility visit (if paid separately). *(In local currency unit)*					
28	Food cost for **companion** during the health facility visit (if paid separately). *(In local currency unit)*					
29	Add-on and miscellaneous cost related to the treatment (eg. toothpaste, soap, phone, herbs,					

	etc.). *(In local currency unit)*					
#	*Response for question B5: Option for "Type of treatment provider" above*<br>**Name codes:** a=Private hospital/ clinic b=Public health facility c=Other health facility d=Pharmacy<br>e=Traditional healer f=Other/self					

*	*Response option for question B9 above:* Who paid for this visit? [Total cost]
(1) Paid completely out-of-pocket by you or your household<br>(2) Paid completely by health insurance/employer<br>(3) Paid completely by health care facility<br>(4) Paid partly by insurance and partly out-of-pocket by you or your household<br>(5) Paid partly by health care facility and partly out-of-pocket by you or your household<br>(6) Paid partly by health care facility and partly by insurance<br>(7) Paid partly by health care facility, partly by insurance, and partly out-of-pocket by you or your household<br>(8) Other *(please specify)* ____<br>(9) Don't know	
^	Response option for question B23 above<br>1.On foot/walking 2.Motorcycle 3.Bicycle 4. Tricycle 5.Animal/cart 6.Car/taxi 7.Bus/truck<br>8.Ambulance 98.Other, specify:_________________________ 99.Don't know

INDIRECT COSTS: PARTICIPANT

☺: I want to know whether the participant needed any person to take care of him/her (**caretaker**) or whether the participant had to ask for someone else to do his/her work/job(**substitute labourer**) during the other illness. Also whether there were days the participant **was completely unable**, or **able to perform some** or **able to perform all** of his/her daily routine **during the other illness**. (Ç: Please do not include the day of interview)

**C**	*Ç: Please, cross check with the participant's diary card to see if answers are comparable.*	
30	**In total**, how many days has the participant been sick with the illness since the last interview?	_____ days<br>9999=Don't know
31	How many days was the participant **completely unable to perform any** of his/her **usual activities**?	_____ days<br>9999=Don't know
32	How many days was the participant **able to perform some** of his/her **usual activities**?	_____ days<br>9999=Don't know
33	How many days was the participant **able to perform all** of his/her **activities while sick**?	_____ days<br>9999=Don't know
34	What would the participant have been doing if s/he had not been sick?<br><br>*(Check only one main activity)*	☐1.Going to school<br>☐2.Working on own farm or self-employed<br>☐3.House work (eg. Cooking, cleaning, child care giving)<br>☐4.Working for wage<br>☐5.Leisure time<br>☐8.Other, specify_____________________
35	How much is the participant normally paid for one day's work?	In local currency: ___________<br>99999999=Don't know

36	Did the participant lose any income because of this illness?<br>*(If "No" or "Don't know" skip to Section D, Q.38)*	☐1.Yes<br>☐2.No<br>☐9.Don't know
37	In total, how many days' income did the participant lose because of this illness?	______ days<br>9999=Don't know

Error Check Box

(Ç: Please check that the days of complete incapacitation, days of debilitation, and normal activity days add up to the total number of days ill.)						
Days of incapacitation<br>(no activity while ill)[31]	+	Days of debilitation<br>(some activity while ill)[32]	+	Days of normal activity<br>(while ill)[33]	+	Total number of days ill (Check that this is equal to number of sick days reported by respondent)[30]
	+		+		=	

INDIRECT COSTS: SUBSTITUTE LABOR

D		
38	Did anyone perform the participant's usual activities for you or him/her while s/he was sick with the illness?<br>*(If "No" or "Don't know" skip to Section G, Q.51)*	☐1.Yes<br>☐2.No<br>☐9.Don't know
39	How many people performed your or the participant's usual activities while you or s/he was sick with that illness?	_____ Person(s)<br>9999=Don't know

Substitute number E	40. # The participant's relationship with the person who performed the participant's usual activities while s/he was sick.	41. Is this person an adult, teenager, or a child?<br><br>1=Adult (17+)<br>2=Teenager (12-16)<br>3=Child (Less than 12)<br>9=DK	42. How many days did s/he perform the participant's usual activities? *(Record number of days and/or number of hours as days/hours)*<br><br>*Day*☐ *Hour*☐<br>9999=DK	43. Did this person perform:<br>1=All of participant's activities<br>2=Some of participant's activities<br><br>9=DK	44. Was this person paid to perform activities?<br><br>1= Yes<br>2= No (Mark "0" in Q.45)<br>9=DK (then mark "99999999" in Q.45)	45. How much was this person paid per day?<br><br>DK=99999999<br>Enter **local currency** amount
**A**						
**B**						
**C**						
**D**						
**E**						
**F**						

#Response key for Q.40 above: 1. Spouse 2.Mother 3.Father 4.Immediate family member 5.Friend 6.Neighbour 7.Hired labourer 8.Can't say 98.Other, specify

Substitute number F	46. Did this person who performed the participant's activities cut back on his or her own usual activities?<br><br>1= Yes<br>2 = No<br>9 = DK<br>*(If "No" or "Don't know"skip to G.51)*	47. How many days did this person who performed the participant's activities cut back on his or her own duties? *(Record number of days and/or number of hours as days/hours)*<br><br>*Day*☐ *Hour*☐ 9999=DK	48. Was this person who performed the participant's activities able to do:<br><br>1=Some of his/her own activities<br>2=None of his/her own activities	49. What would this person who performed the participant's activities have been doing if s/he had not been performing the participant's activities while s/he was sick?<br>1=Going to school<br>2 = Working on a farm<br>3 =Working at home<br>4 =Working for a wage<br>5 =Leisure time<br>8=Other, *specify*	50. *(If s/he would have worked for a wage)* How much is this person who performed the participant's activities normally paid for one day's work?<br><br>DK=99999999<br>Enter **local currency** amount
**A**					
**B**					
**C**					
**D**					
**E**					
**F**					

INDIRECT COSTS: CARETAKING

G		
51	Was the participant so sick that someone had to cut back on his or her own usual activities for one or more days just to care for him/her?<br><br>*Caretaking means assuming responsibility for the physical and emotional needs of a minor or health impaired participant because they are incapable of self-care. Caretaking activities can include attending to the participant at home, accompanying the participant during trips to the* [name study facility:_________________________] *or other care providers or running errands for the participant such as picking up medication.*<br>*(If "No" or "Don't know" skip to Section J, Q.63)*	☐ 1.Yes<br>☐2.No<br>☐9.Don't know
52	How many people cared for the participant while s/he was sick?	_____ Person(s)<br>9999=Don't know

**Caretaker number H-1**	53.*<br>The participant's relationship with the person who cared for the participant while s/he was sick.	54.<br>Is this person an adult, teenager, or a child?<br><br>1=Adult(17+yrs)<br>2=Teenager (12-16 years)<br>3=Child (<12yrs)<br>9=DK	55.<br>How many days did he/she care for the participant?<br><br>*(Record number of days and/or number of hours as days/hours)*<br>*Day*☐ *Hour*☐ 99=DK	56.<br>Was this person paid to care for the participant?<br><br>1= *Yes*<br>2= No *(Mark "0" in Q.57)*<br>9= DK *(Mark 99999999 in Q.57)*	57.<br>How much was this person paid per day to care for the participant?<br><br>DK=99999999<br>Enter **local currency** amount
**A**					
**B**					
**C**					
**D**					
**E**					
**F**					

*Response key for Q.53 above: 1. Spouse 2.Mother 3.Father 4.Immediate family member 5.Friend 6.Neighbour 7.Hired labourer 8.Can't say 98.Other, specify

**Caretaker number H-2**	58. Did this person who cared for the participant cut back on his or her own usual activities?<br><br>1= *Yes*<br>2 = No<br>9=DK<br><br>*(If "No" or "Don't know" skip to Section J, Q.63)*	59. How many days did this person who cared for the participant cut back on his or her own duties? *(Record number of days and/or number of hours as days/hours)*<br><br>*Day*☐ *Hour*☐<br>99=DK	60. Was this person who cared for the participant able to do:<br><br>1=Some of his/her own activities<br>2=None of his/her own activities<br>9=DK	61. What would this person who cared for the participant have been doing if he/she had not been caring for the participant while s/he was sick?<br><br>1= Going to school<br>2 = Working on a farm<br>3 = Working at home<br>4 = Working for a wage<br>5 = Leisure time<br>8=Other, *specify*	62. *(If he/she would have worked for a wage)*<br>How much is this person who cared for the participant normally paid for one day's work?<br><br>DK=99999999<br>Enter **local currency** amount
**A**					
**B**					
**C**					
**D**					
**E**					
**F**					

**I**	**FRICTION COST**	
63	You were sick for ___ to ___ days now, and had many unfulfilled tasks. Do you have to work extra to compensate for the missed tasks due to the illness?	☐1.Yes<br>☐2.No.
64	If YES, by working extra do you think you will complete all the tasks which you have missed?	☐1.Yes<br>☐2.No.

**J**		
65	Is the participant still sick with the other illness?	☐1.Yes ☐2.No ☐3.Can't say<br>*If "**YES**" or "**Can't say**", schedule next interview after finishing to administer all applicable questionnaires on this interview.*<br>*If "**No**", end COOI follow-up after finishing to administer all applicable questionnaires on this interview.*

**K**	**Ç: FOR YOU THE INTERVIEWER TO TAKE ACTION**
66	Upon completion of this form:<br>Use the F6.2: LT-SES Survey Form Cases and Controls' Follow-Up for either *S.* Typhi/Special Case or a neighbourhood Control.

Annex 5: SETA QoL Tool

Form F0: Quality of Life Survey (QoL)

(Day 0: For all febrile and special case participants at SETA enrollment)

F0 for collecting data on participant's wellbeing at enrollment.

Note to Interviewer
*If you see **"Ç",** that is a **note for you** to be read by yourself. If you see **"☺"** you should **read out loud** for the respondent or the caretaker/Next of kin.*

Site/**C**ountry: ____/______________________________

Follow-up Schedule: ☐Day 0 of SETA enrollment

Interviewer's Name:_________________________ **D**ate of Interview (dd/mm/yyyy): _____/_____/__________

Indicate point of care: ☐Out-Patient / ☐In-Patient;

If In-Patient, name of admission ward: ____________________

Date of Admission (dd/mm/yyyy): _____ /_____/__________

Participant's Information

Ç: *Refer to SETA-Appendix B1_Patient Enrollment form number 8 to 13 and fill this part*

Participant's contact number: 1.__________________________ 2.__________________________

Current address/landmarks: ____

Caretaker/Next of kin's Information

Caretaker/Next of kin's Phone: 1. _______________ 2. _______________ 3. _______________

Caretaker/Next of kin's relation to patient: ☐Spouse ☐Mother ☐Father ☐Immediate family member ☐Friend ☐Neighbour ☐Hired individual ☐Don't know ☐Other; specify: ____________________

Ç: *Use this instrument to interview an adult participant/caretaker/next of kin (in case of a minor or if health-impaired) that is familiar with the participant's wellbeing.*
☺ Now I am going to ask about the participant's **current** health state on general, physical, emotional, social wellbeing.

A: MEDICAL OUTCOME SURVEY FOR PARTICIPANT (ADOPTED FROM SF-36)

Participant/Caretaker/Next of Kin (In case of minor or if health impaired)

	☺ Please select ONLY one response for each question and for sub-questions ONLY one for each line			
1	In general, would you say your health is:	☐1.Excellent ☐2.Very good ☐3.Good ☐4.Fair ☐5.Poor		
2	**Compared to the time before the illness started**, how would you rate your health in general **now**?	☐1.Much better now than before the illness started<br>☐2.Somewhat better now than before the illness started<br>☐3.About the same<br>☐4.Somewhat worse now than before the illness started<br>☐5.Much worse than before the illness started		
	The following items are about activities you might do during a typical day. Does **your health now limit you** in these activities? If so, how much?	**Yes, limited a lot**	**Yes, limited a little**	**No, not limited at all**
3	**Vigorous activities**, such as running, lifting heavy objects, participating in strenuous sports	☐1.	☐2.	☐3.
4	**Moderate activities**, such as moving a table, pushing a vacuum cleaner, bowling, or playing golf	☐1.	☐2.	☐3.
5	Lifting or carrying groceries	☐1.	☐2.	☐3.
6	Climbing **several** flights of stairs	☐1.	☐2.	☐3.
7	Climbing **one** flight of stairs	☐1.	☐2.	☐3.
8	Bending, kneeling or stooping	☐1.	☐2.	☐3.
9	Walking **more than a mile**	☐1.	☐2.	☐3.
10	Walking **several blocks**	☐1.	☐2.	☐3.
11	Walking **one block**	☐1.	☐2.	☐3.
12	Bathing or dressing yourself	☐1.	☐2.	☐3.
	**From the day the illness started,** have you had any of the following problems with your work or regular daily activities **as a result of physical health**?		**Yes**	**No**
13	Cut down the amount of time you spent on work or other activities		☐1.	☐2.
14	**Accomplished less** than you would		☐1.	☐2.
15	Were limited in the **kind** of work or other activities		☐1.	☐2.
16	Had **difficulty** performing the work or other activities (for example, it took extra effort)		☐1.	☐2.
	**From the day the illness started,** have you had any of the following problems with your work or other regular daily activities **as a result of any emotional problems** (such as feeling depressed or anxious)?			
17	Cut down the **amount of time** you spent on work or other activities		☐1.	☐2.
18	**Accomplished less** than you would like		☐1.	☐2.
19	Didn't do work or other activities as **carefully** as usual		☐1.	☐2.

20	**From the day the illness started,** to what extent has your physical health or emotional problems interfered with your normal social activities with family, friends, neighbours or groups?	Not at all ☐1.	Slightly ☐2.	Moderately ☐3.	Quite a bit ☐4.	Extremely ☐5.

21	How much **bodily** pain have you had **from the day the illness started?**	None ☐1.	Very mild ☐2.	Mild ☐3.	Moderate ☐4.	Severe ☐5.	Very severe ☐6.

22	**From the day the illness started**, how much did **pain** interfere with your normal work (including both work outside the home and housework)?	Not at all ☐1.	A little bit ☐2.	Moderately ☐3.	Quite a bit ☐4.	Extremely ☐5.

These questions are about how you feel and how things have been with you **from the day the illness started**. For each question, please give the one answer that comes closest to the way you have been feeling.

	How much of the time from the day the illness started …	All the time	Most of the time	A good bit of time	Some of the time	A little bit of time	None of the time
23	Did you feel full of pep?	☐1.	☐2.	☐3.	☐4.	☐5.	☐6.
24	Have you been a very nervous person?	☐1.	☐2.	☐3.	☐4.	☐5.	☐6.
25	Have you felt so down in the dumps that nothing could cheer you up?	☐1.	☐2.	☐3.	☐4.	☐5.	☐6.
26	Have you felt calm and peaceful?	☐1.	☐2.	☐3.	☐4.	☐5.	☐6.
27	Did you have a lot of energy?	☐1.	☐2.	☐3.	☐4.	☐5.	☐6.

28	Have you felt downhearted and blue?	☐1.	☐2.	☐3.	☐4.	☐5.	☐6.
29	Did you feel worn out?	☐1.	☐2.	☐3.	☐4.	☐5.	☐6.
30	Have you been happy person?	☐1.	☐2.	☐3.	☐4.	☐5.	☐6.
31	Did you feel tired?	☐1.	☐2.	☐3.	☐4.	☐5.	☐6.

32	**From the day the illness started** , how much of the time has your **physical health or emotional problems** interfered with your social activities (like visiting friends, relatives, etc.)?	All of the time ☐1.	Most of the time ☐2.	Some of the time ☐3.	A little of the time ☐4.	None of the time ☐5.

	How **TRUE** or **FALSE** is each of the following statements for you?	Definitely true	Mostly true	Don't know	Mostly false	Definitely false
33	I seem to get sick a little easier than other people	☐1.	☐2.	☐3.	☐4.	☐5.
34	I am as healthy as anybody I know	☐1.	☐2.	☐3.	☐4.	☐5.
35	I expect my health to get worse	☐1.	☐2.	☐3.	☐4.	☐5.
36	My health is excellent	☐1.	☐2.	☐3.	☐4.	☐5.

Note: The IVI acknowledges that the RAND 36-Item Short Form Health Survey was developed at RAND as part of the Medical Outcome Study.

Scale and items: Physical functioning 3,4,5,6,7,8,9,10,11,12; Role limitation due to physical health 13,14,15,16; Role limitation due to emotional problems 17,18,19; Energy/fatigue 23,27,29,30; Emotional well-being 24,25,26,28,30; Social functioning 20,32; Pain 21,22; General health 1,33,34,35,36

CONCLUSION

B	CONCLUSION

☺ That completes our interview. Thank you very much for taking the time to answer our questions. If you agree, if the doctor/nurse suggests including you in our subsequent survey we will contact you in 3 to 7 days depending on blood culture results availability. When that happens we will need to conduct another interview with you / your caretaker; the interview could be done here at this facility [enter facility name: ____________________________] or at your home depending on how you feel about it.

Annex 6: SETA LT-SES Initial Interview for Cases Tool

F6.1: LT-SES Survey Form for Cases' Initial Interview

(Day 3 to 7: For *S*. Typhi and Special Case Participants)

F6.1 for *S*. Typhi and special cases at initial interview

Note to Interviewer
If you see ***"Ç",*** *that is a* ***note for you*** *to be read by yourself. If you see* ***"☺"*** *you should* ***read out loud*** *for the respondent or the caretaker/next of kin.*

Site/**C**ountry: ______________________________ / ____

Follow-up Schedule: ☐Day 3-7 (initial interview)

Interviewer's Name: ______________________________ **D**ate of Interview (dd/mm/yyyy): _____/_____/__________

Laboratory diagnosis: ☐*S*. Typhi ☐Special cases

Place of interview: ☐Health facility / ☐Home / ☐Other, specify________________________

If at health facility, indicate point of care: ☐Out-Patient / ☐In-Patient / ☐Invited visit for SETA research

If In-Patient, name of admission ward: ___________________________________

Date of Admission: _____/_____/__________

Date of Discharge (dd/mm/yyyy): _____/_____/__________ [**Ç:** Look this up in the study facility record]

PARTICIPATION ASSESSMENT SCREENING

**Ç:** *Is the participant the right person interview last time?*		
0	Correct?	☐1.Yes ☐2.No ☐9.Don't know
**Ç:** *If "**No**" or "**Don't know**", identify the right candidate to continue the interview.*		

**Ç:** *Use this instrument to interview an adult participant/caretaker/next of kin(in case of a minor or if health-impaired) that is familiar with the participant's wellbeing.*
☺ Now I am going to ask about the participant's **current** health state on general, physical, emotional, social wellbeing.

A: MEDICAL OUTCOME SURVEY FOR ADULT PARTICIPANT

Participant/Caretaker/Next of Kin(In case of minor or if health impaired)

	Ç: Read the response options for the study participants to choose one.<br>☺ Please select ONLY one response for each question and for sub-questions ONLY one for each line			
1	In general, would you say your health is:	☐1.Excellent ☐2.Very good ☐3.Good ☐4.Fair ☐5.Poor		
2	**From the day of last interview**, how would you rate your health in general **now**?	☐1.Much better now than the day of last interview<br>☐2.Somewhat better now than the day of last interview<br>☐3.About the same<br>☐4.Somewhat worse now than the day of last interview<br>☐5.Much worse than the day of last interview		
	The following items are about activities you might do during a typical day. Does **your health now limit you** in these activities? If so, how much?	Yes, limited a lot	Yes, limited a little	No, not limited at all
3	**Vigorous activities**, such as running, lifting heavy objects, participating in strenuous sports	☐1.	☐2.	☐3.
4	**Moderate activities**, such as moving a table, pushing a vacuum cleaner, bowling, or playing golf	☐1.	☐2.	☐3.
5	Lifting or carrying groceries	☐1.	☐2.	☐3.
6	Climbing **several** flights of stairs	☐1.	☐2.	☐3.
7	Climbing **one** flight of stairs	☐1.	☐2.	☐3.
8	Bending, kneeling or stooping	☐1.	☐2.	☐3.
9	Walking **more than a mile**	☐1.	☐2.	☐3.
10	Walking **several blocks**	☐1.	☐2.	☐3.
11	Walking **one** block	☐1.	☐2.	☐3.
12	Bathing or dressing yourself	☐1.	☐2.	☐3.
	**From the day of last interview**, did you have any of the following problems with your work or regular daily activities **as a result of your physical health**?		**Yes**	**No**
13	Cut down the amount of time you spent on work or other activities		☐1.	☐2.
14	Accomplished less than you would		☐1.	☐2.
15	Were limited in the kind of work or other activities		☐1.	☐2.
16	Had difficulty performing the work or other activities (for example, it took extra effort)		☐1.	☐2.
	**From the day of last interview**, have you had any of the following problems with your work or other regular daily activities **as a result of any emotional problems** (such as feeling depressed or anxious)?		**Yes**	**No**
17	Cut down the **amount of time** you spent on work or other activities		☐1.	☐2.
18	**Accomplished less** than you would like		☐1.	☐2.
19	Didn't do work or other activities as **carefully** as usual		☐1.	☐2.

20	**From the day of last interview**, to what extent has your physical health or emotional problems interfered with your normal social activities with family, friends, neighbours or groups?	Not at all ☐1.	Slightly ☐2.	Moderately ☐3.	Quite a bit ☐4.	Extremely ☐5.

21	How much **bodily** pain have you had **from the day of last interview**?	None ☐1.	Very mild ☐2.	Mild ☐3.	Moderate ☐4.	Severe ☐5.	Very severe ☐6.

22	**From the day of last interview**, how much did **pain** interfere with your normal work (including both work outside the home and housework)?	Not at all ☐1.	A little bit ☐2.	Moderately ☐3.	Quite a bit ☐4.	Extremely ☐5.

	These questions are about how you feel and how things have been with you **from the day of the last interview**. For each question, please give the one answer that comes closest to the way you have been feeling.						
	How much of the time from the day of the last interview…	All the time	Most of the time	A good bit of time	Some of the time	A little bit of time	None of the time
23	Did you feel full of pep?	☐1.	☐2.	☐3.	☐4.	☐5.	☐6.

24	Have you been a very nervous person?	☐1.	☐2.	☐3.	☐4.	☐5.	☐6.
25	Have you felt so down in the dumps that nothing could cheer you up?	☐1.	☐2.	☐3.	☐4.	☐5.	☐6.
26	Have you felt calm and peaceful?	☐1.	☐2.	☐3.	☐4.	☐5.	☐6.
27	Did you have a lot of energy?	☐1.	☐2.	☐3.	☐4.	☐5.	☐6.
28	Have you felt downhearted and blue?	☐1.	☐2.	☐3.	☐4.	☐5.	☐6.
29	Did you feel worn out?	☐1.	☐2.	☐3.	☐4.	☐5.	☐6.
30	Have you been happy person?	☐1.	☐2.	☐3.	☐4.	☐5.	☐6.
31	Did you feel tired?	☐1.	☐2.	☐3.	☐4.	☐5.	☐6.

32	**From the day of the last interview**, how much of the time has your **physical health or emotional problems** interfered with your social activities (like visiting friends, relatives, etc.)?	All of the time ☐1.	Most of the time ☐2.	Some of the time ☐3.	A little of the time ☐4.	None of the time ☐5.

	How **TRUE** or **FALSE** is **each** of the following statements for you?	Definitely true	Mostly true	Don't know	Mostly false	Definitely false
33	I seem to get sick a little easier than other people	☐1.	☐2.	☐3.	☐4.	☐5.
34	I am as healthy as anybody I know	☐1.	☐2.	☐3.	☐4.	☐5.
35	I expect my health to get worse	☐1.	☐2.	☐3.	☐4.	☐5.
36	My health is excellent	☐1.	☐2.	☐3.	☐4.	☐5.

Note: The IVI acknowledges that the RAND 36-Item Short Form Health Survey was developed at RAND as part of the Medical Outcome Study.

Scale and items: Physical functioning 3,4,5,6,7,8,9,10,11,12; Role limitation due to physical health 13,14,15,16; Role limitation due to emotional problems 17,18,19; Energy/fatigue 23,27,29,30; Emotional well-being 24,25,26,28,30; Social functioning 20,32; Pain 21,22; General health 1,33,34,35,36

B: FINANCIAL BURDEN

	**Financial Burden for Participants**<br>***Ҁ: Following questions do not include lost work and lost income related to caretaking and substitute labour during illness***	
37	**From the day your illness started,** did the participant's household have to borrow money from anyone in order to pay for treatment of the illness?<br>*(If "No" and "Don't know" skip to Q.39)*	☐1.Yes<br>☐2.No<br>☐9.Don't know
38	Who did the participant's household borrow money from, from **the day your illness started**?	☐1.Family member ☐2.Friend<br>☐3.Informal money lender ☐4.Church/Mosque<br>☐5.Traditional leader<br>☐8.Other, specify: ____________________
39	Did the participant's household have to sell any item in order to raise money to pay for the participant treatment of this illness from **the day your illness started**?<br>*(If "No" and "Don't know" skip to Q.41)*	☐1.Yes<br>☐2.No<br>☐9.Don't know
40	What from the following did the participant's household have to do to make treatment from **the day the illness started** possible?<br>*(Select all that apply)*	☐1.Borrowed money<br>☐2.Sold livestock, specify: ____________<br>☐3.Sold belongings, specify: ____________<br>☐4.Sold property, specify: ____________<br>☐8.Other, specify: ________________<br>☐9.Don't know
41	Did the participant's household have to minimize or discontinue treatment from **the day your illness started** due to financial reasons?	☐1.Yes<br>☐2.No<br>☐9.Don't know

42	**Ç:** Do not include lost work and lost income related to illness for Q42 & Q43<br>Does any other household member have to stop either going to school, work or lost income in relation to the illness?<br>*(If “YES” enter number of people and days missed from school or work in Q.43)*<br>*(if “NO” or “Don’t know”, skip to Q. 44)*		☐1.Yes<br>☐2.No<br>☐9.Don’t know
43	How many people are affected and how many days have they missed school or work?	1.No. of pupils/students:_____ School days missed: _____<br>2.No. of Earners:______ Working days missed:______	

C: THE FAMILY BURDEN INTERVIEW SCHEDULE

Only for Caretaker/Next of Kin to Answer

Ç: Please first move away from the participant to a different place where the participant cannot hear your interview with the caretaker. Re-assure them nobody will know about their responses.

Please interview the caretaker of the participant on the following guidelines. You may probe further in order to assess a particular item if you feel it necessary. During the interview note your rating for each general category, as well as for each individual item, on the three-point scale 0, 1 and 2.

Caretaker/Next of Kin's relation to participant:____

	We are trying to assess the various difficulties felt by you as the caretaker of the participant, and will ask you few questions about these. Tell us to what extent the following **burdens** you from **the day the illness started**. Please do not hesitate to express your true feelings.	**No Burden**	**Moderate Burden**	**Severe Burden**
	**Financial Burden:**			
	**From the day illness started,** how will you rate the burden on you posed by the…			
44	Loss of participant's income (Has the participant lost his/her job? Stopped doing the work which s/he was doing before?; To what extent does it affect the family income?)	0 ☐	1 ☐	2 ☐
45	Loss of income of any other family member due to participant's illness? (Has anybody stopped working in order to stay home, lost pay, lost a job? To what extent are the family finances affected?)	0 ☐	1 ☐	2 ☐
46	Expenditure incurred due to participant's illness and treatment (Has s/he spent or lost money irrationally due to the participant's illness? How much has this affected the family's finances? How much has been spent on treatment, medicines, transport, and accommodation away from home and so on? How much has been spent on other treatments such as alternative/traditional healers? How has this affected family finances)	0 ☐	1 ☐	2 ☐
47	Expenditure incurred due to extra arrangements (For instance, any other relative coming to stay with the participant; appointing a nurse or servant; boarding out children. How have these affected the family finances?)	0 ☐	1 ☐	2 ☐
48	Loans taken or savings spent (How large a loan? How does the participant plan to pay it back? How much does it affect the family? Did the participant spend from savings? Were these used up? How much is the family affected?)	0 ☐	1 ☐	2 ☐
49	Any other planned activity put off because of financial pressure of the participant's illness (For instance, postponing a marriage, a journey or religious rite. How far is the family affected?)	0 ☐	1 ☐	2 ☐
	**Disruption of Family Routine Activities**			
	**From the day illness started,** how will you rate the burden on you posed by the…			
50	Participant not going to work, college, etc. (How inconvenient is this for the family?)	0 ☐	1 ☐	2 ☐
51	Participant not helping in the household work (How much does this affect the family)	0 ☐	1 ☐	2 ☐
52	Disruption of activities of other members of the family (Has someone spent time looking after the participant, thus abandoning another routine activity? How inconvenient is this?)	0 ☐	1 ☐	2 ☐
53	Participant's behaviour disrupting activities. (Participant insisting on someone being with him/her, not allowing that person to go out, etc.? Participant becoming violent, breaking things, not sleeping and not allowing others to sleep? How much does it affect the family?)	0 ☐	1 ☐	2 ☐
54	Neglect of the rest of the family due to participant's illness. (Is any other member missing school, meals, etc. How serious is this?)	0 ☐	1 ☐	2 ☐
	**Effect on Mental Health of Others**			
	**From the day illness started,** how will you rate the burden on you posed by the…			
55	Has any other family member sought help for psychological illness brought on by the participant's behaviour (for instance by the participant's drowsiness, confusion, or change in behaviour or his/her mental function)?^ How severe is this?	0 ☐	1 ☐	2 ☐
56	Has any other member of the family lost sleep, become depressed or weepy,	0	1	2

		Severely	A little	Not at all
	expressed suicidal wishes, become excessively irritable, etc.? How severe?	☐	☐	☐
	**Subjective Burden on the Family**			
	**From the day illness started,** how will you rate the burden on you posed by the…			
		**Severely**	**A little**	**Not at all**
57	How much would you say you have suffered owing to the participant's illness?	0 ☐	1 ☐	2 ☐

Note: The IVI acknowledges that the Family Burden Interview Schedule has been developed by Pai & Kapur, 1981; Ren H. et al., 2014

58	Can you tell us your motivation to be there for this person?	☐1.Religious ☐2.Financial ☐3.Family bondage<br>☐4.Friendship ☐8.Other, specify:__________________<br>☐9.Don't know

D	Interview scheduling and conclusion
**Ç:** Hand out the diary card to the study participant and explain to them how to record their activities relating to the study. Schedule a common date for the next meeting between the 12 to 14 days that is favourable to the participant.	
59	Schedule date of next interview: First schedule: **D**ate(dd/mm/yyyy): _____/_____/__________<br>Second schedule: **D**ate(dd/mm/yyyy): _____/_____/__________

E	CONCLUSION
☺That completes our interview for today. Thank you very much for taking the time to answer my questions.	

F	**Ç:** Complete the following yourself after finishing the interview.

Did you hand over and explained how to fill the diary card? ☐1.Yes ☐2.No

Interview completion date(dd/mm/yyyy): _____/_____/__________

Interview conducted by: _________________

Signature of Interviewer:_________________

Annex 7: SETA LT-SES Initial Interview for Controls Tool

F6.3: LT-SES Survey Form Neighbourhood Controls' Initial Interview

(Day 3 to 7: For Neighbourhood Control Participants)

F6.3 for neighbourhood controls at initial interview

Note to Interviewer
If you see ***"Ç",*** *that is a* ***note for you*** *to be read by yourself. If you see* ***"☺"*** *you should* ***read out loud*** *for the respondent or the caretaker/next of kin.*

Site/**C**ountry: ______________________________ / ____

Follow-up Schedule: ☐Day 3-7 (initial interview)

Interviewer's Name: ______________________________ **D**ate of Interview (dd/mm/yyyy): _____/_____/__________

Laboratory diagnosis: ☐**C**ontrol

Place of interview: ☐Health facility / ☐Home / ☐Other, specify________________________

If at health facility, indicate point of care: ☐Out-Patient / ☐In-Patient / ☐Invited visit for SETA research

PARTICIPATION ASSESSMENT SCREENING

**Ç*:*** **Is the participant selected as healthy controls for the study?**		
0	Correct?	☐1.Yes ☐2.No ☐9.Don't know
**Ç*:*** *If "**No**" or "**Don't know**", identify the right candidate to continue the interview.*		

Ç: *Use this instrument to interview an adult participant/caretaker/next of kin(in case of a minor or if health-impaired) that is familiar with the participant's wellbeing.*
☺ Now I am going to ask about the participant's **current** health state on general, physical, emotional, social wellbeing.

A: MEDICAL OUTCOME SURVEY FOR ADULT PARTICIPANT

Participant/Caretaker/Next of Kin(In case of minor or if health impaired)

	Ç: Read the response options for the study participants to choose one.<br>☺ Please select ONLY one response for each question and for sub-questions ONLY one for each line			
1	In general, would you say your health is:	☐1.Excellent ☐2.Very good ☐3.Good ☐4.Fair ☐5.Poor		
2	**Compared to four weeks ago**, how will you rate your health in general **now**?	☐1.Much better now than four weeks ago<br>☐2.Somewhat better now than four weeks ago<br>☐3.About the same<br>☐4.Somewhat worse now than four weeks ago<br>☐5.Much worse than four weeks ago		
	The following items are about activities you might do during a typical day. Does **your health now limit you** in these activities? If so, how much?	Yes, limited a lot	Yes, limited a little	No, not limited at all
3	**Vigorous activities**, such as running, lifting heavy objects, participating in strenuous sports	☐1.	☐2.	☐3.
4	**Moderate activities**, such as moving a table, pushing a vacuum cleaner, bowling, or playing golf	☐1.	☐2.	☐3.
5	Lifting or carrying groceries	☐1.	☐2.	☐3.
6	Climbing **several** flights of stairs	☐1.	☐2.	☐3.
7	Climbing **one** flight of stairs	☐1.	☐2.	☐3.
8	Bending, kneeling or stooping	☐1.	☐2.	☐3.
9	Walking **more than a mile**	☐1.	☐2.	☐3.
10	Walking **several blocks**	☐1.	☐2.	☐3.
11	Walking **one** block	☐1.	☐2.	☐3.
12	Bathing or dressing yourself	☐1.	☐2.	☐3.
	During **the past four weeks**, did you have any of the following problems with your work or regular daily activities **as a result of your physical health**?		**Yes**	**No**
13	Cut down the amount of time you spent on work or other activities		☐1.	☐2.
14	Accomplished less than you would		☐1.	☐2.
15	Were limited in the kind of work or other activities		☐1.	☐2.
16	Had difficulty performing the work or other activities (for example, it took extra effort)		☐1.	☐2.
	During **the past four weeks**, have you had any of the following problems with your work or other regular daily activities **as a result of any emotional problems** (such as feeling depressed or anxious)?		**Yes**	**No**
17	Cut down the **amount of time** you spent on work or other activities		☐1.	☐2.
18	**Accomplished less** than you would like		☐1.	☐2.
19	Didn't do work or other activities as **carefully** as usual		☐1.	☐2.

20	During **the past four weeks**, to what extent has your physical health or emotional problems interfered with your normal social activities with family, friends, neighbours or groups?	Not at all ☐1.	Slightly ☐2.	Moderately ☐3.	Quite a bit ☐4.	Extremely ☐5.

21	How much **bodily** pain have you had during the **past four weeks**?	None ☐1.	Very mild ☐2.	Mild ☐3.	Moderate ☐4.	Severe ☐5.	Very severe ☐6.

22	During **the past four weeks**, how much did **pain** interfere with your normal work (including both work outside the home and housework)?	Not at all ☐1.	A little bit ☐2.	Moderately ☐3.	Quite a bit ☐4.	Extremely ☐5.

	These questions are about how you feel and how things have been with you **during the past four weeks**. For each question, please give the one answer that comes closest to the way you have been feeling.						
	How much of the time during the past four weeks…	All the time	Most of the time	A good bit of time	Some of the time	A little bit of time	None of the time
23	Did you feel full of pep?	☐1.	☐2.	☐3.	☐4.	☐5.	☐6.

24	Have you been a very nervous person?	☐1.	☐2.	☐3.	☐4.	☐5.	☐6.
25	Have you felt so down in the dumps that nothing could cheer you up?	☐1.	☐2.	☐3.	☐4.	☐5.	☐6.
26	Have you felt calm and peaceful?	☐1.	☐2.	☐3.	☐4.	☐5.	☐6.
27	Did you have a lot of energy?	☐1.	☐2.	☐3.	☐4.	☐5.	☐6.
28	Have you felt downhearted and blue?	☐1.	☐2.	☐3.	☐4.	☐5.	☐6.
29	Did you feel worn out?	☐1.	☐2.	☐3.	☐4.	☐5.	☐6.
30	Have you been happy person?	☐1.	☐2.	☐3.	☐4.	☐5.	☐6.
31	Did you feel tired?	☐1.	☐2.	☐3.	☐4.	☐5.	☐6.
32	During **the past four weeks**, how much of the time has your **physical health or emotional problems** interfered with your social activities (like visiting friends, relatives, etc.)?	All of the time ☐1.	Most of the time ☐2.	Some of the time ☐3.	A little of the time ☐4.	None of the time ☐5.	
	How **TRUE** or **FALSE** is **each** of the following statements for you?	Definitely true	Mostly true	Don't know	Mostly false	Definitely false	
33	I seem to get sick a little easier than other people	☐1.	☐2.	☐3.	☐4.	☐5.	
34	I am as healthy as anybody I know	☐1.	☐2.	☐3.	☐4.	☐5.	
35	I expect my health to get worse	☐1.	☐2.	☐3.	☐4.	☐5.	
36	My health is excellent	☐1.	☐2.	☐3.	☐4.	☐5.	

Note: The IVI acknowledges that the RAND 36-Item Short Form Health Survey was developed at RAND as part of the Medical Outcome Study.

Scale and items: Physical functioning 3,4,5,6,7,8,9,10,11,12; Role limitation due to physical health 13,14,15,16; Role limitation due to emotional problems 17,18,19; Energy/fatigue 23,27,29,30; Emotional well-being 24,25,26,28,30; Social functioning 20,32; Pain 21,22; General health 1,33,34,35,36

B: FINANCIAL BURDEN

	**Financial Burden for Participants**<br><br>***Ç: Following questions do not include lost work and lost income related to caretaking and substitute labour during illness***	
37	During **the past four weeks,** did the participant's household have to borrow money from anyone in order to pay for treatment of **any illness**?<br>*(If "No" and "Don't know" skip to Q.39)*	☐1.Yes<br>☐2.No<br>☐9.Don't know
38	Who did the participant's household borrow money from **during the past four weeks**?	☐1.Family member ☐2.Friend<br>☐3.Informal money lender ☐4.Church/Mosque<br>☐5.Traditional leader<br>☐8.Other, specify: ____________________
39	Did the participant's household have to sell any item in order to raise money to pay for the participant treatment **during the past four weeks**?<br>*(If "No" and "Don't know" skip to Q.41)*	☐1.Yes<br>☐2.No<br>☐9.Don't know
40	What from the following did the participant's household has to do to make treatment of illness possible **during the past four weeks**?<br><br>*(Select all that apply)*	☐1.Borrowed money<br>☐2.Sold livestock, specify: ____________<br>☐3.Sold belongings, specify: ____________<br>☐4.Sold property, specify: ____________<br>☐8.Other, specify: ____________<br>☐9.Don't know
41	Did the participant's household have to minimize or discontinue treatment from **during the past four weeks** due to financial reasons?	☐1.Yes<br>☐2.No<br>☐9.Don't know

42	**Ç:** Do not include lost work and lost income related to the health condition for Q42 & Q43<br>Does any other household member have to stop either going to school, work or lost income in relation to the treatment or health condition?<br>*(If "YES" enter number of people and days missed from school or work in Q.43)*<br>*(if "NO" or "Don't know", skip to Q. 44)*	☐1.Yes<br>☐2.No<br>☐9.Don't know

43	How many people are affected and how many days have they missed school or work?	1.No. of pupils/students:_____ School days missed: _____<br>2.No. of Earners:______ Working days missed:______

C: THE FAMILY BURDEN INTERVIEW SCHEDULE

Only for Caretaker/Next of Kin to Answer

Ç: Please first move away from the participant to a different place where the participant cannot hear your interview with the caretaker. Re-assure them nobody will know about their responses.

Please interview the caretaker of the participant on the following guidelines. You may probe further in order to assess a particular item if you feel it necessary. During the interview note your rating for each general category, as well as for each individual item, on the three-point scale 0, 1 and 2.

Caretaker/Next of Kin's relation to participant:___________________________________

	We are trying to assess the various difficulties felt by you as the caretaker of the participant, and will ask you few questions about these. Tell us to what extent the following **burdens** you **during the past four weeks**. Please do not hesitate to express your true feelings.	**No Burden**	**Moderate Burden**	**Severe Burden**
	**Financial Burden:**			
	**During the past four weeks,** how will you rate the burden on you posed by the…			
44	Loss of participant's income<br>(Has the participant lost his/her job? Stopped doing the work which s/he was doing before?; To what extent does it affect the family income?)	0 ☐	1 ☐	2 ☐
45	Loss of income of any other family member due to participant's illness? (Has anybody stopped working in order to stay home, lost pay, lost a job? To what extent are the family finances affected?)	0 ☐	1 ☐	2 ☐
46	Expenditure incurred due to participant's illness and treatment<br>(Has s/he spent or lost money irrationally due to the participant's illness? How much has this affected the family's finances? How much has been spent on treatment, medicines, transport, and accommodation away from home and so on? How much has been spent on other treatments such as alternative/traditional healers? How has this affected family finances)	0 ☐	1 ☐	2 ☐
47	Expenditure incurred due to extra arrangements<br>(For instance, any other relative coming to stay with the participant; appointing a nurse or servant; boarding out children. How have these affected the family finances?)	0 ☐	1 ☐	2 ☐
48	Loans taken or savings spent<br>(How large a loan? How does the participant plan to pay it back? How much does it affect the family? Did the participant spend from savings? Were these used up? How much is the family affected?)	0 ☐	1 ☐	2 ☐
49	Any other planned activity put off because of financial pressure of the participant's illness<br>(For instance, postponing a marriage, a journey or religious rite. How far is the family affected?)	0 ☐	1 ☐	2 ☐
	**Disruption of Family Routine Activities:**			
	**During the past four weeks,** how will you rate the burden on you posed by the…			
50	Participant not going to work, college, etc.	0	1	2

	(How inconvenient is this for the family?)	☐	☐	☐
51	Participant not helping in the household work<br>(How much does this affect the family)	0<br>☐	1<br>☐	2<br>☐
52	Disruption of activities of other members of the family<br>(Has someone spent time looking after the participant, thus abandoning another routine activity? How inconvenient is this?)	0<br>☐	1<br>☐	2<br>☐
53	Participant's behaviour disrupting activities.<br>(Participant insisting on someone being with him/her, not allowing that person to go out, etc.? Participant becoming violent, breaking things, not sleeping and not allowing others to sleep? How much does it affect the family?)	0<br>☐	1<br>☐	2<br>☐
54	Neglect of the rest of the family due to participant's illness.<br>(Is any other member missing school, meals, etc. How serious is this?)	0<br>☐	1<br>☐	2<br>☐
	**Effect on Mental Health of Others**			
	**During the past four weeks,** how will you rate the burden on you posed by the…			
55	Has any other family member sought help for psychological illness brought on by the participant's behaviour<br>(for instance by the participant's drowsiness, confusion, or change in behaviour or his/her mental function)?^ How severe is this?	0<br>☐	1<br>☐	2<br>☐
56	Has any other member of the family lost sleep, become depressed or weepy, expressed suicidal wishes, become excessively irritable, etc.? How severe?	0<br>☐	1<br>☐	2<br>☐
	**Subjective Burden on the Family**			
	**During the past four weeks,** how will you rate the burden on you posed by the…			
		**Severely**	**A little**	**Not at all**
57	How much would you say you have suffered owing to the participant's illness?	0<br>☐	1<br>☐	2<br>☐

Note: The IVI acknowledges that the Family Burden Interview Schedule has been developed by Pai & Kapur, 1981; Ren H. et al., 2014

58	Can you tell us your motivation to be there for this person?	☐1.Religious ☐2.Financial ☐3.Family bondage<br>☐4.Friendship ☐8.Other, specify:___________________<br>☐9.Don't know

KNOWLEDGE AND PREVIOUS EXPERIENCE WITH TYPHOID
Health Risk Perception: *For Participant to Answer*

☺ I want to know what you think about typhoid fever.

D		
59	What comes to your mind when we say someone has "severe illness"? Tell me all that you think about this.<br><br>*(Ç: Do not read list. Check all that apply.)*	☐1. Hospitalization<br>☐2. Fainting<br>☐3. Confusion<br>☐4. Wound/bleeding<br>☐5. Repeated laboratory test<br>☐6. Repeated hospital visit<br>☐7. Death<br>☐8. Other, specify: ____________________<br>☐9. Don't know
60	Tell me how someone feels when they have typhoid fever (mention all you know)<br><br>*(Ç: Do not read list. Check all that apply.)*<br><br>*(If "Response 15" is mentioned, skip to Section G, Q.87)*	☐1.Headache ☐2.Exhaustion ☐3.Diarrhea<br>☐4.Rash ☐5.Constipation ☐6.Restlessness<br>☐7.Loss of appetite ☐8.Generalized aching<br>☐9.Cough ☐10.Nosebleeds<br>☐11.Mental confusion ☐12.Malaise ☐13.Nausea<br>☐14. Fever ☐15. Never heard about typhoid<br>☐98. Other, specify: ____________________<br>☐99. Don't know
61	Tell me all the ways that you believe typhoid fever is transmitted. (Mention all you know)<br><br>*(Ç: Do not read list. Check all that apply.)*	☐1. Polluted water/dirty water<br>☐2. Contaminated food<br>☐3. Flies<br>☐4. Lack of safe sanitation/proper disposal of excreta<br>☐5. Sleeping without mosquito net<br>☐6. Contact with patient/carrier<br>☐7. Mosquitoes<br>☐8. Other, specify: ____________________<br>☐9. Don't know
62	If someone is sick with typhoid fever, what do you believe are the best ways to cure this person?<br><br>*(Ç: Do not read list. Check all that apply.)*	☐1. No treatment is necessary<br>☐2. Antibiotics, specify: ____________________<br>☐3. Taking other medicine, specify: ________________<br>☐4. Drinking a lot of clean water<br>☐5. Injections<br>☐6. Changing one's diet<br>☐7. Cleaning house, environment or body<br>☐8. Religious healing<br>☐9. Herbal medicines<br>☐98. Other, specify: ____________________<br>☐99. Don't know

63	What are the best ways to avoid getting typhoid fever?<br><br>*(Ç: Do not read list. Check all that apply.)*	☐1. There is no way to prevent typhoid<br>☐2. Boil water before use/clean water<br>☐3. Control in-house water contamination (safe water storage)<br>☐4. Control in-community water contamination (safe water supplies)<br>☐5. Maintain clean and proper latrines<br>☐6. Maintain adequate waste disposal<br>☐7. Maintain cleanliness in food preparation and handling (e.g. handwashing)<br>☐8. Avoid food from street vendors<br>☐9. Clean the house or environment<br>☐10. Avoid contact with participants or carriers<br>☐11. Eat well-cooked food (including avoiding certain foods)<br>☐12. Get vaccinated<br>☐13.Maintain proper personal hygiene by hand washing<br>☐14. Take preventive medicine, specify: ___________<br>☐98. Other, specify: ____________________________<br>☐99. Don't know
64	Do you think typhoid fever is a severe illness?	☐1.Yes<br>☐2.No<br>☐9.Don't know
65	Who does typhoid affect more seriously?<br><br>*(Ç: Check only one.)*	☐1.Adults<br>☐2.School-age children<br>☐3.Preschool children<br>☐4.Infants<br>☐5.Elderly<br>☐6.Equally serious to all age groups<br>☐7.Not serious in any age group<br>☐8.Other, specify:____________________________<br>☐9.Don't know
**E**		
66	Do you think one gets typhoid fever in this community or somewhere else?	☐1.In this community<br>☐2.Somewhere else<br>☐8.Other, specify:____________________________<br>☐9.Don't know
67	Has the participant had typhoid fever within the past three years?<br>(If "No" or "Don't know" skip to Q.69)	☐1.Yes<br>☐2.No<br>☐9.Don't know
68	How many times has the participant had typhoid fever within the past three years?	_____ time(s)<br>9999=Don't know
69	Has anyone in your household ever died of typhoid fever?<br>(If "No" or "Don't know" skip to Q.72)	☐1.Yes<br>☐2.No<br>☐9.Don't know
70<br>&<br>71	70. How old were they when they died?<br>A. _____ year(s) 9999=Don't know<br>B. _____ year(s) 9999=Don't know<br>C. _____ year(s) 9999=Don't know	71. What year did they die? (*YYYY*)<br>D. ___________ 9999=Don't know<br>E. ___________ 9999=Don't know<br>F. ___________ 9999=Don't know
72	Has any adult now living in your household had typhoid fever within the past three years and recovered? (If "No" or "Don't know" skip to Q.75)	☐1.Yes<br>☐2.No<br>☐9.Don't know

73	How many adults now living in your household have had typhoid fever within the past three years and recovered?	______ Adult(s)<br>9999=Don't know
74	How many times has this adult had typhoid fever within the past three years and recovered?	

Relation	How old?	How many times?
A.		
B.		
C.		

75	Has any other child, now living in your household had typhoid fever within the past three years and recovered? (If "No" or "Don't know" skip to Q.78)	☐1.Yes<br>☐2.No<br>☐9.Don't know
76	How many other children now living in this household have had typhoid fever within the past three years and recovered?	______ Child(ren)<br>9999=Don't know
77	*(Ç: Complete the table by asking the respondent the questions in the top row).*<br>How many times has this child had typhoid fever within the past three years and recovered?	

Relation	How old?	How many times?
A.		
B.		
C.		

78 ☺ Can you tell me the number of people you know outside your family who have ever died of typhoid fever? ☐1.Yes ☐2.No ☐9.Don't know (If "No" or "Don't know" skip to Q.79)

Will be glad to know the relation and ages of those people to the participant.

Relation	How old?
A.	
B.	
C.	

AVERTIVE BEHAVIOUR

F		
79	Before the illness started, has any member of this household done anything special or spent any money on anything to specifically avoid getting infected with typhoid fever?<br>*(If "No" or "Don't know" skip to Section G, Q.87)*	☐1.Yes<br>☐2.No<br>☐9.Don't know

80	Which practices does your household observe to prevent themselves from typhoid fever?<br><br>*(Ç: Do not read list. Check all that apply.)*	☐1. Boil water before use or use bottled water<br>☐2. Wash raw vegetable thoroughly before use<br>☐3. Maintain cleanliness in food preparation and handling (e.g. handwashing with soap)<br>☐4. Eat well-cooked food (including avoiding certain foods)<br>☐5. Avoid food from street vendors<br>☐6. Seek doctors' advice on prevention measures<br>☐7. Report immediately to the hospital when sick<br>☐8. Vaccination<br>☐9. Control in-community water contamination (safe water supplies)<br>☐10. None of the above<br>☐98.Other, specify: ______________________________<br>☐99=Don't know
81	When do household members of the participant wash hands with soap?<br><br>*(Ç: Do not read list. Check all that apply.)*	☐1.During sickness<br>☐2.Before eating<br>☐3.After eating<br>☐4.Before cooking<br>☐5.After going to toilet<br>☐6.Upon returning home<br>☐7.When washing face<br>☐8.Never<br>☐98. Other, specify: ____________<br>☐99.Don't know
82	What kinds of vegetable washing habits does the participant's household observe?<br><br>*(Ç: Do not read list. Check all that apply.)*<br><br>*(If response is not "9 Do not wash", skip to Q.84)*	☐1.Washing with water only without additive<br>☐2.Washing vegetables with salt water<br>☐3.Washing vegetables with lemon juice<br>☐4.Washing vegetables with vinegar<br>☐5.Washing with mixture of water, vinegar, lemon juice<br>☐6.Just rinsing with water without soap or additive<br>☐7.Soak vegetables in plain water<br>☐8.Soak vegetables in additive mixture<br>☐9.**Do not wash**<br>☐98.Other, specify: ______________________________<br>☐99.Don't know
83	If respondent does not wash vegetables in Q.82, "response 9", ASK why not?	☐1.Feeling lazy<br>☐2. Too time consuming/inconvenient<br>☐3.I need to save water<br>☐4.No need to wash<br>☐5.I trust the shops wash well before selling<br>☐6.Small quantities of germs cannot cause harm<br>☐8.Other, specify: ______________________________<br>☐9.Don't know
84	How many times per week do your household members buy prepared (cooked) food from street vendors?<br>(If "no buying from street vendors" write "**0**" time(s) **BUT** if "Don't know" skip to Q.86)	______ time(s)<br><br>☐99.Don't know

85	If ZERO times in Q.84, ASK: Why don't the household members buy food from street vendors?	☐1.Too expensive<br>☐2.Too time consuming/inconvenient<br>☐3.Not available<br>☐4.Worried about typhoid<br>☐5.Worried about health risks<br>☐8.Other, specify: ____________________<br>☐9.Don't know
86	How early does one need to see a doctor when feeling sick with typhoid fever? Please select only one option.	☐1.No need to see a doctor<br>☐2.On the first day of symptom start<br>☐3.Three days after symptom start<br>☐4.One week after symptom start<br>☐8.Other, specify:_______________<br>☐9.Don't know

Socio-Economic Information

***Ç:** This information should be collected from the participant/caregiver in case of a child or when participant is unable to communicate.*

Socio-economic Indicators: *For adult participant or caretaker of a minor or any health impaired participant to answer*

G	☺ **We are almost done,** ***let me ask you few questions about yourself***	
87	What is the participant's level of education?<br>*(**Ç:** Ask proxy person if participant is less than 18 years)*	☐1.Never been to school<br>☐2.Preschool/Kindergarten<br>☐3.1-5 years of school (Primary)<br>☐4.6-9 years of school (JHS)<br>☐5.10-12 years of school (SHS)<br>☐6. Tertiary<br>☐7.Polytechnic /Undergraduate<br>☐8.Other, specify: ____________________<br>☐9.Don't know
88	Is the participant a pupil/student?	☐1.Yes<br>☐2.No, reason: _________________________<br>☐9.Don't know
89	Is the participant currently employed?<br>(If "No" or "Don't know" skip to Q.94)	☐1.Yes<br>☐2.No<br>☐9.Don't know
90	How many months in a year does the participant earn a wage?	__________ months<br>9999=Don't know
91	Does the participant get the same wage every month?<br>*(If "Yes" skip to Q.93)*	☐1.Yes<br>☐2.No<br>☐9.Don't know
92	In which months does the participant earn more?<br>*(**Ç:** Write all that apply and separate by commas)*<br>*(Refer to ** below for key)*	_________________________<br>99=Don't know
**	Response options for Q.92<br>**Months:** Jan=1, Feb=2, Mar=3, Apr=4, May=5, Jun=6, Jul=7, Aug=8, Sept=9, Oct=10, Nov=11, Dec=12	
93	Is the participant the only earning member of your household?<br>*(If "Yes" skip to Q.95)*	☐1.Yes<br>☐2.No<br>☐9.Don't know
94	Who else earns a wage in your/the participant's household?	1.No. of household members earning: __________<br><br>**In local currency**<br>2.Combined earning per day: Amount: __________
***Ç:** Please ask the respondent these questions in the table below on behalf of his/her **household**. If the respondent says "don't know", record (99) for Q.95. If the respondent refuses to answer, record (97)*<br><br>☺: I will like you to now tell me about participants work and how much you/s/he earn(s) per month?		

List	95***<br>**Occupation**	96.<br>**Earnings per month**<br>In local currency<br>DK=99999999
Participant		
Parent/caretaker (If different from participant)		
Head of household		

***	*Response option for Q.95 above*<br>**Occupation List**		
[1]<br>[2]<br>[3]<br>[4]<br>[5]<br>[6]<br>[7]<br>[8]<br>[9]	Student/Pupil<br>Retiree<br>Housewife<br>Professional<br>Unskilled office worker<br>Business owner<br>Farmer<br>Unskilled manual worker<br>Skilled manual worker	[10]<br>[11]<br>[12]<br>[13]<br>[14]<br>[15]<br>[97]<br>[98]<br>[99]	Street seller<br>Driver<br>Public servant<br>Petty trader<br>Fisherman<br>Service worker (eg. servant, cook, hotel or restaurant worker)<br>Refused to answer<br>Other, specify: ______________________________<br>Don't know

97	How many people live with the participant in the same household?<br>*(i.e. Number of all persons living in a house or a cluster of houses who share a common cooking pot)*	__________ person(s).<br>9999=Don't know
98	What is the main source of drinking water for members of your household?<br><br>*(If "Response 1, 2 or 9" is mentioned, go to "Q.99")*	**PIPED WATER**<br>☐1.Piped into dwelling/household connection<br>☐2.Piped plot/yard connection<br>☐3.Public tap/standpipe<br>☐4.**TUBEWELL/BOREHOLE**<br>**DUG WELL**<br>☐5.Protected well<br>☐6.Unprotected well<br>**WATER FROM SPRING**<br>☐7.Protected spring<br>☐8.Unprotected spring<br>☐9.**RAINWATER**<br>☐10.**TANKER TRUCK**<br>☐11.**CART WITH SMALL TANK/DRUM**<br>☐12.**SURFACE WATER(RIVER/DAM/LAKE/POND/ STREAM/CANAL/IRRIGATION CHANNEL)**<br>☐13.**BOTTLED WATER**<br>☐98.Other, specify: ______________________________

99	What kind of toilet facility do members of your household usually use?<br><br>*(If "Response 12" is mentioned, skip to "Q.102")*	**FLASH OR POUR FLUSH TOILET**<br>☐1.Flush to piped sewer system<br>☐2.Flush to septic tank<br>☐3.Flush to pit latrine<br>☐4.Flush to somewhere else<br>☐5.Flush, don't know where<br>**PIT LATRINE**<br>☐6.Ventilated improved pit latrine<br>☐7.Pit latrine with slab<br>☐8.Pit latrine without slab/open pit<br>☐9.COMPOSING TOILET<br>☐10.BUCKET TOILET<br>☐11.HANGING TOILET/HANGING LATRINE<br>☐12.NO FACILITY/BUSH/FIELD<br>☐98.Other, specify:_________________________
100	Do you share this toilet facility with other households?<br>*(If "No" skip to Q.102)*	☐1.Yes<br>☐2.No
101	How many households use this toilet facility?	☐1.Number of households if less than 10: ☐<br>☐2.10 or more households<br>☐9.Don't know
102	*(Ç: Read out each item and select the answer given. Multiple responses possible. Do not leave any item blank. If none, select 'No'.)*<br><br>Does your household have:<br>Electricity?<br>A Radio?<br>A television?<br>A mobile telephone?<br>A non-mobile telephone?<br>A refrigerator?	see below

☐1.Electricity	☐1.Yes	☐2.No
☐2.Radio	☐1.Yes	☐2.No
☐3.Television	☐1.Yes	☐2.No
☐4.Mobile telephone	☐1.Yes	☐2.No
☐5.Non-mobile telephone	☐1.Yes	☐2.No
☐6.Refrigerator/Fridge	☐1.Yes	☐2.No
☐7.Chair	☐1.Yes	☐2.No
☐8.Bed	☐1.Yes	☐2.No
☐9.Sofa	☐1.Yes	☐2.No
☐10.Cupboard	☐1.Yes	☐2.No
☐11.Table	☐1.Yes	☐2.No
☐12.Clock	☐1.Yes	☐2.No
☐13.Grain grinder	☐1.Yes	☐2.No
☐14.Cassette/CD player	☐1.Yes	☐2.No
☐15.Fan	☐1.Yes	☐2.No

103	What type of fuel does your household mainly use for cooking?<br><br>*(Ç: If the household uses more than one fuel for cooking, find out the fuel used most often. If any fuel other than the precoded ones is reported as being the main fuel used for cooking, select '98' and specify the type of fuel in the space provided)*	☐1.Electricity<br>☐2.LPG<br>☐3.Natural gas<br>☐4.Biogas<br>☐5.Kerosine<br>☐6.Coal, Lignite<br>☐7.Charcoal<br>☐8.Wood<br>☐9.Straw/Shrubs/Grass<br>☐10.Agricultural crop<br>☐11.Animal dung<br>☐12.No food cooked in household<br>☐98.Other, specify:__________________________
104	Main material of the floor.<br><br>*(Ç: Record observation; do not ask but check for yourself. If there is more than one kind of flooring material, record the main type of material (the material that covers the largest amount of floor space).)*	**NATURAL FLOOR**<br>☐1.Earth/Sand<br>☐2.Dung<br>**RUDIMENTARY FLOOR**<br>☐3.Wood planks<br>☐4.Palm/Bamboo<br>**FINISHED FLOOR**<br>☐5.Parquet or polished wood<br>☐6.Vinyl or asphalt strips<br>☐7.Ceramic tiles<br>☐8.Cement<br>☐9.Carpet<br>☐98.Other, specify:__________________________
105	Main material of the roof.<br><br>*(Ç: Record observation, in case of doubt asks the head of household. In case of different kinds of roofing material, record the main type of material that covers the largest amount of roof space.)*	**NATURAL ROOFING**<br>☐1.No roof<br>☐2.Thatch/Palm leaf<br>☐3.Sod<br>**RUDIMENTARY ROOFING**<br>☐4.Rustic mat<br>☐5.Palm/Bamboo<br>☐6.Wood planks<br>☐7.Cardboard<br>**FINISHED ROOFING**<br>☐8.Metal<br>☐9.Wood<br>☐10.Calamine/Cement fiber<br>☐11.Ceramic tiles<br>☐12.Cement<br>☐13.Roofing shingles<br>☐98.Other, specify:__________________________
106	Main material of the exterior walls.<br><br>*(Ç: Record observation, in case of doubt asks the head of household. In case of different kinds of exterior wall material, record the main type of material that covers the largest*	**NATURAL WALLS**<br>☐1.No walls<br>☐2.Cane/Palm/Trunks<br>☐3.Dirt

	amount of wall space.)	**RUDIMENTARY WALLS**<br>☐4.Bamboo with mud<br>☐5.Stone with mud<br>☐6.Uncovered adobe<br>☐7.Plywood<br>☐8.Cardboard<br>☐9.Reused wood<br>**FINISHED WALLS**<br>☐10.Cement<br>☐11.Stone with lime/Cement<br>☐12.Bricks<br>☐13.Cement blocks<br>☐14.Covered adobe<br>☐15.Wood planks/shingles<br>☐98.Other, specify:________________________
107	How many rooms in this household are used for sleeping?	Room(s): _____<br>☐99.Don't know
108	Does any member of this household own:<br><br>A watch?<br>A bicycle?<br>A motorcycle or motor scooter?<br>An animal-drawn cart?<br>A car or truck?<br>A boat with a motor?<br><br>*(Ç: Do not record children's bicycle as it is considered primarily as a toy.)*	☐1.Watch ☐1.Yes ☐2.No<br>☐2.Bicycle ☐1.Yes ☐2.No<br>☐3.Motorcycle/Scooter ☐1.Yes ☐2.No<br>☐4.Animal-drawn cart ☐1.Yes ☐2.No<br>☐5.Car/Truck ☐1.Yes ☐2.No<br>☐6.Boat with motor ☐1.Yes ☐2.No
109	Does any member of this household own any agricultural land?<br>*(If "No" or "Don't know", skip to "Q.111")*	☐1.Yes<br>☐2.No
110	How many hectares of agricultural land do members of this household own?<br>*(If 95 or more, select "Response 2".)*	☐1. Hectares: ☐☐.☐<br>☐2. 95 or more hectares<br>☐9.Don't know
111	Does this household own any livestock, herds, other farm animals, or poultry?<br>*(If "No" or "Don't know", skip to "Q.113")*	☐1.Yes<br>☐2.No

112	*(Ç: Read out each item and select the answer given. Multiple responses possible. Do not leave any item blank)*<br><br>How many of the following animals do this household own?<br><br>Cattle?<br>Milk cows or bulls?<br>Horses, donkeys, or mules?<br>Goats?<br>Sheep?<br>Chickens?<br><br>If none, enter '00'.<br>If 95 or more, enter '95'.<br>If unknown, enter '99'.	☐1.Cattle . . . . ☐☐<br>☐2.Cows/Bulls . . . . ☐☐<br>☐3.Horses/Donkeys/Mules . . . . ☐☐<br>☐4.Goats . . . . ☐☐<br>☐5.Sheep . . . . ☐☐<br>☐6.Chickens . . . . ☐☐<br>☐7.Pigs………... . . . . …. . ☐☐<br>☐8.Ducks……………………………. ☐☐<br>☐98.Other, specify:……………….....
113	Does any member of this household have a bank account?	☐1.Yes<br>☐2.No
114	What is the participant's source of drinking water outside home?	☐1.Kiosk/stall water seller<br>☐2.Water from street vendors<br>☐3.Bottled/Sachet water from local residents<br>☐4.Bottled/Sachet water from professional firms<br>☐5.Water from unprotected containers/barrels<br>☐6.Water from protected spring<br>☐7.Water from unprotected spring<br>☐8.Other, specify: ______________________________<br>☐9.Don't know
115	Does the participant have access to **a toilet outside home**?<br>(If "No" or "Don't know" skip to Q.117)	☐1.Yes<br>☐2.No<br>☐9.Don't know
116	Type of **toilet outside home**	**PUBLIC FLASH OR POUR FLUSH TOILET**<br>☐1.Public flush to pipe sewer system<br><br>**PUBLIC PIT LATRINE**<br>☐2.Ventilated improved pit latrine<br>☐3.Pit latrine with slab<br>☐4.Pit latrine without slab/open pit<br><br>☐5.PUBLIC HANGING TOILET/HANGING LATRINE<br>☐6.NO FACILITY/BUSH/FIELD<br><br>☐8.Other, specify:_________________________<br>☐9.Don't know
117	Which toilet does the participant usually use?	☐1.Home toilet<br>☐2.Outside toilet<br>☐3.Do not use<br>☐8.Other, specify:_________________________<br>☐9.Don't know

118	Who is the owner of the house the participant lives in?	☐1.Self ☐2.Parents ☐3.Family<br>☐4.Provided by employer ☐5.Rented<br>☐8.Other, specify:________________<br>☐9.Don't know
119	How much does the participant's household spend on average during the month?	**In local currency:**<br>____________<br>99999999=Don't know<br>88888888=No response
120	How much was the participant's household's own electricity related cost last month? Or, if the bill is being shared, how much was the participant's share?	**In local currency**<br>Household's own share of electricity bill: ________<br>66666666=We pay no monthly charge for electricity? It's included in the rent<br>99999999=Don't know/Not sure<br>88888888=No response
121	How much was the participant's household's telephone (land line and mobile) related cost last month?	**In local currency**<br>Household's own share of phone bill: ________<br>99999999=Don't know<br>88888888=No response

H	Interview scheduling and conclusion
**Ç:** Hand out the diary card to the study participant and explain to them how to record their activities relating to the study. Schedule a common date for the next meeting between the 12 to 14 days that is favourable to the participant.	
122	Schedule date of next interview: First schedule: **D**ate(dd/mm/yyyy): _____/_____/__________<br>Second schedule: **D**ate(dd/mm/yyyy): _____/_____/__________

I	CONCLUSION
☺That completes our interview for today. Thank you very much for taking the time to answer my questions.	

J	**Ç:** Complete the following yourself after finishing the interview.
Did you hand over and explained how to fill the diary card? ☐1.Yes ☐2.No<br><br>Interview completion date(dd/mm/yyyy): _____/_____/__________<br><br>Interview conducted by: ________________<br>Signature of Interviewer:________________	

Annex 8: SETA LT-SES Follow-up Interview for Cases and Controls Tool

F6.2: LT-SES Survey Form Cases and Controls' Follow-Up

(Day ____ to ____: For *S*. Typhi/Special Cases & neighbourhood Control Participants' Follow-Up till Day 360)

F6.2 for *S*. Typhi and special cases as well as controls during follow-up visits

Note to Interviewer
If you see ***"Ç",*** *that is a* ***note for you*** *to be read by yourself. If you see* ***"☺"*** *you should* ***read out loud*** *for the respondent or the caretaker/Next of kin.*

Site/**C**ountry: ______________________________ / ______________________________

Follow-up Schedule: ☐Day 12-14 ☐Day 28-30 ☐Day 90 ☐Day 180 ☐Day 270 ☐Day 360

Interviewer's Name: ______________________________ **D**ate of Interview (dd/mm/yyyy): _____/_____/__________

Laboratory diagnosis: ☐*S*. Typhi ☐Special cases ☐**C**ontrol

Place of interview: ☐Health facility / ☐Home / ☐Other, specify________________________

If at health facility, indicate point of care: ☐Out-Patient / ☐In-Patient / ☐Invited visit for SETA research

If In-Patient, name of admission ward: ___________________________________

Date of Admission: _____/_____/__________

Date of Discharge (dd/mm/yyyy): _____/_____/__________ [**Ç:** Look this up in the study facility record]

(Ç: In case the participant has died, enter the following and administer ***Form F7****):*

Date of Death (dd/mm/yyyy): _____/_____/__________ [**Ç:** Look this up in hospital record or ask household head]

PARTICIPATION ASSESSMENT SCREENING

**Ç:** *Is the participant the right person interviewed last time?*		
0	Correct?	☐1.Yes ☐2.No ☐9.Don't know
**Ç:** *If "**No**" or "**Don't know**", identify the right candidate to continue the interview.*		

**Ç:** *Use this instrument to interview an adult participant/caretaker/next of kin(in case of a minor or if health-impaired) that is familiar with the participant's wellbeing.*
☺ Now I am going to ask about the participant's **current** health state on general, physical, emotional, social wellbeing.

A: MEDICAL OUTCOME SURVEY FOR ADULT PARTICIPANT

Participant/Caretaker/Next of Kin(In case of minor or if health impaired)

	Ç: Read the response options for the study participants to choose one.<br>☺ Please select ONLY one response for each question and for sub-questions ONLY one for each line						
1	In general, would you say your health is:	☐1.Excellent ☐2.Very good ☐3.Good ☐4.Fair ☐5.Poor					
2	**From the day of last interview**, how will you rate your health in general **now**?	☐1.Much better now than the day of last interview<br>☐2.Somewhat better now than the day of last interview<br>☐3.About the same<br>☐4.Somewhat worse now than the day of last interview<br>☐5.Much worse than the day of last interview					

	The following items are about activities you might do during a typical day. Does **your health now limit you** in these activities? If so, how much?	Yes, limited a lot	Yes, limited a little	No, not limited at all
3	**Vigorous activities**, such as running, lifting heavy objects, participating in strenuous sports	☐1.	☐2.	☐3.
4	**Moderate activities**, such as moving a table, pushing a vacuum cleaner, bowling, or playing golf	☐1.	☐2.	☐3.
5	Lifting or carrying groceries	☐1.	☐2.	☐3.
6	Climbing **severa**l flights of stairs	☐1.	☐2.	☐3.
7	Climbing **one** flight of stairs	☐1.	☐2.	☐3.
8	Bending, kneeling or stooping	☐1.	☐2.	☐3.
9	Walking **more than a mile**	☐1.	☐2.	☐3.
10	Walking **several blocks**	☐1.	☐2.	☐3.
11	Walking **one** block	☐1.	☐2.	☐3.
12	Bathing or dressing yourself	☐1.	☐2.	☐3.

	**From the day of last interview**, did you have any of the following problems with your work or regular daily activities **as a result of physical health**?	**Yes**	**No**
13	Cut down the amount of time you spent on work or other activities	☐1.	☐2.
14	Accomplished less than you would	☐1.	☐2.
15	Were limited in the kind of work or other activities	☐1.	☐2.
16	Had difficulty performing the work or other activities (for example, it took extra effort)	☐1.	☐2.
	**From the day of last interview**, have you had any of the following problems with your work or other regular daily activities **as a result of any emotional problems** (such as feeling depressed or anxious)?		
17	Cut down the **amount of time** you spent on work or other activities	☐1.	☐2.
18	**Accomplished less** than you would like	☐1.	☐2.
19	Didn't do work or other activities as **carefully** as usual	☐1.	☐2.

20	**From the day of last interview**, to what extent has your physical health or emotional problems interfered with your normal social activities with family, friends, neighbours or groups?	Not at all ☐1.	Slightly ☐2.	Moderately ☐3.	Quite a bit ☐4.	Extremely ☐5.

21	How much **bodily** pain have you had **from the day of last interview**?	None ☐1.	Very mild ☐2.	Mild ☐3.	Moderate ☐4.	Severe ☐5.	Very severe ☐6.

22	**From the day of last interview**, how much did **pain** interfere with your normal work (including both work outside the home and housework)?	Not at all ☐1.	A little bit ☐2.	Moderately ☐3.	Quite a bit ☐4.	Extremely ☐5.

These questions are about how you feel and how things have been with you **from the day of the last interview**. For each question, please give the one answer that comes closest to the way you have been feeling.

	How much of the time from the day of the last interview…	All the time	Most of the time	A good bit of time	Some of the time	A little bit of time	None of the time
23	Did you feel full of pep?	☐1.	☐2.	☐3.	☐4.	☐5.	☐6.
24	Have you been a very nervous person?	☐1.	☐2.	☐3.	☐4.	☐5.	☐6.
25	Have you felt so down in the dumps that nothing	☐1.	☐2.	☐3.	☐4.	☐5.	☐6.

	could cheer you up?						
26	Have you felt calm and peaceful?	☐1.	☐2.	☐3.	☐4.	☐5.	☐6.
27	Did you have a lot of energy?	☐1.	☐2.	☐3.	☐4.	☐5.	☐6.
28	Have you felt downhearted and blue?	☐1.	☐2.	☐3.	☐4.	☐5.	☐6.
29	Did you feel worn out?	☐1.	☐2.	☐3.	☐4.	☐5.	☐6.
30	Have you been happy person?	☐1.	☐2.	☐3.	☐4.	☐5.	☐6.
31	Did you feel tired?	☐1.	☐2.	☐3.	☐4.	☐5.	☐6.
32	**From the day of last interview**, how much of the time has your **physical health or emotional problems** interfered with your social activities (like visiting friends, relatives, etc.)?	All of the time ☐1.	Most of the time ☐2.	Some of the time ☐3.	A little of the time ☐4.	None of the time ☐5.	
	How **TRUE** or **FALSE** is **each** of the following statements for you?	Definitely true	Mostly true	Don't know	Mostly false	Definitely false	
33	I seem to get sick a little easier than other people	☐1.	☐2.	☐3.	☐4.	☐5.	
34	I am as healthy as anybody I know	☐1.	☐2.	☐3.	☐4.	☐5.	
35	I expect my health to get worse	☐1.	☐2.	☐3.	☐4.	☐5.	
36	My health is excellent	☐1.	☐2.	☐3.	☐4.	☐5.	

Note: The IVI acknowledges that the RAND 36-Item Short Form Health Survey was developed at RAND as part of the Medical Outcome Study.

Scale and items: Physical functioning 3,4,5,6,7,8,9,10,11,12; Role limitation due to physical health 13,14,15,16; Role limitation due to emotional problems 17,18,19; Energy/fatigue 23,27,29,30; Emotional well-being 24,25,26,28,30; Social functioning 20,32; Pain 21,22; General health 1,33,34,35,36

B: FINANCIAL BURDEN

	**Financial Burden for Participants**<br><br>***Ç: Following questions do not include lost work and lost income related to caretaking and substitute labour during illness***	
37	**From the day of last interview,** did the participant's household have to borrow money from anyone in order to pay for the participant's treatment of illness?<br>*(If "No" and "Don't know" skip to Q.39)*	☐1.Yes<br>☐2.No<br>☐9.Don't know
38	Who did the participant's household borrow money from, from the **day of last interview**?	☐1.Family member ☐2.Friend<br>☐3.Informal money lender ☐4.Church/Mosque<br>☐5.Traditional leader<br>☐8.Other, specify: ____________________
39	Did the participant's household have to sell any item in order to raise money to pay for the participant treatment **from the day of last interview**? *(If "No" and "Don't know" skip to Q.41)*	☐1.Yes<br>☐2.No<br>☐9.Don't know
40	What from the following did the participant's household have to do to make treatment of illness from the **day of last interview** possible?<br><br>*(Select all that apply)*	☐1.Borrowed money<br>☐2.Sold livestock, specify: ____________<br>☐3.Sold belongings, specify: ____________<br>☐4.Sold property, specify: ____________<br>☐8.Other, specify: ________________<br>☐9.Don't know
41	Did the participant's household have to minimize or discontinue treatment from **day of last interview** due to financial reasons?	☐1.Yes<br>☐2.No<br>☐9.Don't know

42	**Ç:** Do not include lost work and lost income related to illness or health condition for Q42 & Q43<br><br>Does any other household member have to stop either going to school, work or lost income in relation to the illness or health condition?<br>*(If "YES" enter number of people and days missed from school or work in Q.43)*<br>*(if "NO" or "Don't know", skip to Q. 44)*	☐1.Yes<br>☐2.No<br>☐9.Don't know
43	How many people are affected and how many days have they missed school or work?	1.No. of pupils/students:_____ School days missed: _____<br><br>2.No. of Earners:______ Working days missed:______

C: THE FAMILY BURDEN INTERVIEW SCHEDULE

Only for Caretaker/Next of Kin to Answer

Ç: Please first move away from the participant to a different place where the participant cannot hear your interview with the caretaker. Re-assure them nobody will know about their responses.

Please interview the caretaker of the participant on the following guidelines. You may probe further in order to assess a particular item if you feel it necessary. During the interview note your rating for each general category, as well as for each individual item, on the three-point scale 0, 1 and 2.

Caretaker/Next of Kin's relation to participant:______________________________

	We are trying to assess the various difficulties felt by you as the caretaker of the participant, and will ask you a few questions about these. Tell us to what extent the following **burdens** you **from the day of last interview**. Please do not hesitate to express your true feelings.	**No Burden**	**Moderate Burden**	**Severe Burden**
	**Financial Burden:**			
	**From the day of last interview,** how will you rate the burden on you posed by the…			
44	Loss of participant's income<br>(Has the participant lost his/her job? Stopped doing the work which s/he was doing before?; To what extent does it affect the family income?)	0 ☐	1 ☐	2 ☐
45	Loss of income of any other family member due to participant's illness? (Has anybody stopped working in order to stay home, lost pay, lost a job? To what extent are the family finances affected?)	0 ☐	1 ☐	2 ☐
46	Expenditure incurred due to participant's illness and treatment<br>(Has s/he spent or lost money irrationally due to the participant's illness? How much has this affected the family's finances? How much has been spent on treatment, medicines, transport, and accommodation away from home and so on? How much has been spent on other treatments such as alternative/traditional healers? How has this affected family finances)	0 ☐	1 ☐	2 ☐
47	Expenditure incurred due to extra arrangements<br>(For instance, any other relative coming to stay with the participant; appointing a nurse or servant; boarding out children. How have these affected the family finances?)	0 ☐	1 ☐	2 ☐
48	Loans taken or savings spent<br>(How large a loan? How does the participant plan to pay it back? How much does it affect the family? Did the participant spend from savings? Were these used up? How much is the family affected?)	0 ☐	1 ☐	2 ☐
49	Any other planned activity put off because of financial pressure of the participant's illness<br>(For instance, postponing a marriage, a journey or religious rite. How far is the family affected?)	0 ☐	1 ☐	2 ☐
	**Disruption of Family Routine Activities**			
	**From the day of last interview,** how will you rate the burden on you posed by the…			

50	Participant not going to work, college, etc.<br>(How inconvenient is this for the family?)	0 ☐	1 ☐	2 ☐
51	Participant not helping in the household work<br>(How much does this affect the family)	0 ☐	1 ☐	2 ☐
52	Disruption of activities of other members of the family<br>(Has someone spent time looking after the participant, thus abandoning another routine activity? How inconvenient is this?)	0 ☐	1 ☐	2 ☐
53	Participant's behaviour disrupting activities.<br>(Participant insisting on someone being with him/her, not allowing that person to go out, etc.? Participant becoming violent, breaking things, not sleeping and not allowing others to sleep? How much does it affect the family?)	0 ☐	1 ☐	2 ☐
54	Neglect of the rest of the family due to participant's illness.<br>(Is any other member missing school, meals, etc. How serious is this?)	0 ☐	1 ☐	2 ☐
	**Effect on Mental Health of Others**			
	**From the day of last interview,** how will you rate the burden on you posed by the…			
55	Has any other family member sought help for psychological illness brought on by the participant's behaviour<br>(for instance by the participant's drowsiness, confusion, or change in behaviour or his/her mental function)?^ How severe is this?	0 ☐	1 ☐	2 ☐
56	Has any other member of the family lost sleep, become depressed or weepy, expressed suicidal wishes, become excessively irritable, etc.? How severe?	0 ☐	1 ☐	2 ☐
	**Subjective Burden on the Family**			
	**From the day of last interview,** how will you rate the burden on you posed by the…			
		**Severely**	**A little**	**Not at all**
57	How much would you say you have suffered owing to the participant's illness?	0 ☐	1 ☐	2 ☐

Note: The IVI acknowledges that the Family Burden Interview Schedule has been developed by Pai & Kapur, 1981; Ren H. et al., 2014

58	Can you tell us your motivation to be there for this person?	☐1.Religious ☐2.Financial ☐3.Family bondage<br>☐4.Friendship ☐8.Other, specify:___________________<br>☐9.Don't know

**D**	**Interview scheduling and conclusion**
**Ç:** Hand out the diary card to the study participant and explain to them how to record their activities relating to the study. Schedule a common date for the next meeting that is favourable to the participant.	
59	Schedule date of next interview: First schedule: **D**ate(dd/mm/yyyy): _____/_____/___________<br>Second schedule: **D**ate(dd/mm/yyyy): _____/_____/___________

**E**	**CONCLUSION**
☺ That completes our interview for today. Thank you very much for taking the time to answer my questions.	

**F**	**Ç:** Complete the following yourself after finishing the interview.

Did you hand over and explained how to fill the diary card? ☐1.Yes ☐2.No

Interview completion date(dd/mm/yyyy): _____/_____/__________

Interview conducted by: ________________

Signature of Interviewer:________________

Annex 9: SETA Cost of Death Interview Tool

Form F7: Death-Related Costs due to Illness

(Day 0 and beyond: At earliest after a study participant passes away)

F7 for all study arms, in case of death, during follow-up visits

Note to Interviewer
*If you see **"Ç",** that is a **note for you** to be read by yourself. If you see **"☺"** you should **read out loud** for the respondent or the caretaker/Next of kin.*

Site/**C**ountry: ______________________________/______________________________

Interview Schedule:

☐Day 0 (in case a Special Case died right upon enrollment)

☐Day 3-7 ☐Day 12-14 ☐Day 28-30 ☐Day 90 ☐Day 180 ☐Day 270 ☐Day 360

Recorder's Name:_________________________ **D**ate of Recording(dd/mm/yyyy):_____/_____/__________

Deceased was a: ☐***S***. Typhi Case ☐**S**pecial Case ☐***S***. Paratyphi Case ☐**i**NTS Case ☐**C**linical Enteric Fever Case ☐**C**ontrol

Place of **D**eath: ☐Health facility / ☐Home / ☐Other, specify: ___________________________________

If at health facility, indicate point of care: ☐Out-Patient / ☐In-Patient

If deceased was in In-Patient, name of admission ward: _________________________

Date of **A**dmission (dd/mm/yyyy): _____/_____/__________

Date of **D**eath (dd/mm/yyyy): _____/_____/__________ [**Ç:** Ask Next of Kin or Look this up in the study facility record]

Specific **C**ause of **D**eath (if known): ____________________ [**Ç:** Ask Next of Kin or Look this up in the study facility record]

Respondent's relation to the deceased: ☐1.Spouse ☐2.Mother ☐3.Father ☐4.Immediate family member ☐5.Friend ☐6.Neighbour ☐7.Hired labourer

***Ç:** The **deceased** was just enrolled or was the right person followed-up the last time*		
0	Correct?	☐1.Yes ☐2.No ☐9.Don't know
***Ç:** If "**No**" or "**Don't know**", **STOP** the interview of the Next of Kin/Household member.*		

***Ç:** Use this instrument to interview Next of Kin/Household member of the deceased person who is familiar with the participant before his/her death.*
☺ Now I am going to ask about the **Deceased person's** cost of death and **all funeral related costs incurred by the family**.

DETAILS OF EXPENDITURE DUE TO THE DEATH OF STUDY PARTICIPANT

Ç: ***Please collect only costs incurred after death.***

About how much did the family spend on each of the following during and after the death of the deceased study participant? For all that does not apply, mark N/A.

A	**Direct Costs of Death**<br>**Out-of-Pocket Medical and Non-Medical Care Expenditure after death**	
1	Age of the deceased (At the time of death)	years
2	Pathologist consultation cost	
3	Postmortem medical costs	
4	Body screening Xray charges	
5	Mortuary keeping costs	
6	Body handling medical expenses	
7	Handling material expenses	
8	Body transportation expenses	
9	Was any living household member (**companion**) with the corpse at the place of death? *(Skip to Q13, if "No" or "Don't know")*	☐1.Yes ☐2.No ☐9.Don't know
10	How many **companion(s)**?	
11	Food cost for **companion** (In local currency unit)<br>(if paid separately)	
12	Travel cost for **companion** (In local currency unit)<br>(One-way)	
13	Cost of funeral rites<br>*(In total, including cost of coffin, burial, funeral party, etc.)*	
14	Add-on and miscellaneous costs related to death	

Ç: If the deceased was earlier enrolled in the COI study, any data for responses to Q16 to Q20 in this Form F7 should be extracted from Form F2: COI Initial Interview "Section M".

B	**Indirect Costs of Death**<br>**Forgone Earnings of the Deceased and Companion(s)**	
15	Was the deceased earlier on enrolled in the COI Study before death?<br>*(Refer to **Participant/Caretaker Record, Register R1**)*<br>Ç: If "Yes", extract data for Q16 to Q20 from Form F2 Q97, Q101, Q102, Q103, Q104	☐1.Yes ☐2.No ☐9.Don't know
16	Was the deceased regularly employed before death?	☐1.Yes ☐2.No ☐9.Don't know
17	Was the deceased the only earning member of the household?<br>*(If "Yes" skip to Q.19)*	☐1.Yes ☐2.No ☐9.Don't know
18	How many other members of the deceased household earn a wage?	1.No. of household members earning: ___________

*ʢ: Please ask the respondent these questions in the table below on behalf of his/her **household and the deceased**. If the respondent says "don't know", record (99) for Q.19. If the respondent refuses to answer, record (97)*

☺: I will like you to now tell me about the occupation and regular monthly earnings of the deceased and his/her household members counted in Q18 above.

**List**	19*** **Occupation** *(See response options for *** above)*	20. **Earnings per month** In local currency DK=99999999
1.Deceased		
2.Parent/caretaker (If different from participant)		
3.Head of household		

***	*Response option for Q19 above.* **Occupation List**		
[1]	Student/Pupil	[10]	Street seller
[2]	Retiree	[11]	Driver
[3]	Housewife	[12]	Public servant
[4]	Professional	[13]	Petty trader
[5]	Unskilled office worker	[14]	Fisherman
[6]	Business owner	[15]	Service worker (eg. servant, cook, hotel or restaurant worker)
[7]	Farmer	[97]	Refused to answer
[8]	Unskilled manual worker	[98]	Other, specify: ______________________________
[9]	Skilled manual worker	[99]	Don't know

21	Did anyone have to cut back on his/her own usual activities in order to help in the funeral related activity? *(If "No" or "Don't know" skip to Section C, Q.33)*	☐1.Yes<br>☐2.No<br>☐9.Don't know
22	How many people helped in the funeral related activity?	__________ Person(s)<br>9999=Don't know

**Substitute number C-1**	23. # The deceased's relationship with the person who helped in the funeral related activities.	24. Is this person an adult, teenager, or a child? 1=Adult (17+) 2=Teenager (12-16) 3=Child (Less than 12) 9=DK	25. How many days did s/he lost in doing funeral related activities? *(Record number of days and/or number of hours as days/hours)* *Day*☐ *Hour*☐ 99=DK	26. Was this person paid to do funeral related activities? 1= Yes 2= No (Mark "0" in Q.27) 9 =DK (then mark "99999999" in Q.27)	27. How much was this person paid per day for doing the funeral related activities? 99999999=DK Enter **local currency** amount
**A**					
**B**					
**C**					
**D**					
**E**					
**F**					

#Response key for Q.23 above: 1. Spouse 2.Mother 3.Father 4.Immediate family member 5.Friend 6.Neighbour 7.Hired labourer 8.Can't say 98.Other, specify

Caretaker number C-2	28. Did this person who did the funeral related activity cut back on his or her own usual activities?<br><br>1= *Yes*<br>2 = No<br>9 =DK<br>*(If "No" or "Don't know" skip to Section C, Q.34)*	29. How many days did this person who did the funeral related activity cut back on his or her own duties? *(Record number of days and or number of hours as days/hours)*<br><br>*Day*☐ *Hour*☐<br>99=DK	30. Was this person who did the funeral related activity able to do:<br><br>1=Some of his/her own activities<br>2=None of his/her own activities<br>9=DK	31. What would this person who did the funeral related activity have been doing if he/she had not been preparing the deceased?<br><br>1 = Going to school<br>2 = Working on a farm<br>3 = Working at home<br>4 = Working for a wage<br>5 = Leisure time<br>8 = Other *(specify)*	32. *(If he/she would have worked for a wage)*<br><br>How much is this person who did the funeral related activity normally paid for one day's work?<br><br>DK=99999999<br>Enter **local currency** amount
**A**					
**B**					
**C**					
**D**					
**E**					
**F**					

**C**	**Financial Burden for Deceased Person's Household**<br><br>***Ç: Following questions do not include lost work and lost income related to caretaking and substitute labour during illness***		
33	**From the day of the participant's death,** did the participant's household borrow money from anyone in order to pay for funeral related costs?<br>*(If "No" and "Don't know" skip to Q.35)*		☐1.Yes<br>☐2.No<br>☐9.Don't know
34	Who did the deceased's household borrow money from, **for funeral related activities**?	☐1.Family member ☐2.Friend<br>☐3.Informal money lender ☐4.Church/Mosque<br>☐5.Traditional leader<br>☐8.Other, specify: ____________________	
35	Did the deceased's household have to sell any item in order to raise money to pay for any funeral-related cost **from the day of death**? *(If "No" and "Don't know" skip to Q.37)*		☐1.Yes<br>☐2.No<br>☐9.Don't know
36	What from the following did the deceased's household have to do to cater for the expenditure on funeral-related activities?<br><br>*(Select all that apply)*	☐1.Borrowed money<br>☐2.Sold livestock, specify: ____________<br>☐3.Sold belongings, specify: ____________<br>☐4.Sold property, specify: ____________<br>☐8.Other, specify: ________________<br>☐9.Don't know	
37	Did the deceased person's household have to cut down on living expenses from **day of death** due to financial reasons?		☐1.Yes<br>☐2.No<br>☐9.Don't know
38	Does any other household member have to stop either going to school, work or lost income in relation to the death of the study participant?<br>*(If "YES" enter number of people and days missed from school or work in Q.39)*<br>*(if "NO" or "Don't know", End the interview)*		☐1.Yes<br>☐2.No<br>☐9.Don't know

39	How many people are affected by the death of the study participant and how many days have they missed school or work?	1.No. of pupils/students:_____<br><br>2.No. of Earners:______	School days missed: ______<br><br>Working days missed:______

☺***: Thank you for the time. I very much appreciate it and please accept my condolence for your loss.***

Annex 10: SETA Clinical Recruitment Form Part 1 (Slected Questions only)

V. PRELIMINARY DIAGNOSIS

23. Indicate diagnosis made at first clinical assessment (check all that apply):

A. Malaria	☐ 1= Yes	☐ 2= No
B. Upper respiratory tract infection	☐ 1= Yes	☐ 2= No
C. Lower respiratory tract infection	☐ 1= Yes	☐ 2= No
D. Urinary tract infection	☐ 1= Yes	☐ 2= No
E. Gastrointestinal tract infection	☐ 1= Yes	☐ 2= No
F. Typhoid/Enteric fever	☐ 1= Yes	☐ 2= No
G. Skin infection	☐ 1= Yes	☐ 2= No
H. Sepsis	☐ 1= Yes	☐ 2= No
I. Cholecystitis	☐ 1= Yes	☐ 2= No
J. Appendicitis	☐ 1= Yes	☐ 2= No
K. Kidney stones	☐ 1= Yes	☐ 2= No
L. Central nervous system infection	☐ 1= Yes	☐ 2= No
M. Fever of unknown origin	☐ 1= Yes	☐ 2= No
N. Other (specify: _________________________)	☐ 1= Yes	☐ 2= No
